# Poster Presentations - Abstracts of the 26^th^ Annual Congress of the SBRA. São Paulo/SP - Brazil, 2022

**DOI:** 10.5935/1518-0557.20220044

**Published:** 2022

## P-01. Oocyte recovery through follicular flushing in low responders undergoing assisted reproduction technique. Retrospective case study

**Daniela Fink Hassan^1^, Karina Fattori^1^**

*^1^Centro de Reprodução Humana Wahib Hassan*

**Objective**: Follicular flushing or closed follicular lavage consists of injecting a specific medium into each follicle during oocyte uptake in *in vitro* fertilization, aiming to increase the rate of recovered oocytes and with the least damage to gametes. The objective of this retrospective descriptive comparative study was to evaluate the results of performing follicular flushing during the oocyte uptake process and to compare with conventional aspiration, in a sample of women with a low response, that is, ≤5follicles visualized on ultrasound and with a mean follicular diameter ≥14mm at the time of aspiration. We defined as the main objective the total number of oocytes collected in relation to aspirates and to compare results between aspiration cycles with and without flushing.

**Methods:** We used an electronic database of oocyte collection procedures performed at a human reproduction center between January 2021 and May 2022. At the beginning of the treatment, informed consents were obtained from the women, authorizing the use of data from the medical records for the research. Follicular aspiration was performed 36h after ovulation triggering under intravenous anesthesia. We selected 20 patients from a general group of 28, allocated according to age between 31 and 48 years and the number of follicles, paired into two groups, a group of 10 women who underwent follicular lavage with a 17G double lumen needle (K -OPSD-1735-BL, Cook, Australia), and another of 10 for conventional aspiration using a single 19G needle system (Wallace ONS 1733). 1.5 cc of washing medium (PBS, Ingamed^®^) was added to each punctured follicle to recover the oocyte-cumulus corona complex, repeating this process 3 to 4 times with suction at a pump pressure of 150 mmHg, always performed by the same operator. The final outcomes were defined as: the total cumulative rate of oocyte recovery, oocyte viability and the total operative time of the procedure in the groups with and without flushing. We performed a comparative descriptive analysis, defining the mean of the main outcome and the differences between the two groups.

**Results**: As a final result, the predominant mean age was 39.65±4.3 years. A total of 80 follicles were aspirated and a total of 59 oocytes recovered, an overall recovery rate of aspirated oocytes\follicles of 73.5%. The recovery rate of oocytes\follicles in the flushing group was 81.40% and 64.86% in the conventional aspiration group. Follicular flushing increased the oocyte retrieval rate by 16.54%. The mean operative time in the non-flushing group from 12.2±1.8 minutes increased to 39.5±4.6 minutes in the flushing group, an increase of 27.3 minutes in operative time. The viability of oocytes collected without flushing from 12.50% decreased to 11.43% using flushing.

**Conclusion:** We concluded that follicular flushing determined a higher overall rate of oocyte recovery, in low responders, in *in vitro* fertilization cycles, however the operative time of the procedure increased and oocyte viability reduced. The evidence was insufficient to allow conclusions regarding the effectiveness of the method and adverse events in view of the size, heterogeneity and bias of the sample.

## P-02. Reasons for disclosure to the child conceived by using donated gamete: when, how and why to tell

**Helena Prado Lopes^1,2^, Flávia Giacon^3^**

*^1^Instituto de Ginecologia da UFRJ, Hospital Moncorvo Filho - Setor de Reprodução Humana,*

*^2^Clínica Pró-Fértil - Centro de Medicina Reprodutiva*

*^3^Clínica de Reprodução Humana Mater Prime*

**Objective:** In recent years, the number of assisted reproduction treatments with the donation of gametes (ovules and/or sperm) has increased in Brazil. The use of donated gametes and embryos in assisted reproduction techniques (ART) makes it necessary to examine interests that involve relevant ethical and legal considerations, which include the autonomy and privacy rights of the intended parents, donors right to privacy and the right of the minors to know their genetic origin. In recent decades, the donor anonymity paradigm has changed in many countries, there has been a general trend towards the recognition of the rights of children born through ART with genetic material from donors, to learn their origins. Thus, the anonymity of gamete donors is no longer a unanimous rule at an international level and is one of the most controversial issues regarding the birth of children through donated eggs, sperm, embryos. Despite a growing consensus on donor-conceived offspring’s right to information about their genetic origin, disclosure to the child remains a challenge for many parents, particularly heterosexual couples. Research has consistently found that openness in donor conception families from an early age is optimal, and that disclosure positively impacts rather than threatens family relationships. Despite this, disclosure can be difficult for parents, particularly if they perceive an unsupportive social context. This article presents the research literature that tell us about disclosure patterns, the reasons for telling children about the way they were conceived, the best age to tell, and strategies for having this conversation with donor-conceived children. Although protecting children from possible harms was a major reason for infertile couples’ secrecy, based on the literature cited here in references, based on the literature read for this article, keeping this secret would not be always easy. Many individuals conceived through third-party participation are deprived of information that may be crucial to their future well-being for medical or psycho-social reasons. Additionally, long-term psychological counselling during and after the donation process is highly recommended.The anonymity of gamete donors in the context of medically assisted reproduction techniques (ART) and the right of the offspring to know their genetic or biological parents’ identity is a controversial and widely debated topic in the scientific literature. The decision to use a gamete donation in infertility treatment could have significant long-term psychological and social effects for all stakeholders involved. This study aims to raise the forms and not the formulas of how and when to reveal to the child about its origin.

**Methods:** This paper will focus on the psychosocial studies to find out whether there is evidence to show that disclosure of donor conception is in the best interests of donor offspring. The research literature that tells us about disclosure patterns, the reasons for telling children about the way they were conceived, the best age to tell, and strategies for having this conversation with donor-conceived children.

**Results:** The results of the present study indicated that most couples decided not to disclose the use of a donation procedure to their future child in order to protect the child from possible harms and also indicated that couples who chose not to disclose this information to others emphasized the idea of child protection from accidental disclosure as it could affect the child-parent relationship and create a lack of trust about the parents.

**Conclusions:** The results of the present study indicated that most couples decided not to reveal the use of gamete donation to their future child in order to protect the child from possible harm. The lack of a biological link and unprocessed feelings about it, can affect the relationship between parents and children. Mental health professionals working on this subject can play a fundamental role in guiding parents’ decisions about whether, when and how to tell their children about their origin. Psychological counseling during and after the gamete donation process is highly recommended.

## P-03. Semi-automated or automated FISH analysis for scoring aneuploidy in human sperm

**Daniela Borri^1^, Renata Kiyomi Kishimoto^1^, Giuliana Pandolfi^1^, Gilmara de Souza e Silva^1^, Roberta Maria Safranauskas^1^, Karla Pelegrino^1^, Elvira Deolinda Rodrigues Pereira Velloso^1^**

*^1^Sociedade Beneficiente Israelita Brasileira Albert Einstein*

**Objective:** Infertility affects 113 million people worldwide and one in six couples need the help of assisted reproduction techniques to have a child. Male factor infertility is present in ~40% of infertile couples. Sperm analysis has a central role in the diagnosis and treatment of male factor infertility. Classic sperm analysis includes measurement of semen volume, sperm concentration, percentage of motile sperm and the fraction of sperm with normal morphology. Genetic analysis, including the measurement of DNA damage and chromosome aberration (aneuploidy and structural aberrations) could be performed. The standard technique for measuring genetic heterogeneity in individual sperm within a population is Fluorescence *in situ* hibridization (FISH). Our study plans to study dissomy X, Y, 13, 18 and 21 chromosomes in a normal control population using an automated scan platform and compare the automated and semi- automated FISH analysis.

**Methods:** Normal semen parameters according to WHO 2010 criteria, DNA fragmentation and negative SARS-Cov-2 were considered in the control group. Two control groups were studied: samples from men 30- 39 and 40- 49-year-old. FISH technique was performed following standard protocol using commercial probes for X, Y, 13, 21 and 18 chromosomes. Analysis was carried out using an automated system. Scanning, relocation, capture, and fluorescence analysis were performed using a software for evaluating sperm nuclei, based on a flexible cell classifier system that can be calibrated by the user. An automated classifier was programmed to operationalize nuclei selection based on area, contour ratio, sphericity, and eccentricity following strict criteria. Abnormal scores were re-analysed manually (semi-automated analysis) by two experienced technicians applying the same strict criteria. Only sperm heads showing a regular contour and well-defined limits were evaluated and diploidy was defined as two signals from each chromosome in a set. Statistical analysis was performed, signal concordance (chromosomes) were evaluated using an intraclass correlation coefficient (ICC) with respective 95% confidence intervals (CI of 95%), between the automated and semi-automated methods and for the semi-automated scoring by two analysers and also calculated the repeatability between analysers.

**Results:** For each age group, 10 samples were analysed and for each sample, 2,000 nuclei were captured (1,000 for each probe) and the same nuclei were analysed by automated and semi-automated methods and by two technicians (table below). There was no concordance between automated and semi-automated analysis. In general, there was a good reproducibility in semi-automated analysis after training.

**Conclusion:** In our experience, the software was until now, unable to read the signal criteria stablished for all nuclei. Semi-automated FISH analysis relocating abnormal signals is a feasible approach for analysing numerical chromosomal aberrations in sperm.

**Table:** 

Chromosome	Age y.o (range)	Mean SemA 1	Mean Semi A 2	inter observers (CI 95%)	Rep Semi 1/2	Mean Aut	inter methods (CI 95%)
X	30-39 40-49	0.14 0.12	0.13 0.11	0.974 (0.905-0.993) 0.979 (0.922-0.995)	0.02 0.02	0.68 0.48	0.152 (-0.140-0.583) 0.094 (0-0.543)
Y	30-39 40-49	0.18 0.09	0.22 0.12	0.925 (0.713-0.981) 0.819 (0.454- 0.951)	0.05 0.05	0.96 1.13	0.067 (-0.143-0.464) 0 (0-0.433)
XY	30-39 40-49	0.11 0.4	0.12 0.39	0.966 (0.877-0.991) 0.973 (0.896-0.993)	0.02 0.05	2.69 4.82	-0.001 (-0.076-0.218) 0.034 (0-0.355)
13	30-39 40-49	0.18 0.25	0.20 0.26	0.955 (0.832-0.989) 0.970 (0.886-0.992)	0.03 0.04	3,15 4.57	-0.019 (-0.135-0.28) 0.000 (-0.056-0.175)
18/X	30-39 40-49	0.11 0.06	0.13 0.1	0.949 (0.811-0.987) 0.667 (0.154-0.903)	0.03 0.07	0.42 0.43	0.216 (-0.069-0.655) 0.105 (0-0.503)
18/Y	30-39 40-49	0.007 0.11	0.009 0.1	0.864 (0.559-0.964) 0.727 (0.212-0.925)	0.03 0.05	0.41 0.62	0.036(-0.060-0.294) 0.11(0-0.487)
21	30-39 40-49	0.24 0.36	0.27 0.35	0.871 (0.587-0.966) 0.963 (0.862-0.991)	0.06 0.04	1.32 1.98	0.009 (-0.226-0.44) 0.071(-0.104-0.432)

A: automated, SemiA: semi-automated analysis, Rep: repeatability.

## P-04. Clinical outcomes of a vitrified donor oocyte internacional program

**Milena Orso Ranzan^1^, Helena Vitoria Fauth^2^, Nicoly Abido Borilli^2^, Iáskara Vieira de Oliveira^1,2^**

*^1^Faculdade especializada na área da Saúde do Rio Grande do Sul - FASURGS Passo Fundo/RS*

*^2^Gerarte Clínica de Reprodução Humana Passo Fundo/RS*

**Objective**: International ovodonation is one of the alternatives that is gaining great focus in the area of assisted human reproduction. When female infertility is confirmed, one option is the method of ovoreception, which a woman can achieve pregnancy through a donated female gamete, the same can be selected in a Cell and Germinative Tissue Banks filtering donors phenotypically and genotypically. In the international ovonodation program, gametes are donated frozen by the vitrification method. Once the choice and the quantity of oocytes have been made, the cryopreserved samples are sent to the site of the procedure in another country. The objective of this work was to perform a data analysis of couples that chose the international ovodonation program in an assisted human reproduction clinic in the north region of Rio Grande do Sul, Brazil to verify the effectiveness of the treatment.

**Methods**: Fifteen medical records were analyzed, containing the evolution of each international ovodonation case from January/2018 to September/2020. We described the survival, fertilization, embryo development and blastocyst, implantation and pregnancy rates obtained in our program, and compared with the parameters found in the literature.

**Results**: In our ovodonation international program we obtained an occytes survival rate after warming of 83.1%, a fertilization rate of 81.9%, a cleavage implantation rate (D3) of 50.0% and a blastocyst implantation rate (D5) of 55.5%, which corresponded at the end of this analysis to a clinical data of 9 positive results from βHCG (pregnancy) with gestational sac development among the 15 medical records analyzed, which corresponded to a total implantation rate of 53.3%. In the literature, data were found in publications made between 2010-2018 with an average survival rate of oocytes after devitrification between 73.6% to 96.8%, fertilization rates between 66.1% to 79.2%, cleavage implantation rates (D3) between 30.0% to 34.0% and blastocyst implantation rates (D5) between 31.1% to 49.8%.

**Conclusion**: Based on these analyses compared with the rates described in the literature, the international ovodonation program demonstrates to be efficient for couples who choose this treatment.

## P-05. Morphologic embryo quality vs embryo ploidy, what we need to know?

**Edson Guimarães Lo Turco^1^, Fernanda Rodrigues Bernarde^1^, Mariana Moraes Piccolomini^2^, Oscar Duarte^2^, Lucas Yamakami^2^, Renato Tomioka^2^, Fernando Prado Ferreira^1,4^**

*^1^Universidade Federal de São Paulo, Departamento de Urologia, Divisão Reprodução Humana*

*^2^Lab For Life*

*^3^Clínica Neovita*

*^4^Universidade Federal de São Paulo - Departamento de ginecologia*

**Objective:** Evaluate the implications of the embryo quality in the outcome of embryo ploidy

**Methods**: Retrospective cohort study. In this study, 1082 patients were selected, over the period of 36 months (2019-2021) and divided into two groups according to the percentage of high- quality blastocysts. The high-quality blastocysts were defined by the blastocoel expansion into 4 or 5, and inner cell mass and trophectoderm in A or B (Gardner 2000). The groups were divided into patients with up to 20% of the high-quality embryos, Low-Quality Group (LQ-Group) with 682 patients, and patients with embryos with more than 50% of the high-quality embryos, High- Quality Group (HQ-Group), with 400 patients. The couples who underwent the same assisted reproduction treatment protocol were included in this study. The biopsy was performed on all embryos. Statistical analysis was performed by the student t-test, and the q-square test was applied for the categorical variables with the help of the SPSS software (V26). The age of the patients and the ovarian response were the same between both groups.

**Results:** The HQ-Group showed a higher fertilization rate (0.87±0.16 vs 0.80±0.19, *p*<0.05) and higher blastocyst formation (0.74±0.18 vs 0.28±0.27, *p*<0.05). Concerning normal embryos, the HQ-Group presented 346 euploid blastocysts and the LQ-Group presented 287 euploid blastocysts. We observed a higher proportion of euploid embryos in the high-quality group when compared to the low-quality group (41.6%±36.6 vs 34.56%±39.41, *p*<0.05). There was no statistical difference in the patient´s age, hormone levels, and ovarian response. In this way, we can observe a relationship between embryo quality and embryo euploidy.

**Conclusions**: Currently, morphological classification is the main tool used to select the embryo for transfer, although it is subjective and very dependent on the experience of the embryologist. This study shows that there is a relationship between embryo quality and embryo euploidy rate. In this way, this study can serve as a basis for physicians and patients to understand the relationship between the proportion of euploid embryos and the quality of embryos.

## P-06. Endometriosis causes changes in oocyte maturity but has no impact on embryo euploidy

**Fernanda Rodrigues Bernarde^1^, Marcelo Lucchesi Montenegro^2^, Alexander Kopelman^2^, Amanda Lino de Faria^2^, Silvia Morales Jau^2^ , Edson Guimarães Lo Turco^1^, Fernando Prado Ferreira^2^**

*^1^Universidade Federal de São Paulo, Departamento de Urologia, Divisão Reprodução Humana*

*^2^Clínica Neovita;*

**Objective:** The study aims to assess the impact of endometriosis on the proportion of genetically normal embryos.

**Methods:** Retrospective cohort study. In this study, we included 328 patients, divided into a study group (n=181) and controls (n=147). Data were collected between January 2019 and December 2021. The study group included patients with endometriosis without any other associated infertility factors. Controls were selected according to the following inclusion criteria: the presence of tubal factor, unexplained infertility, or presence of adenomyosis. All patients included in the study received the same controlled ovarian stimulation protocol and underwent the same assisted reproduction treatment with embryos transferred after the Preimplantation Genetic Test for aneuploidy (PGT-A). Statistical analysis of the data was performed using Student’s T-test and Chi-square test with SPSS software (V26). And an alpha of 5% was considered for the difference between the groups.

**Results:** In the comparison between groups, the variables age, LH, FSH, AMH, Estradiol, Progesterone, fertilization rate, rate of development to blastocyst, rate of chromosomally normal embryos, and rate of embryo implantation, did not show statistical difference. However, patients with endometriosis had a lower rate of stage MII oocytes (0.73±0.32 vs 0.87±0.45, p<0.002).

**Conclusions:** Endometriosis is a chronic disease and one of the main causes of female infertility that affects thousands of women worldwide. Many articles relate to endometriosis implantation failures and poor oocyte quality. However, the relationship of endometriosis with embryo development and possible genetic changes in these embryos is not well established. Thus, in this study, no relationship was observed between endometriosis and embryo development and in endometriosis and chromosomal changes in embryos. But oocyte rate in metaphase II in patients with endometriosis is lower when compared with the control group, as expected.

## P-07. Non-invasive preimplantational chromosomal testing of embryos

**Nicoly Abido Borilli^1^, Helena Vitoria Fauth^1^, Iáskara Vieira de Oliveira^1^**

*^1^Gerarte Clínica de Reprodução Humana/Passo Fundo-RS.*

**Objective**: The present study aims to describe what is non-invasive embryo analisys and the advantages, comparing euploidy and aneuploidy rates between PGT-A (Preinplantational Genetic Testing for aneuploidies) and NICS (Non-Invasive Chromosome Screening) techniques through a literature review.

**Methods**: The search for articles was carried out in 2 scientific databases and data of the objective, type of study, methods of inclusion and exclusion of articles, results and conclusions were related.

**Results**: In a literature analyzed, differences of 1.46% were found between the techniques, when comparing the results of euploidy and aneuploidy. Another study identified a difference of 2.13% for aneuploids embryos and 10.64% for euploids, 8.51% could not be analysed by the PGT-A technique. One of the articles showed a significant difference of 46.42% for aneuploids embryos and 37.5% for euploids embryos. Another research pointed out that 96.49% of the samples were successfull in the amplification of the genetic material. Other study pointed out an success of 99.10% and 94.80% of amplification at trofectoderm and non-invasive techniques, respectively. Furthermore, it demonstrated that media samples collected on the days 6/7 had a lower chance of having false positive results comparing day 5 sample (8.6% on day 6/7 and 29.6% on day 5).

**Conclusion**: Despite the advantages, non-invasive biopsy has a major disadvantage: the low amount of genetic material on culture media. It is important to emphasize that, even improving the non-invasive technique, PGT remains very important. However, non-invasive biopsy must continue to be studied, because patients who would not have the necessary qualifications to perform a traditional biopsy may have more assurance and security that their embryo will be genetically normal in case of implantation.

## P-08. Good prognosis couples. Is PGT-A useful?

**Amanda Amaral^1^, Victor Lazar^1^, Alecsandra Prado Gomes^1^, Tatiana Carvalho de Souza Bonetti^1,2^, Pedro Augusto Araújo Monteleone^1,3^**

*^1^Centro de Reprodução Humana Monteleone, Brazil*

*^2^Departamento de Ginecologia. Escola Paulista de Medicina da Universidade Federal de São Paulo*

*^3^Disciplina de Ginecologia - Departamento de Obstetrícia e Ginecologia. Faculdade de Medicina da Universidade de São Paulo, Brasil*

**Objective:** Under the broader field of assisted reproductive technologies, *in vitro* fertilization (IVF) is considered mainstream treatment. Nowadays, over 2.5 million cycles are being performed every year, resulting in over 500,000 deliveries. With technological advances and increasing demand for IVF, a number of interventions, referred to as “add-ons” have been introduced over the past decade. However, many such interventions were often introduced without proper validation. The most used add-on is the preimplantation genetic testing for aneuploidy (PGT-A), which is many times performed without appropriate indication. This study aimed to assess the clinical outcomes of IVF cycles of good prognosis couples who underwent standard protocols without adds-on. The primary outcome evaluated was the cumulative ongoing pregnancy after two embryo transfers per initiated cycle.

**Methods:** This is a retrospective cohort study evaluating good prognosis couples undergoing IVF cycles at a private assisted reproductive center. Couples who underwent IVF cycles between 2014 and 2020 with the following inclusion criteria were selected: Couples undergoing their first ICSI cycles with own oocytes, female age until 37 years old, at least 8 oocytes retrieved, no severe male factor (non-obstructive azoospermia and less than 1million of sperm concentration in ejaculated), no adds-on applied as PGT-A or other invasive procedure as Endometrial Receptivity assays. Women underwent standard ovarian stimulation with recombinant or urinary gonadotropin, GnRH agonist or antagonist for pituitary blockage and oocyte maturation trigger by recombinant hCG or GnRH agonist. Oocytes were fertilized by ICSI and embryos were cultured in a conventional or time-lapse incubator. Most of the transfers were performed at blastocyst stage (76.7%) and vitrification was used for embryo cryopreservation.

**Results:** From 3800 IVF cycles started in the study period, 410 cycles were selected for this study. The women’s age was 33.9±3.1 years old. Among the cycles evaluated, 27 had no embryo transfer (ET), 167 had the first fresh-ET and 216 had the first frozen-ET. The mean of embryos transferred was 1.4±0.5. The whole ongoing pregnancy rate after the first ET was 38%, as 29.9% (50/167) for fresh-ET and 44.4% (96/216) for frozen-ET. A second transfer was performed in 171 couples with a negative outcome and 64 ongoing pregnancies (37.4%) were obtained. The cumulative ongoing pregnancy rate per started cycle was 51.2% after two ET. If all couples with negative outcome after the first ET had done a second ET, the estimated cumulative ongoing pregnancy rate is 57% per initiated cycle.

**Conclusion:** In our routine, the PGT-A is an add-on used only in specific cases where karyotype alteration, recurrent miscarriage or implantation failure are present. Thus, we used the results published by The National Summary Report of Society for Assisted Reproductive Technology (SART) 2019 as a reference for treatments with PGT-A. The outcomes of this observational study confirmed a highly satisfactory clinical outcome for good prognosis couples without add-on. The SART 2019 shows a live birth rate of 44% in couples undergoing first ET, using (43.8%) or not PGT-A (43.9%) in more than 45 thousand cycles registered for women until 37 years old. The pregnancy rate after the first transfer observed in our study is very similar to SART register. However, it is important to note that the second transfer brings an important increment, attaining almost 60% of success, with no adds-on. Our results contradict the proposal of wide use of PGT-A to better select embryos and obtain a pregnancy in a shorter time. It also suggests that couples with good prognosis do not have any benefit of using PGT-A in their first IVF cycle. It is important to emphasize that the inherent risks and costs of these invasive procedures were avoided, with the same successful result.

## P-09. Ability of arrival to the blastocyst stage of embryos cultivated in single medium culture in patients with an average age of 38 years

**Cintia Pimentel Mangueira Teixeira^1^, Giovanna Marcela Barbosa^1^, Maria Luiza Silva Ricardo^1^, Wilson Jaccoud^1^, Marina Vicente Jaccoud^1^**

*^1^Fert-Embryo*

**Objective:**To evaluate the influence of single embryo culture medium on the rate of blastocyst formation in patients with advanced reproductive age.

**Methods:** Retrospective cohort study involving 84 patients with an average age of 38.54 years, who underwent an *in vitro* fertilization procedure at a clinic in the west region of the state of São Paulo, from January 2021 to May 2022. The Intracytoplasmic Microinjection technique was performed of Spermatozoa (ICSI) in 493 oocytes in metaphase II, cultured in a single medium culture at a temperature of 37ºC and 5% CO2. Once cleaved, the medium was renewed on day three of development and the embryos remained under evaluation between days five, six and seven of embryonic development, when classification into the blastocyst stage occurred.

**Results:** The results were expressed by the percentage of arrival to the blastocyst stage of each patient, followed by the quarterly average of the cases. In the first quarter of 2021 the blastocyst rate was 69.44%, in the second quarter 69.50%, in the third quarter the rate was 64.92% and in the fourth quarter of 2021, 59.51%. In 2022, in the first quarter the blastocyst rate was 58.62%, and in the second quarter it was 63.31%.

**Conclusion:** From the results obtained in the present study, we can conclude that there was a high rate of arrival at the blastocyst stage in women of advanced reproductive age. The single medium culture seems to have influenced the achievement of satisfactory results, reinforcing the importance of using culture methods that help in the best embryo selection within clinical practice.

## P-10. IVF add-ons: what do SisEmbrio-registered clinics say about it?

**Márcia Mendonça Carneiro^1,2^, Renata Bossi^1^, Tatiana Moreira^1^, Debora Alvarenga^1^, Ana Carolina Xavier^1^, Marcelo Lopes Cançado^1^, Rodrigo Hurtado^1,2^, Marcos Sampaio^1^**

*^1^ORIGEN Centro de Medicina Reprodutiva, Belo Horizonte-MG, Brazil*

*^2^Departamento de Ginecologia e Obstetrícia- Faculdade de Medicina da UFMG*

**Objective**: To evaluate XX^th^ SisEmbrio-registered fertility clinic websites in the State of Minas Gerais (MG), Brazil in order to find out how these clinics are advertising four common IVF add- ons: assisted hatching (AH), preimplantation genetic testing for aneuploidies (PGT-A), ERA test and sperm DNA fragmentation test.

**Methods**:The inclusion criteria were that the clinic: (i) offered IVF treatment and (ii) had a website. For each clinic, a record was made of whether each of the add-ons was advertised. If the clinic advertised the procedure, screenshots were taken of the webpages, including the claims made in relation to the add-ons. Advertisements were identified by a single reviewer. Categorization of claims were double-checked by a second reviewer.Statistical analyses were restricted to descriptive analyses of the frequency of each add-on, and the frequency of claims made in relation to their use. The folowing information on each add-on was colletced:Clinic offerered any of the mentioned add-on (yes or no); the website acknowledged that there could be a negative impact of an add-on (e.g:. a reduction in live birth rate; misdiagnosis risk); was the add-on offered as part of a package ( yes or no) or offered alone (stand alone);was there information on of effctiveness of the add-on in terms of: implanation rate(IR)(yes or no), pregnancy rate (PR)(yes or no) or live-birth rate (LBR)(yes or no); was there information on the scientifc uncertainty of the use of add-on and in the case of PGT-A did the clinic inform that the data obtained should be confirmed by prenatal diagnostic testing?Lastly, did the clinic offer any other add-on not mentioned above?

**Results:** There were 27 XX^th^ SisEmbrio-registered clinics in Minas Gerais ,26 private and one public which had no website. Two private clinics were also off-line. PGT was the most frequent available add-on (n=14; 58,3%) but it was not clear if they offered PGT-A or PGT for genetic diseases. Two clinics mentioned PGT-A was part of IVF but none informed on PR, IR nor LBR. The sperm DNA fragmentation test was presented in 6 websites but none reported on potential adverse effects nor success rates in terms of PR, IR or LBR. No information on the uncertainty of the benefit the test was provided. AH was found in 6 websites with no data on possible negative effects nor success rates (PR, IR or LBR) but 2 clinics admitted AH had uncertain benefits. As for the ERA-test, it was advertised in 5 websites with no information on its benefits on sucess rates but one clinic advised it could have a negative impact and one agreed its effectiveness was unclear. The cost was never pesented for any of the add-ons researched. Of note, three websites advertises superICSI, one ZYMOT, one ovarian tissue cryopreservation and one endometrial scrathcing.

**Conclusion:** Although not all IVF clinics in Minas Gerais advertise add-nos, the available information on their websites is heterogeneous. None of the websites reported the clinic’s pregnancy rate following the use of a certain add-on procedure nor did they inform on the current scientific uncertainty surrounding their use. The cost was never pesented for any of the add-ons researched. Unfortunately the information provided by these clinics is often innacurate and could result in comercial bias. As most people rely on the internet for information, it is of utmost importance that clinics offer scientific sound scientific data to their prospective patients and the general public as well.

## P-11. Patient’s profile evaluation and the factors that interfere in the quest for the treatment against infertility in a Human Reproduction Service

**Drauzio Oppenheimer^1^, Ana Carolina Vechi Siqueira^1^, Alexandra Abich^1^**

*^1^Faculdade de Medicina de Itajubá*

**Objective**: To asses patient’s profile who search for treatment against infertility and characterize the main factors that directly interfere and also restrict the access on a specialized services.

**Methods**: 50 questionnaires were answered by patients over 18 years old who are clinically followed up for infertility in a Human Reproduction Clinic, not bound by the Unified Health System, located in the city of São Paulo. Data were obtained through a questionnaire using the Google Forms platform. Among the variables are: age, race, education, monthly income, time to seek help and previous treatment, financial factor and desires related to the treatment.

**Results**: It was observed the prevalence of 44% Age Group between 36 and 40 years old, 66% White Race, 88% Education Higher Education and 30% with Monthly Income higher than R$8,000.00. In the sample, 88% of the patients already had a previous diagnosis. About the pathologies, we asked about the most reported in the literature, but 26% reported “other” as an answer, with 18% referring endometriosis and 16% causes related to the male factor, over the prevalence in national and international studies. Related to the tubal factor, 14%, ending below the literary prevalence, which is close to 40%. In relation to the time of pregnancy attempt, 60% of the patients have been trying for more than 3 years and 64% had already undergone previous treatment, and of these, 60% had already undergone *In Vitro* Fertilization previously. Moreover, 38% took 3 years or more to seek specialized help, similar to the literature. As for the financial factor, 30% have a monthly income above eight thousand reais, showing that access to the private sector is limited to a minority of the population. Moreover, 88% had higher education, reflecting the direct link between higher education and access to information, technologies, and availability of financial resources. Despite this, 74% of the patients mentioned the high cost as the greatest difficulty in the treatment, 72% thought of giving up because of this, and 48% resorted to loans. However, when asked if the greatest fear during treatment was not having the financial conditions to continue, most reported that the greatest fear was not having a successfully pregnancy.

**Conclusion**: There is a public health problem aggravated by public policies and the in access to specialized services, not widespread yet in Unified Health System. The late search for care shows scarce knowledge about health and infertility. The inaccessibility to specialized services makes more difficult the diagnosis and results in the failure of the pregnancy, since there are also several impeding factors, among them the high cost. This study showed that most had previously undergone a highly complex procedure, such as *In Vitro* Fertilization, demonstrating previous access to the service in this sample. The choice of the clinic was based on the most affordable cost for the woman and the couple; where the cost stood out as the greatest difficulty in the treatment and a significant portion of patients have already thought of giving up due to the financial factor. Thus, we observed that infertility therapies are still not accessible, where even a sample with higher socioeconomic status reports difficulty in access, reflecting the need for public and inclusive measures.

## P-12. The neglected father: evaluation of online resources for patient education on male preconception and fertility care in South America

**Márcia Mendonça Carneiro^1,2^, Raquel Lanna Cerqueira^3^, Marisa Mendonça Carneiro^4^**

*^1^ORIGEN Centro de Medicina Reprodutiva, Belo Horizonte-MG, Brazil*

*^2^Departamento de Ginecologia e Obstetrícia- Faculdade de Medicina da UFMG*

*^3^Faculdade FAMINAS Belo Horizonte-MG, Brazil*

*^4^Faculdade de Letras da UFMG, Belo Horizonte-MG*

**Objective**: investigate the availability of online patient education resources on male infertility well as male preconception care policies, guidelines, recommendations and services in South American countries using an electronic search of public available data on Google.

**Methods**: We performed an electronic search and investigation of preconception policy, guidelines, recommendations and services available to healthcare professionals and the general public in June 2022 in South America (n= 11 countries): Argentina, Bolivia, Brazil, Chile, Colombia, Ecuador, , Paraguay, Peru, Suriname, Uruguay and Venezuela. The availability of online patient education resources on male infertilty was evaluated by searching Google using advanced search setup for each country with the following words: male sterility; male factor; male infertility ; protocols; male reproductive health; male preconception advice; male preconception risk; male preconception visit; male conception; male reproductive advice. Data from the Latin American Registry (REDLARA, 2019) and the Latin American Federation of Societies of Obstetrics and Gynecology (FLASOG) as well as from each country Society of Urology was also obtained . The first 5 pages for each of the search terms presented by Google were evaluated. Private infertility clinics ads and websites were excluded from the analysis. No ethical approval as was obtained as we used only public available online information.

**Results:** Five Google pages on the topics were available for all countries except Paraguay (3 pages) and Suriname (1 page). Male preconception care guidelines provided by the government were only available in Uruguay and Venezuela whereas Argentina, Chile, Colombia and Uruguay had male infertility guidelines. Public provided patient information and education on male preconception care as well as infertility was available in Brazil and Uruguay. Only the Brazilian Urological Society displayed a protocol for managing male factor infertility. Even countries that had guidelines on male preconception care and infertility devoted little attention to the topic. In all countries the majority of information related to male infertility were provided by private fertility clinics and doctors as well as nonscientific publications and were not included in the analysis. Uruguay was the country that provided more information on male preconception care and male infertility.

**Conclusions**: There is a scarcity of public provided online information regarding male infertility in South American countries. The majority of governments and medical societies do not report specific guidelines. Governments and medical societies should join efforts to develop evidenced-based guidelines as well as scientific sound online patient information on male infertility and preconception care.

## P-13. What about the arrested embryos? Early morphokinetics parameters can predict non-developing blastocysts

**Mariana Nicolielo^1^, Catherine Kuhn Jacobs^1^, Fabiana Mendez^1^, Renata Erberelli^1^, Bruna Camillo Barros^1^, Ana Luiza Rossi de Castro Lopes^1^, Mauricio Barbour Chehin^1^, Eduardo Leme Alves da Motta^1,2^, José Roberto Alegretti^1^, Aline Rodrigues Lorenzon^1^**

*^1^Huntington Medicina Reprodutiva, Eugin Group*

*^2^Departamento de Ginecologia, Escola Paulista de Medicina, UNIFESP*

**Objectives:** Despite all technology in IVF laboratories, the majority of embryos created via assisted reproductive techniques subsequently arrest in extended culture, resulting in a low efficiency rate from fertilization to blastocyst stage. The aim of this study was to correlate morphokinetics parameters of developing and arrested embryos from patients using autologous and donor oocytes, in order to evaluate the contribution of advanced maternal age in those parameters.

**Methods:** This is a retrospective cohort study with 459 non-developing embryos from autologous cycles (n=242 patients) and 116 non-developing embryos from egg donation cycles (n=72 patients) performed between December 2017-2019 at Huntington Medicina Reprodutiva, Brazil. Maternal age, early morphokinetics parameters (time of pronucleous fading - tPNf, time to 2-cell - t2, t3, t4 and t5) were analyzed comparing to 1748 embryos that reached blastocyst stage from autologous cycles (n=430 patients) and 265 blastocysts from egg donation cycles (n=77 patients), cultured in Embryoscope Plus. Student t test was applied.

**Results:** Morphokinetic parameters between developing and non-developing embryos were distinct for almost all early time-points in autologous cycles: tPNf (23.83±3.15 versus 25.24±3.81, *p*<0.0001), t2 (27.01±3.91 versus 28.99±4.71, *p*<0.0001), t3 (36.96±4.68 versus 37.92±6.08, *p*=0,0001 and t4 (38.69±5.10 versus 40.79±6.70, *p*<0.0001). Only t5 parameter did not show significantly difference in autologous cycles (48.88±7.50 versus 48.88±9.26, *p*=0,5314). Maternal age was similar in autologous cycles (developing: 37.77±3.27 versus arrested: 37.93±4,24, *p*=0.1257). In egg donation cycles, the morphokinetic parameters that were different between developing and arrested embryos were t2 and t4 only (26.45±3.04 versus 28.57±5.50, *p*=0.0024; 38.66±5.43 versus 41.36±8.23, *p*=0.0093, respectively). tPNf, t3 and t5 were not statistically different in egg donation cycles in developing and arrested embryos (23.77±2.77 versus 24.61±4.90, *p*=0.0951; 36.72±4.85 versus 38.25±7.10, *p*=0.0745; 49.22±8.05 versus 51.67±11.74, *p*=0.2257, respectively). Mean age of egg donors in our program is 24.68±3.89.

**Conclusion:** Arrested embryos showed a delay in the first 48h of development. The most consisted parameters linked to embryo arrest were t2 and t4, that demonstrated difference in both autologous and egg donation cycles. In conclusion, early morphokinetics parameters can predict a good perspective of development embryos from those who will not reach blastocyst stage in autologous and donor cycles.

## P-14. Oocyte recipient patients: Tell or not telling the offspring the oocyte origin?

**Fernanda Kunrath Robin^1^, Sandra Maria Cezar Leal^2^, Denise Azambuja Zocche^3^, Gabriela Andrade^1^, Nilo Frantz^1^, Tagma Marina Schneider Donelli^2^**

*^1^Nilo Frantz Medicina Reprodutiva*

*^2^Universidade do Vale do Rio dos Sinos*

*^3^Universidade do Estado de Santa Catarina*

**Objective**: The number of *In vitro* fertilization cycles with donated oocytes significantly increased, specially by patients who postponement the motherhood, have genetic or oncological diseases or early ovarian failure. This treatment raises important psychological questions. This study objective is to increase knowledge of what these patients think about revealing to their offspring the use of heterologous (donated) oocyte.

**Methods**: We carried out qualitative retrospective research to understanding participants’ intention to reveal or not their offspring the use of heterologous oocytes. Heterosexual couples and single parents who underwent *in vitro* fertilization with donated oocytes from January 2017 to February 2020 were included. Due to the topic complexity and the emotional distress by participating in oocyte donation program, were included only participants who had a successful treatment. A total of 21 participants were drawn using the computer program (SorteioGo). Data collection was carried out through an electronic questionnaire. Data content analysis was based on Bardin (2011), in three phases: pre-analysis; material exploration; results treatment and interpretation. The encoding unit chosen was in accordance with the IRAMUTEq (Rautinaud, 2009) software standards, that was used for data processing and content analysis.

**Results**: Based on the patients’ answers, the results reinforce the lack of consensus on this subject and that there is no right or wrong about telling or not the offspring upon the use of heterologous oocytes. It is important to point out that some participants were still pregnant and others had children up to two years at the time they answered the questionnaire. Therefore, children already born do not yet have the necessary understanding of revelation. Part of the interviewed patients showed a willingness to reveal about the way their child was conceived (6 patients), some still have doubts about the disclosure (3 patients), but most believe there is no need to talk about it (12 patients). In addition to this, the patients have many psychological concerns with a variety feelings and emotions involved. Many doubts and uncertainties permeate this entire process and were reported in several responses.

**Conclusions:** The decision of tell or not the offspring about the origin of the oocyte is a challenge for oocyte donation cycles patients. Each family is inserted in a different context of life, which must be taken into account. Among those surveyed, the majority think that is no need to talk about it, nonetheless some patients think about counting; some have doubts and others have not thought about it.There is no rule as to whether or not to tell the offspring about their biological origins and this study results evidenced it. To increase knowledge about physiological dilemmas that oocyte donation cycles patients face during and after treatment can help future patients and involved staff to deal with it.

## P-15. Validation of non-invasive preimplantation genetic testing for aneuploidies (niPGT-A): comparative analysis of different kits and sequencing platforms

**Natalia Juliana Nardelli Gonçalves^1^, Camila Madaschi^1^, Priscila Fabiane dos Santos Beirigo^1^, Adriano Bonaldi^1^, Kalina Renata Naomi Endo^1^, Maria Fernanda Grillo Milanezzi^1^, Paulo Marques Pierre^1^, Patricia Guilherme^2^, Livia Vingris^2^, Edson Borges Jr^2^, Vinicius Bonatto Rosa^3^, Alessandro Schuffner^3^, Eduardo Gomes Sá^4^, Sebastião Evangelista Torquato Filho^4^, Janaína Mendes Maciel^5^, Ciro Dresh Martinhago^1^**

*^1^Diagnósticos da América - DASA*

*^2^Fertility Medical Group - São Paulo*

*^3^Conceber Clinic*

*^4^Bios Clinic*

*^5^Cenafert Clinic*

**Objective**: Preimplantation genetic testing (PGT) is the most reliable test to detect chromosomal aneuploidy in DNA from trophectodermal (TE) biopsy of embryos before implantation. A new non-invasive PGT-A (niPGT-A) method took advantage of the cell-free genomic DNA (cfDNA) present in human blastocoel fluid (BF) and provided the groundwork for the technique. Here we show the validation of three methods of niPGT-A in spent blastocyst media (SBM), in two NGS platforms. Consideration was given to technical limitations and potential of niPGT-A to provide reliable results.

**Methods:** Prospective niPGT-A validation was conducted in DASA - São Paulo - Brazil, 2021, with collaboration of four Brazilian IVF clinics. The zona pellucida of frozen/thawed (FT) and fresh blastocysts (FB) was lasered to release BF into culture medium and increase the concentration of embryo cfDNA. FT embryos on day 5/6 were cultured for 24h before SBM collection. In total, 99 SBM samples were collected from 71 unique embryos (40 FT and 31 FB), in tubes containing either buffer from Yikon Genomics or our “in house” buffer. Twenty-eight SBMs were collected with both buffers. All FT blastocysts had previous PGT-A diagnosis in TE samples. For FB, SBM collection and TE biopsy were performed on the same day. Twenty-six whole embryos (WE) were donated for testing, with couples informed consents. Three kits were used for whole genome amplification (WGA) and library preparation of cfDNA in SBM: NICSInst LNICS or INICS (Yikon Genomics), and ReproSeq PGS (ThermoFisher). LNICS and ReproSeq libraries were sequenced on GeneStudio S5 (ThermoFisher), using 530 chips, and INICS libraries on MiSeq (Illumina), using V3 kit. TE biopsies and WEs were processed with the ReproSeq kit. Data analysis was done using softwares ChromGo v.1.6 or Ion Reporter v5.12.3.0.

**Results:** All NGS runs showed excellent performances in terms of yield, valid reads rate, genome mapping and coverage, regardless of the kit or platform used. Averages of 1M and 135K reads per sample were achieved for Yikon and ReproSeq libraries, respectively, as expected. The overall clinical concordance between niPGT-A and PGT-A was 59~69%, and between niPGT-A and WE was 73~81%. ReproSeq showed higher rates, despite the lower number of reads. A possible reason is that ReproSeq was also used for analyzing TE and WE, thus all steps were the same for SBM, TE and WE, including templating on Ion Chef, sequencing on S5, and analysis in Ion Reporter, which may have reduced possible biases. The concordance rate of PGT-A x WE (85%) was higher than that observed for niPGT-A x WE, indicating that TE biopsy is still more accurate and, thus, more indicated for the analysis of aneuploidies. Comparison of 16 paired samples prepared with Yikon INICS and ReproSeq kits showed 81% concordant results. Divergences may be related to differences in sample collection, library preparation, sequencing platform, and total reads per sample. Furthermore, our data indicated a trend towards higher concordance of SBM x TE in FT blastocysts than FB (*p*-value: 0.048). One hypothesis is that the media from FT embryos might have more cfDNA since they were previously collapsed for TE biopsy.

**Conclusion:** Our data showed that analysis of aneuploidies by NGS is feasible in SBM samples from FT and fresh embryos, but also suggest that niPGT-A should be improved to achieve similar efficacy as PGT-A in identifying euploid embryos, potentially enhancing pregnancy outcomes. Further analysis is needed to improve our niPGT-A validation.

## P-16. Karyotype of miscarriage and maternal cell contamination

**Daniela Borri^1^, Maria Julia Lumi Watanabi^1^, Mauren Fernanda Moller dos Santos^1^, Elena Outon Alonso^1^, Amanda Aparecida Cardoso Coimbra^1^, Jason de Lima Silva^1^, Newton de Freitas Centuriao^1^, Margareth Afonso Torres^1^, Elvira Deolinda Rodrigues Pereira Velloso^1^**

*^1^Sociedade Beneficiente Israelita Brasileira Albert Einstein*

**Objective**: Chromosomal abnormalities are the most frequent cause of fetal loss in the first trimester of pregnancy, and miscarriage karyotyping is indicated as a part of investigation of recurrent spontaneous abortion. However, a female karyotype without abnormalities in product of conception may be the result of maternal cells analysis. This study aims to understand the profile of maternal cell contamination (MCC) performed by the PCR-STR (Polymerase Chain Reaction - Short Tandem Repeats) technique, in miscarriage tissue sent for karyotype study.

**Methods**: Karyotypic study of product of conception were performed using a standardized technique (G-banding), using 48 h (culture A), 72 h (culture B) and long-term (culture F) cultures of selected material (preferably villous). The MCC survey was performed by identifying the profile of 24 microsatellites from the mother’s genetic material (DNA from peripheral maternal blood) in the miscarriage tissue (DNA from the analysed culture), amplified using the globalfiler^®^ kit. The alleles were identified by an electrophoretic run in the ABI 3500 sequencer (applied biosystems). The results were analysed and the percentage of MCC was defined by the software chimermarker v.3.1.5 (softgenetics).

**Results**: From June 2021 to April 2022, 52 samples of product of conception with 46,XX karyotype were submitted to the PCR-STR technique to study MCC. In all, except four samples, PCR-STR technique was conclusive. MCC was present in 58% (7/12) in culture A, 80% (4/5) in culture B and 100% (31/31) in culture F (Table 1).

**Conclusion**: Evaluation of CCM by PCR-STR in abortion samples karyotyped as 46,XX is very important, especially in short culture cells (less than 72 h) in which heterogeneity in CCM can lead to misinterpretation of the etiology of fetal loss. For long-term cultures, the technique did not show benefits, as they all have MCC.

**Table 1:** 

Culture	Total	MCC	No MCC	Inconclusive
A	12	7	5	0
B	5	4	1	0
F	35	31	0	4

## P-17. The impact of chronic endometritis screening on clinical egg donation outcomes

**Fábio Vieira Vilela^1^, Ana Paula Melo Vianna^1^, Átila Sena Almeida^1^, Genevieve Marina Coelho^1^**

*^1^IVI (Instituto Valenciano de Infertilidade) - Salvador BA*

**Objective:** To evaluate the relevance of screening and treatment of Chronic Endometritis (CE) in the clinical results (clinical pregnancy and implantation rates) of Egg donation with embryo implantation failure.

**Methods:** Retrospective cohort study in which the clinical results of patients undergoing egg donation with a history of embryo implantation failure (EIF) at IVI Salvador, where ICSI cycles were performed from January 2019 to June 2022 were analyzed. Patients were divided into 2 groups: the 1st in which Chronic Endometritis was not investigated and the 2nd in which it was investigated through endometrial biopsy; diagnosed by pathological anatomy, immunohistochemistry with CD138 research, associated with pathognomonic findings on hysteroscopy; and treated with standard service antibiotic therapy. Data were verified regarding the age range of the egg recipient patients, ultrasound findings, hysteroscopy, endometrial thickness prior to embryo transfer, number of transferred embryos, embryonic quality, correlating with the clinical pregnancy and implantation rate. Data related to biochemical pregnancies were excluded.

**Results:** During the study period, 368 women were screened, of which 201 were excluded for not having a history of embryo implantation failure. This condition was observed in 167 patients, of which 5 were excluded due to biochemical pregnancies. In the study 162 patients were included and divided into 2 groups. 121 in group 1, in which Chronic Endometritis was not researched and 41 in group 2, in which the research was performed. In this group 2, only 20 patients were diagnosed with CE. In the general analysis of the data, it was observed that the mean age of the egg recipient patients was 44.0 (±4.1) years, 21.3% had anatomical changes on ultrasound and 38.2% had hysteroscopic findings, polyps were the most common (20.2%) in group 1. Mean endometrial thickness was 8.0 mm in group 1 and 7.9 mm in group 2. Two-embryo transfer was more frequent in the Endometritis group (65 .0% versus 57.9%); in contrast, the transfer rate of 1 embryo was higher in group 1 (42.1% versus 35.0%). Regarding the quality of the embryos, it was found that 95.9% of the transferred blastocysts were classified as of good/regular quality in group 1 and 95% in group 2. A very small percentage was found of embryos classified as of low quality, 4.2% in group 1 and 5% in group 2. Patients with positive screening for Chronic Endometritis had clinical pregnancy rates similar to patients who did not undergo this screening. The clinical pregnancy and implantation rate in group 1 was 54,5% e 43,4% respectively, while in group 2 it was 50.0% and 36.4%.

**Conclusion:** It was found that the screening and treatment of Chronic Endometritis has not been shown to impact the clinical outcomes of the Egg Donation treatments. The absence of statistical difference in the clinical results between the evaluated groups may corroborate the fact that the management of embryo implantation failure is multifactorial and a continuous challenge of assisted reproduction. However, prospective studies are needed to better elucidate the effect of screening for CE in this population, as well as better standardization of diagnostic methods.

## P-18. First trimester bleeding and clinical outcomes in patients submitted to *in vitro* fertilization

**A. V. Pereira Neto ^1^, G. M. Coelho^1^, D. P. Freitas^1^, A. P. M. Vianna^1^, M. Pereira^1^**

*^1^IVI Instituto Valenciano de Infertilidade - Salvador BA*

**Objective:** Evaluate the percentage of patients with genital bleeding after beta-HCG positive and what is the clinical outcome (Clinical Pregnancy, Abortion, Ectopic) in the first 8 weeks of gestation. Furthermore, stratify the findings in relation to the serum level of progesterone on the day of embryonic transfer.

**Method:** Retrospective study that analyzed the stimulation cycles performed in IVI Salvador between January 2020 and March 2022. All patients with positive beta-HCG after embryonic transfer of fresh *In Vitro* Fertilization (IVF) cycles, deferred transfer, with or without genetic study, and also patients of donated eggs or donated semen treatments were included. Patients with absence of any of the parameters were excluded from the study: Age, Cycle type, Progesterone dosage on the day of transfer, beta-HCG on day 12, characterization of bleeding and follow-up without clinical outcome.

**Results:** 416 patients with beta-HCG above 25mUi/ml were selected on the 12th day after implantation. Age ranged from 23 to 60 years. Of these 38 patients with fresh cycle (9.1%), and 378 in deferred cycle with frozen embryo transfer (90.8%). Among the frozen embryo transfers, the natural cycle was performed in 45 patients (10.8%). 254 (61%) patients with positive beta- HCG showed no bleeding. Among patients with bleeding (162), 30% presented as sludge type, 61% as bright red type and 8% with bleeding with clots. On the clinical outcomes of bleeding patients, 68.5% had clinical pregnancy while 51.6% progressed to abortion. Two patients (1.2%) had ectopic pregnancy and 16 (9.8%) had biochemical pregnancy. Of the 254 patients who did not present bleeding, 75.5% had clinical pregnancy and 37.3% progressed to abortion, even without bleeding. 29 patients had biochemical pregnancy and none of them had ectopic pregnancy. Regarding the degree of bleeding, of the 49 patients who presented blurry bleeding, 10.2% progressed to abortion. This number was higher in patients with live red bleeding (61.1%) and clots (8.6%), with a rate of 38.3% 3 57.1%, respectively. Regarding progesterone dosage, with a cutoff point of 9.2ng/ml, the percentage of patients with clinical bleeding (37.3% vs. 40%) and abortion outcome (26% and 28%) were similar.

**Conclusion:** The rate of genital bleeding in the first 8 weeks was similar to that observed in other studies, a percentage that is much higher than in spontaneous pregnancies, without an assisted method. Among these patients with bleeding, a higher percentage of abortion was observed (24.4% vs. 31.4%). When stratified by type of bleeding, the abortion rate increased in relation to the volume of bleeding. Additionally, when the serum level of progesterone is observed, there is no difference in the percentages of bleeding and loss.

## P-19. Evaluation of ploidy status in early day-4 blastocysts cultured in a time- lapse system

**Patricia Leme de Marchi^1^, Bruna C. Barros^1^, Catherine K. Jacobs^1^, Camila C. Moraes^2^, Ana Paula Reis^1^, Mauricio Barbour Chehin^1^, José Roberto Alegretti^1^, Aline Rodrigues Lorenzon^1^**

*^1^Huntington Medicina Reprodutiva, Eugin Group*

*^2^Pró-Criar Medicina Reprodutiva, Eugin Group*

**Objective:** Time-lapse (TL) is an imaging system that enables continuous monitoring of preimplantation embryo development, offering the opportunity to visualize time points and aspects of embryo morphology and morphokinetics in a non-invasive manner. Embryos with faster cleavage rate, especially during early divisions, are more predictive of livebirth. Embryo biopsy at an earlier time-point (day 4 of development) started to be considered only after the inclusion of a TL system in the laboratory routine. The aim of our study was to compare the euploidy rate between day 4 and day 5 blastocysts in the same cohort of biopsied embryos of a patient cycle.

**Methods:** Retrospective, case-control study including IVF cycles from patients that underwent preimplantation genetic testing for aneuploidy (PGT-A) by next generation sequencing (NGS) according to medical referral between April/2019 and January/2021 in a private ART clinic in Brazil. Two-hundred and two biopsied embryos from 44 patients that had in the same cycle full blastocysts biopsied at day 4 (D4) and D5 (default for embryo ploidy status) were included. Euploidy and pregnancy rates were compared between D4 and D5. Comparison statistical tests were performed accordingly to normal distribution of dataset. Fisher’s exact test were also used. A *p* value <0.05 was considered significant.

**Results:** Maternal mean age was 37.53±4.26 years old. Seventy-three blastocysts were biopsied at D4 (36.14%) and 129 blastocysts were biopsied at D5 (63.86%). Euploidy rate for D4 was 0.70±0.46 (70%) and for D5 was 0.50±0.51 (50%), *p*=0.02. Frozen embryo transfer was followed by 27/44 patients (61.4%). Positive pregnancy rate was similar between those that transferred a D4 or D5 euploid blastocyst (66.7% versus 41.7%, respectively, p=0.26). Maternal age in positive pregnancies after D4 or D5 euploid embryos were similar (35.75±1.58 and 37.60±4.51, p=0.34), as well in negative pregnancies (37.60±1.67 and 36.14±1.46, *p*=0.14).

**Conclusions:** Blastocysts biopsied at D4 had a higher euploidy rate when compared to D5 embryos biopsied from the same patient cycle. Pregnancy rates had not differ from D4 or D5 euploid embryo transfers. The presence of a D4 blastocyst in a patient’s cohort of embryos eligible to transfer, additionally to morphology and other morphokinetics parameters, may be considered for embryo ranking before uterine transfer.

## P-20. Fertility in women undergoing chemotherapy treatment: a mapping review

**Ana Karoline Machado da Rosa^1^, Luis Pedro Bernardi^1^, Fabiano Hahn Souza^1^**

*^1^CoreBox Medical Communications*

**Objective**: Assess the difference in fertility between healthy women and women undergoing chemotherapy with alkylating agents.

**Methods:** A mapping review of the medical literature was performed in PubMed/Medline, Web of Science, and EMBASE to identify studies that evaluated the impact of chemotherapy with alkylating agents (carboplatin, cisplatin, oxaliplatin, and cyclophosphamide) on female fertility. Articles published in English were searched on the above mentioned databases up to June 2022 using controlled vocabulary terms (MeSH or Emtree) and free text words. Only articles having the following characteristics were considered: observational or experimental clinical studies of the association between alkylating agents and female fertility.

**Results:** A total of 5543 articles were located according to the methodology used, and duplicates and unavailable ones were removed, in association with filters and application of eligibility criteria, leaving 16 articles for analysis. The impact of chemotherapy on a woman’s fertility depends on her age and the types and doses of drugs used. Therefore, studies have shown that anti-cancer therapy with treatments combined with at least one alkylating agent is often a cause of premature ovarian failure and infertility, three of them reported a decrease in anti-Müllerian hormone. Even with the presentation of these outcomes, pregnancies have been reported, showing that chemotherapy can affect fertility but does not prevent women from having children and does not result in deleterious effects on pregnancies.

**Conclusion**: Oocyte cryopreservation and fertility-sparing surgery are good alternatives for cancer treatment patients with alkylating agents to minimize infertility issues.

## P-21. Correlation between sperm concentration and sperm DNA fragmentation index

**Fabrício Sousa Martins^1^, DarleteLima Matos^1^, Karla Rejane Oliveira Cavalcanti^1^, Lilian Maria da Cunha Serio^1^, Daniel Paes Diogenes de Paula^1^**

*^1^Fertibaby Ceará*

**Objective**:To Investigate the relationship between sperm DNA fragmentation and sperm concentration.

**Methods**:In the present study carried out from January 2020 to March 2022, 70 samples of semen from patients investigated for infertility were analyzed and sperm analysis parameters were correlated with the result of the DNA fragmentation test. Patients were instructed to maintain a period of abstinence from 48 to 72 hours and to collect their samples of semen, by masturbation, inside a sterile plastic container. The samples were submitted to liquefaction at 37°C for 30 minutes, and the following parameters of semen analysis were evaluated: volume of semen, percentage of sperm motility, and total concentration of motile sperm. Patients who had a sperm concentration higher than 16.0 million/ml or progressive motility higher than 30% (WHO normality criteria, 2021) and strict morphology higher than 4.0% were considered normal. For the evaluation of sperm fragmentation, we used the sperm chromatin dispersion test (SCD) which consists of fixing spermatozoa in agarose gel and submitting them to lysis solutions for DNA exposure. Spermatozoa with intact DNA had an expressive halo formed around the head while those with fragmented DNA had a small halo around the head or no halo. Samples with values greater than 30% fragmentation were considered altered. After this analysis, the patients were divided into two groups: normospermic and oligospermic and correlated with the results of the fragmentation tests in each group. Results were represented by means (± standard deviation, SD) and compared using Student’s t test for independent samples. A significance level of 0.05 (*p* = 5%) was adopted.

**Results**:The value of sperm DNA fragmentation was higher in normospermic samples becuase 64% of patients with sperm concentration within the normal range, those called normosperms, had high or altered sperm DNA fragmentation. On the other hand, only 24% of patients with low sperm concentration, that is, oligozoospermic patients, had high or altered sperm DNA fragmentation. Thus, we cannot correlate low sperm concentration with high DNA fragmentation index.

**Conclusion**: In this study, the relationship between sperm concentration and fragmentation rate was investigated, and no positive correlation was found between these parameters, since a higher rate of DNA fragmentation was found in patients with normal sperm concentration. Scientific reports claim that it is common for apparently normal semen to have subtle defects that are not found in basic semen analysis such as sperm analysis. Sperm DNA damage is associated with low pregnancy rates and an increased risk of miscarriage, making laboratory investigation of DNA integrity necessary even in patients with normal seminal parameters.

## P-22. Oncofertility: An academic guide for students and lecturers of health and biological sciences

**Adriana Bos-Mikich^1^, Bianca Suzin Santos^1^, Rossana Colla Soletti^1^**

*^1^Universidade Federal do Rio Grande do Sul*

**Objective**: The academic guide on oncofertility is aimed at lecturers and students who are involved in providing and spreading healthcare information, within the university community and outside its gates. Given the lack of assessment to fertility information, particularly for young people suffering from a cancer, the academic guide main goal is to make students and lecturers aware of oncofertility, giving special attention to fertility preservation for children, adolescents and adults of reproductive age with cancer.

**Methods**: The guide is being developed as a website, in which different titles and subtitles are presented, together with illustrations and videos with legends. Images are presented with a transcript displayed bellow the illustration. A forum is provided for students and lecturers to interact among them and with the authors of the website. When finished, the site will be host at the Federal University of Rio Grande do Sul institutional site and at free platforms such as Wordpress. The explanatory texts in each Subtitle are presented as blogs in Portuguese language, with room for commentaries at the end of each subject. The main titles include: Introduction to oncofertility, risks to fertility after cancer treatments, tumor types and infertility risks, fertility preservation for children, pre-pubertal and pos-pubertal adolescents and adults of reproductive age (these topics divided in male and female cancer patients), established methods and experimental procedures for FP and psychological aspects of FP and quality of life of the patients. All information provided is based on a preliminary scientific literature review. The pertinent information is collected and described by two PhD lecturers with extensive experience in oncology pharmacology and human embryology. Infographics are made using Canvas program. Links will be provided to access scientific societies, oncolofgy referral services in Brazil and abroad, annual meetings of societies engaged in the study of Oncofertility and\or FP, together with pertinent scientific references identified in literature.

**Results**: The development of the Oncofertility guide identified more than 30 articles containing information eligible for description and inclusion in the blogs. The website logo and infographics were created using Canvas program to depict the importance of discussing fertility issues with cancer patients and methods for fertility preservation for each category of patient (male, female, children, adolescent and young adults). The project has received good acceptance in the academic community and a scholarship was granted for a graduation student take part in the creation of the website.

**Conclusions**: Considering that the University environment represents a solid ground to disperse information and scientific knowledge, the development of an academic guide on oncofertility represents an important tool to increase the level of consciousness on the impact that cancer and its treatments may have on patients´ fertility. The guide describes in a simple way, scientific information to be used by lecturers in their classes and by students during their academic years to enhance their knowledge on fertility issues that may impact the quality of life of people inside and outside the academic milieu suffering from cancer.

## P-23. Serum estradiol to follicle ratio as oocyte maturity marker and outcome predictor of *in vitro* fertilization cycles

**Luiza Mezzomo Donatti^1^, Carolina Lumertz Martello^1^, Gabriella Mamede Andrade^1^, Fernanda Robin^1^, Nilo Frantz^1^**

*^1^Nilo Frantz Reproductive Medicine*

**Objective:** Patients undergoing *in vitro* fertilization (IVF) treatments are submitted to different controlled ovarian stimulation (COS) protocols to achieve a livebirth child. Ovarian response to COS depends on patient’s characteristics, such as ovarian reserve and body mass index (BMI), and the clinicians use follicles diameter measure to follow the responsiveness. Serum estradiol (E2) level can also be used to control COS duration and to determine the oocyte pickup (OPU) time since it reflects, in general, follicle size and maturity. Serum E2 to follicles number ratio could be an interesting predictor parameter to obtain high-quality oocytes from COS. This study aimed to evaluate if the E2/follicle number ratio (E2/fol) could be used as an oocyte quality marker and to predict laboratory outcomes and pregnancy rates in IVF cycles.

**Methods:** Retrospective cohort study including 587 IVF cycles in a single reproductive medicine center from 2016 to 2021. COS was made by GnRH agonist/ antagonist protocol, under the patient’s characteristics, using gonadotrophins and trigger administration was made 36 hours before OPU. Patients enrolled in this study collected a blood sample for E2 dosage on the morning of trigger administration day and serum E2 levels (pg/mL) were evaluated using an enzyme-linked immunosorbent assay technique (Minividas, Biomérieux). E2/fol was determined as the relation of serum E2 level on the trigger day to either the number of punctured follicles during OPU. From the initial 587 IVF cycles, 143 cycles presented fresh embryo transfer (ET). Patients were divided into four E2/fol groups: A, <100; B, 100-200; C, 201-300; and D, >300. Age, BMI, oocyte maturity rate, oocyte immaturity rate, fertilization rate, top-quality blastocyst rate, and pregnancy rates were compared among the groups. Top- quality blastocysts were evaluated for inner cell mass (ICM) and trophectoderm (TE), respectively, and were classified as Bl1 (AA, AB, or BA), and Bl2 (BB or CB). Total ovarian stimulation days and total gonadotrophin dosage used were also compared among groups.

One-way ANOVA was applied to compare the groups and *p*-value ≤0.05 was considered statistically significant.

**Results:** In group D, in which the patients have the higher E2/fol ratio, the data analysis showed a lower percentage of M1 (3.28%, *p*= 0.0303) and GV (6.24%, *p*= 0.0064) oocytes recovered during OPU. Additionally, group D patients presented a higher pregnancy rate (57%, *p*= 0.004) among the groups despite being older (37.3 years old, *p*= 0.0031). Interesting that patients from group A were younger (33.8 years old, *p*= 0.0031), with a greater BMI (26.72, *p*= 0.0002), and received a lower total gonadotrophin dose during COS (2134 IU, *p*= 0.0002) compared to higher E2/fol groups. Others laboratory outcomes evaluated as total ovarian stimulation days (11.1 x 11.4 x 11.2 x 11.2 days, *p*=0.8898), oocyte maturity rate (78.9%x79.5% x 81.5%x82.9%, *p*=0.297), fertilization rate (75.4% x 74.3% x 73.2% x 76.2%, *p*=0.6313), and top quality blastocysts formation rate (59.9% x 67.6% x 62.2% x 65.5%, *p*=0.4035) did not differ between the four study groups.

**Conclusion:** IVF treatment success depends on various aspects, but an optimal COS is essential to provide good quality oocytes. Younger patients with a greater BMI tended to have lower E2/fol and it seems to affect the maturity of oocytes retrieved, suggesting poor follicle maturation. Clinicians can delay trigger administration based on E2 dosage and ultrasound follicle measurement in light of to achieve higher E2/fol and better quality oocytes. Our results showed that E2/follicle ratio >300 had the lowest oocyte immaturity rate and the highest clinical pregnancy rate after fresh ET, suggesting that high E2/fol could be used as an oocyte maturity marker and an IVF outcome predictor.

## P-24. Sperm Parameters Alterations On Patient Post COVID-19 Infection

**Mariana Mitiko Aseka Garcia^1^, Regis Yukio Cho^2^, Kahisa Natiele Fontana Dal Toso^2^, Tiago Cesar Mierzwa^2^, Débora Scaraboto^2^, Isadora Terumi Saruhashi^2^, Lidio Jair Ribas Centa^2^**

*^1^Universidade Federal do Paraná*

*^2^Androlab*

**Objective:** Case report of a patient that after infected with COVID-19 presented alterations in spermogram parameters.

**Methods:** Literature review on the influence of COVID-19 and febrile syndrome on male human reproduction, and a case study of patient with alteration in spermogram parameters.

**Results:** A 53-year-old male patient seeking *in vitro* fertilization (IVF) treatment underwent a sperm analysis on August 9, 2021 with a sperm count of 4.6 million/mL and 10% progression in sperm motility. On February 9, 2022, he was diagnosed with COVID-19 trough detection of viral antigen, presenting, among the symptoms, febrile syndrome (FS). On March 26, 2022, the patient underwent semen collection to start the IVF procedure, however the first sample had a sperm count of 0.4 million/mL and 0% progression, a second sample was collected and presented 0.2 million/mL and 0% progression, the procedure was canceled due to lack of viable sperm. Following treatment, sperm analysis was performed 2 months after FS on April 13, 2022 with a count of 1.5 million/mL 5% progression and, on May 25, 2022, 3 months after FS, the count was 0.9 million/mL and 4% progression.

**Conclusion:** Exposure of the testis to moderate to high temperatures affects testicular function and may cause disruption of sperm production. Studies evaluated the impact of febrile episodes on seminal parameters, it was found alterations in sperm count, motility and morphology after febrile episodes in patients with chickenpox, with recovery of these parameters after 4 weeks of temperature normalization and normal sperm count after 8 weeks. These findings are consistent with those reported by several studies.

The most common cause of fever in infections is Influenza, but in COVID-19 infections, up to 98.6% of patients may have fever, among other symptoms such as fatigue, dry cough, myalgia and dyspnea. Studies indicate that the infection of cells by SARS-CoV-2 involves the presence of angiotensin-converting enzyme 2 receptor (ACE2) and breakage of the spike protein of the virus by TMPRSS2. Although the expression of ACE2 in Sertoli and Leydig cells is high, the expression of TMPRSS2 is very low, and in spermatogonial stem cells the expression is even lower, indicating low susceptibility to viral entry into cells. This is consistent with the results presented, which did not find the virus in the semen of most patients in one study. The patient in this report had progressive spermatozoa prior to COVID-19, severe asthenozoospermia after the fever, followed by presence of motile spermatozoa after a period of new sperm production. This may indicate that COVID-19, like other infections that alter body temperature, influences sperm production temporarily after fever episodes. Most studies have not found SARS-CoV-2 in the male reproductive tract, although some studies have reported orchitis or hormonal changes as consequence of infection (impaired spermatogenesis). This likely indicates that sperm changes in patients infected with SARS-CoV-2 are likely to be due to increased body temperature, as seen in other systemic infections.

## P-25. Blastocyst rate with D3 evaluation versus without D3 Evaluation

**Kahisa Natiele Fontana Dal Toso^1^, Débora Scaraboto^1^, Isadora Terumi Saruhashi^1^, Regis Yukio Cho^1^, Viviane Margareth Scantamburlo Niehues^1^, Lidio Jair Ribas Centa^1^**

*^1^ Androlab*

**Objective:** To evaluate and compare the blastocyst rate of embryos which were exposed to evaluate the cleavage stage versus embryos that were let in the incubator until Day 5.

**Methods:** Retrospective study, performed at the ART (Assisted Reproduction Technology) center Androlab in Curitiba, Brazil, between january/2021 and june/2022. All ICSI cycles performed at this center were included. All embryos were cultured with continuous medium in an incubator without a time lapse system. The data were divided into two groups. The group A from embryos that were not evaluated on cleavage stage and group B with embryos that were evaluated on Day+3 or Day+2. Time lapse systems has been proved to increase success rates by avoiding the exposure of embryos in the ART laboratory. Although many ART centers does not have time lapse systems, alternatives can be used to minimize the negative effects of assessment to these embryos, like not evaluating them at cleavage stage.

**Results:** As shown below in Table 1 a total of 1525 embryos were analyzed, 756 from group A and 769 embryos from group B. In group A the blastocyst rate was significantly higher: 49% against 41% in group B (*p*=0.004). The rate of poor quality blastocysts were 11% in group A and 16% in group B (*p*=0.031). The confidence interval=95%.

**Conclusion:**Not evaluating the embryos at cleavage stage was beneficial by improving blastocyst rates (*p*=0.004) and decreasing poor quality blastocyst rate (*p*=0.031). This way we can avoid exposure of embryos and reduce the manipulation, increasing the rate and quality of blastocysts.

**Table 1:** Comparison of results on Group A (with evaluation only at blastocyst stage) and Group B (with evaluation on cleavage stage)

	Group A	Group B	*p* value
Maternal age	36.7	38	-
no. of Embryos	756	769	-
Blastocyst rate	49%	41%	0.004
Poor quality blastocyst rate	11%	16%	0.031

## P-26. Effect of the time of ICSI after the trigger in fertilization rate

**Kahisa Natiele Fontana Dal Toso^1^, Isadora Terumi Saruhashi^1^, Débora Scaraboto^1^, Viviane Margareth Scantamburlo Niehues^1^, Regis Yukio Cho^1^, Franciane Poletto Obara^1^, Ana Carolina Possebom^1^, Lidio Jair Ribas Centa^1^**

*^1^ Androlab.*

**Objective:**To evaluate if there is an optimal time post trigger to perform ICSI considering fertilization rate.

**Methods:**A single-center retrospective cohort analysis performed at the ART (Assisted Reproduction Technology) center Androlab in Curitiba, Brazil, was performed including 2099 oocytes from 385 ICSI cycles from January/2021 until July/2022. All cycles of ICSI performed at this center were included. Regarding the time interval between ovulation triggering and oocyte injection, four categories were considered: <37.5h; 37.6h-38.5h; 38.6h-39.5h; >39.6h. Oocyte retrieval was routinely performed 35 hours post triggering. In all cases, denudation was performed immediately prior to injection.

**Results:**As shown in the Table 1, when ICSI was performed less than 37.5 hours after the trigger (n=131), the fertilization rate found was 79% (n=103). When ICSI was performed 37.5 to 38.5 hours after triggering (n=1436), the fertilization rate found was 85% (n=1220). For those oocytes whose ICSI was performed 38.6 to 39.5 hours after triggering (n=485) the fertilization rate was 72% (n=351). In those cases where ICSI was performed 39.6 hours or more (n=47) after trigger, the fertilization rate was 49% (n=23).

**Conclusion:**The fertilization rate was significantly higher when ICSI was performed 37.5-38.5h post triggering. Considering the results and thinking about obtaining the best fertilization rates, it is suggested that ICSI should be performed up to a maximum of 38.5 hours post trigger, since after this period the fertilization rate reduces significantly.

**Table 1:** Comparison of results in each time of ICSI after the trigger

	<37.5h	37.6h -38.5h	38.6h -39.5h	>39.6h	Total
**Cycles (n)**	29	280	67	9	385
**Oocytes injected**	131	1436	485	47	2099
**Oocytes fertilized**	103	1220	351	23	1697
**Fertilization** **Rate**	79%	85%	72%	49%	81%

## P-27. Oocyte retrieval rate using different protocols to block the LH surge

**Kahisa Natiele Fontana Dal Toso^1^, Regis Yukio Cho^1^, Débora Scaraboto^1^, Isadora Terumi Saruhashi^1^, Viviane Margareth Scantamburlo Niehues^1^, Franciane Poletto Obara^1^, Ana Carolina Possebom^1^, Lidio Jair Ribas Centa^1^**

*^1^Androlab*

**Objective:** To verify the oocyte retrieval rate with desogestrel versus antagonist protocol for preventing a premature luteinizing hormone (LH) surge in a cycle of ovarian stimulation protocol for *in vitro* fertilization (IVF) or oocyte cryopreservation.

**Methods:** Retrospective analysis regarding patients that underwent controlled ovarian stimulation in a private assisted reproduction clinic, between January 2021 and June 2022. Inclusion criteria was use of antagonist or desogestrel protocols. The primary outcome was number of follicles aspirated and number of oocytes retrieved.

**Results:** 260 patients used Antagonist to block the LH surge in the controlled ovarian stimulation with 3623 follicles aspirated and 2770 oocytes retrieved, which leads a oocyte retrieval rate of 76.5% (Table 1). Patients who used Desogestrel instead the rate was 76,7% with 1769 aspirated follicles and 1356 oocytes found in the follicular fluid.

**Conclusion:** LH suppression in IVF or oocyte cryopreservation cycles is usually achieved by using gonadotropin-releasing hormone (GnRH) analogues. The good tolerability and low cost of desogestrel seems to be a good option to be considered when the oocytes or embryos will be all cryopreserved, without fresh embryo transfer. This study showed that there is no difference in oocyte retrieval rate.

**Table 1:** Comparison of result when LH surge block was with Antagonist versus Desogestrel

	Antagonist	Desogestrel
Aspirated Follicles	3623	1769
Retrieved Oocytes	2770	1356
Oocyte Retrieval Rate	76.5%	76.7%

## P-28. Seminal alterations in sedentary patients versus patients practicing physical exercise

**Débora Scaraboto^1^, Isadora Terumi Saruhashi^1^, Andressa Thais Culpi^1^, Kahisa Natiele Fontana Dal Toso^1^, Lidio Jair Ribas Centa^1^**

*^1^Androlab*

**Objective:** To verify the seminal parameters of patients.

**Methods:**1552 patients were included in this study. Data collection about physical exercise practice was carried out through a questionnaire applied to patients who went to a private laboratory looking for a sperm analysis from 2017 to 2022. All semen samples were evaluated between 30-60 minutes after collection. Data about semen analysis have been performed by an andrology analyst. Ideal assessment values were considered: semen volume at least 1.4 mL; sperm concentration of at least 16 million sperm per ml; sperm progressive motility of at least 30% and normal forms of at least 4%.

**Results:** From all 1552 patients, 552 reported being sedentary and 1000 patients reported to practice sports and/or other physical exercises at least once a week. Of the samples from sedentary patients, 51% (n=280) had teratozoospermia, 24% (n=131) had oligozoospermia, and 34% (n=190) asthenozoospermia. Among the patients who practiced physical exercises, teratozoospermia was present in 48% (n=482), oligozoospermia in 21% (n=209), and asthenozoospermia in 30% (n=301). Confidence intervals=95%.

**Conclusion:** As much as the difference is not statistical, progressive motility tends to improve in exercise practitioners in this population, as other scientific articles have already suggested. The practice of physical activities seems to benefit seminal quality and should be encouraged to all patients undergoing infertility treatment.

**Table:** 

	Sedentary subjects	Exerciser subjects	*p* value
**Volume ≤ 1.4ml**	40	65	0.58
**Sperm concentration ≤16 mi/ml**	131	209	0.2
**Progressive motility ≤ 30%**	190	301	0.08
**Sperm morphology ≤ 4%**	280	482	0.34

## P-29. Clinical outcomes of social oocyte cryopreservation. A local experience

**Isadora Seganfredo^1,2^, Mariana Fujji^1^, Alecsandra Prado Gomes^1^, Tatiana Carvalho de Souza Bonetti^1,3^, Pedro Augusto Araújo Monteleone^1,2^**

*^1^Centro de Reprodução Humana Monteleone*

*^2^Disciplina de Ginecologia - Departamento de Obstetrícia e Ginecologia. Faculdade de Medicina da Universidade de São Paulo*

*^3^Departamento de Ginecologia. Escola Paulista de Medicina da Universidade Federal de São Paulo*

**Objective:** In the last decade, the demand for fertility preservation through oocyte cryopreservation has increased concomitantly with the postponement of motherhood. Since 2012, oocyte freezing is no longer considered an experimental technique and vitrification has promoted an important increase in success rates, becoming similar to treatments with fresh gametes. The aim of this study was to evaluate the laboratory and clinical outcomes of elective social egg freezing in a private assisted reproduction center.

**Methods:** This is a retrospective cohort study carried out in a private Assisted Reproduction Center in São Paulo, Brazil. Three hundred seventy three (373) women between 2014 and June 2021 performed elective social egg freezing. Of these, 23 women (6.2%) who had vitrified oocytes for at least one year, performed an ICSI cycles (n=25 cycles) with autologous thaw oocytes between 2019 and 2022. For oocyte collection, the women underwent controlled ovarian stimulation and all mature oocytes were vitrified following standard procedures. In oocyte thaw cycles, surviving oocytes were fertilized by ICSI, embryos were routinely cultured in a time-lapse incubator and transferred to the uterus after a hormone replacement cycle. The results of cycles performed between 2020 and 2022, in women undergoing the first or second IVF cycle, with autologous fresh oocytes injected by ICSI with fresh or frozen-thawed embryos transfers were included as a control group (Control group, n=512), and compared to autologous oocytes thaw cycles (Study group, n=25).

**Results:** The women’s age was 37.9±3.8 for the CT and 36.9±3.6 for the Study group at the time of oocyte collection/vitrification (*p*=0.193). In the Study group, the period between oocyte vitrification and warming was 43.4±20.4 months and a mean of 10.3±5.3 oocytes were warmed per cycle, ranging from four to 30 thawed oocytes. The women’s age at the oocyte warming and ICSI in the study group was 40.5±3.9 years old. The oocyte survival rate after vitrification- warming was 73%. The number of MII oocytes injected was similar between CT and Study groups (7.0±3.0 versus 8.5±6.1; *p*=0.248), but the fertilization (72.7% versus 79.9%; *p*=0.060), blastocyst development (49.4% versus 60.6%; p=0.100) and expanded blastocyst development rate (40.1% versus 56.3%; *p*=0.012) were slightly higher in the Ct than study group, respectively. On the other hand, embryo transfer was performed in most of couples (94% and 87%; *p*=0.280) and the occurred majority at the blastocyst stage (96% and 91%, *p*=0.237). The average number of embryos transferred was 1.3±0.5 for both with similar ongoing pregnancy rates (26% versus 32%; *p*=0.509) in Ct and Study groups, respectively.

**Conclusion:** Despite the few thaw oocytes cycles included in this study, our findings bring a number of important assumptions. The percentage of women who return to use their oocytes after a social oocyte freezing is very small after a mean of 4 year of cryopreservation. Still, laboratory outcomes with thaw oocytes are slightly impaired when compared to fresh oocytes, but resulting in similar clinical success rates. These data are of paramount importance for the social egg freezing clinical planning and warming procedures, considering a loss of about 30% in the vitrification and warming processes (survival rate ≅ 70%) and little lower blastocyst development rate. On the other hand, the very small number of thaw oocytes included limits the generality of our outcomes. In summary, mature oocytes that survived the vitrification- warming process seem to maintain enough potential for fertilization, blastocyst development and implantation compared to fresh eggs, which allows for similar success rates. This study reinforces the oocyte vitrification effectiveness for social egg freezing.

## P-30. Can oocyte morphology interfere on blastocyst formation at day 5?

**Renata Ternus Pedó^1^, Mariana Saikoski Faller^1^, Lauren Dorneles Rebello^1^, Marco Antônio de Bastiani^2^, Carlos Alberto Link^1^, Lucia von Mengden Meirelles^2^, Fabio Klamt^2^**

*^1^Clínica ProSer*

*^2^UFRGS*

**Objective:** The search for non-invasive analysis methods in assisted reproduction techniques has been increasing in order to select the most competent embryos with minimal impact on embryo manipulation and culture. Given that oocyte quality and possibly morphology has a direct impact on fertilization and embryo development, the aim of this study is to correlate morphological alterations of the oocytes with blastocyst formation on day 5.

**Methods:** A total of 883 oocytes from 114 retrievals were analyzed morphologically and classified according to the alterations presented, such as alterations of zona pellucida (ZP), thick ZP, thin ZP, oval ZP, double polar body (PB), large PB, fragmented PB, increased perivitelline space (PS), debris in PS, total granulation of cytoplasm, central granulation of cytoplasm, lateral granulation of cytoplasm, presence of smooth endoplasmic reticulum aggregate, presence of one small vacuole in cytoplasm, presence of one big vacuole in cytoplasm, presence of many vacuoles in cytoplasm, presence of inclusions in cytoplasm, dark cytoplasm, altered cytoplasm, irregular form and presence of a dark spot in cytoplasm. The oocytes undergoing intracytoplasmic sperm injection (ICSI) were evaluated and cultivated separately until blastocyst stage, when they were destined according to each patient’s treatment. Descriptive statistics were compiled for the morphological variables and days. We evaluated the associations between morphological variables and days using Fisher’s exact test (FET) for count data. All analyses were implemented using the R statistical environment.

**Results:** At the time of the ICSI, 850 oocytes were morphologically evaluated according to the selected alterations. Of the oocyte morphological alterations evaluated, only two showed significant statistical difference (*p*<0.05) when correlated with blastocyst formation, and one presented a borderline relation, being them: alterations of ZP (n=60), dark spot in cytoplasm (n=6) and the presence of fragmented PB (n=82). Of the oocytes that presented ZP alterations (7.06%), 51 (80.95%) fertilized and 6 (13.33%) reached the blastocyst stage; Of the oocytes that presented a dark spot in cytoplasm (0.71%), 6 (100%) fertilized and 5 (83.22%) reached the blastocyst stage; Of the ones that presented fragmented PB (9.65%), 73 (89.02%) fertilized and 26 (31.70%) reached the blastocyst stage. A total of 631 embryos from 850 oocytes initially evaluated were accompanied until day 5 of embryo culture and 203 reached the blastocyst stage. The presence of altered ZP was higher in the group of embryos that didn’t reach the blastocyst stage (8.87%) when compared with the blastocyst group (2.95%), showing a significant statistical difference (*p*=0.006). The presence of a dark spot in cytoplasm was higher in the group of embryos that reached the blastocyst stage (2.46%) when compared with the non- blastocyst group (0,23%), showing a significant statistical difference (*p*=0.003). The fragmented PB was more present in the blastocyst group (12.80%) when compared with the group of embryos that did not reach the blastocyst stage (7.94%), but this correlation did not reach statistical significance (*p*=0.059).

**Conclusion:** The presented results show a possibility that oocytes with altered ZP have a negative relation on blastocyst formation, while the oocytes with the presence of a dark spot in cytoplasm show a positive relation with blastocyst formation at day 5. The oocytes that presented a fragmented PB had a higher correlation with blastocyst formation, although this result was not statistically significant. The rest of the morphological alterations evaluated demonstrated to not interfere on blastocyst formation at day 5.

## P-31. Efficacy of frozen-thawed elective single embryo transfer (ESET) compared to double-embryo transfer (DET) without PGT-A in a fellowship program

**Cindy White Loureiro Souza^1^, Paulo Homem de Mello Bianchi^1^, Camila Sommerauer Franchim^1^, Henrique Penna Fazao^1^, Giovanna Santos Cavalcanti^1^, Vanessa Heinrich-Oliveira^1^, Carlos Augusto Zarate Nissel^1^, Pedro Felipe Magalhães Peregrino^1^, Pedro Augusto Araújo Monteleone^1^, Edmund Chada Baracat^1^**

*^1^Hospital das Clínicas da Faculdade de Medicina da Universidade de São Paulo (HCFMUSP)*

**Objective:** To evaluate the performance of eSET compared to DET without embryo selection by PGT-A in a fellowship program.

**Methods:** This retrospective study analyzed data of 328 frozen-thawed embryo transfers at blastocyst stage (day 5 or 6) from 216 couples, totalizing 565 non-PGT-A embryos transferred from January 2019 to December 2021. A maximum of two IVF cycles with flexible antagonist protocol including 150-225 IU/day of HMG or FSH-r; cryopreservation of all viable embryos followed by up to four embryo transfers in medically prepared endometrium (oral estradiol valerate 6mg/day and vaginal natural micronized progesterone 600mg/day) were performed per couple. Patients were selected for eSET if they met the following criteria: good ovarian reserve (antral follicle count ≥10); non obese; without severe endometriosis and severe male factor. Pregnancy rate (serum beta human chorionic gonadotrophin -bHCG- ≥15mUI/ml 9 days after embryo transfer) and clinical pregnancy rates (transvaginal ultrasound detection of an embryo with heartbeat between gestational weeks 6 and 7) were compared between groups according to females’ age (≤35 or >35 years old) by the time of fertilization: group 1 (eSET ≤35 y/o; n=46) versus group 2 (DET≤ 35 y/o; n=73); group 3 (eSET>35 y/o; n=42) versus group 4 (DET >35 y/o; n=55). Implantation rate was calculated by dividing the total number of gestational sacs per total number of embryos transferred.

**Results:** The mean age of patients in each group was: group 1 (31.5 y/o ± 2.7 SD), group 2 (31.4 ± 2.9), group 3 (38.3 ± 1.7) and group 4 (38.9±2.2). There was no statistically significant difference in mean patient´s age between groups 1 and 2 (*p*=.85), and between groups 3 and 4 (*p*=.14). No statistically significant difference in cumulative pregnancy rates nor cumulative clinical pregnancy rates per patient were observed when comparing groups 1 and 2 (60.9% x 65.8%, *p*=.58; 39.1% x 46.6%, *p*=.42, respectively) as well as comparing groups 3 and 4 (52.4% x 54.5%, *p*=.83; 31% x 38.2%, *p*=.46, respectively). Also, there was no significant difference in implantation rates in the eSET (36%) group versus the DET (47%) group (*p*=.14). Regarding multiple gestations, 7 were identified, all resulting from DET (6 in group 2 and 1 in group 4).

**Conclusion:** In a good prognosis group of patients in our fellowship program with in- training physicians, eSET was associated with similar cumulative pregnancy rates, clinical pregnancy rates and implantation rates when compared to DET, even without embryo selection by PGT-A. Besides, in the eSET group there were no multiple pregnancies. Even in settings where physicians are being trained, eSET could be considered for good prognosis patients, regardless of availability of embryo selection by PGT-A. Further studies in larger data sets may help identify if selected patients might benefit from DET.

## P-32. Aspects of insulin metabolism in patients undergoing IVF: Literature review

**Lígia Previato^1^, Leonardo Previato de Araújo^1^, Cássio Leão Fácio^1^, Ligiane Alves Machado-Paula^1^, Edilberto de Araújo Filho^1^**

*^1^Centro de Reprodução Humana de São José do Rio Preto (CRH Rio Preto), São José do Rio Preto, SP, Brasil*

**Objective:** Integrative literature review with the objective of evaluating whether hyperinsulinemia interferes with the ovary and, consequently, the ovulation process, oocyte quality and pregnancy rates in patients undergoing IVF, since insulin is an important modulator of follicular development, steroidogenesis, maturation oocyte and embryonic development.

**Methods**: Literature search was conducted in Pubmed and SciELO databases, using keywords: insulin; hyperinsulinemia; fertility; infertility; polycystic ovary; metabolic syndrome; Assisted Human Reproduction; *In vitro* fertilization (IVF); ovary; oocyte quality and obesity. Studies in humans undergoing IVF treatment were selected. Inclusion criteria: articles published in the last 10 years, original research, cross-sectional studies carried out in human population, with more than 30 patients and systematic reviews.

**Results:** Of 237 studies reviewed, 39 were included in this review, being 30 retrospective and 9 prospective. The results obtained in this literature review showed that due to the high prevalence of obesity, especifically in women of reproductive age, there is a concern related to the risks to these women’s fertility, due to the increased risk of type 2 diabetes, metabolic syndrome (MetS) and other noncommunicable diseases. The MetS is composed of hypertension, dyslipidemia, abdominal obesity and insulin resistance or glucose intolerance and has an adverse impact on female reproduction due to impaired endometrial receptivity and compromised embryonic development. Obesity is also associated with polycystic ovarian syndrome (PCOS), and many of the metabolic abnormalities of MetS also overlap with PCOS. About 50% to 70% of women with PCOS have insulin resistence and infertile women with PCOS had higher levels of fasting insulin, and the resultant hyperinsulinemia plays a role in the pathogenesis of reproductive disorders. Therefore, knowing that insulin has an action on both ovulation and oocyte quality, hyperinsulinemia implies impaired fertility, i.e., altering the ovulatory process, producing low-quality oocytes, probably due to increased inflammation and oxidation, in addition to disrupts the intrafollicular microenvironment during folliculogenesis and reduces the rate of fertilization and embryonic development potential during the natural and ovarian stimulation cycles. Obese women, when undergoing assisted reproduction techniques (ART), require higher doses of gonadotropins (Gn); have an altered follicular environment, poor or unpredictable ovarian response to hormonal stimulation; more cycles canceled; few granulosa cells and cumulus-oocyte complexes in egg aspiration; more immature oocytes; fewer oocytes retrieved; abnormal morphology and spindle malformation; lower fertilization rates; incomplete or impaired embryonic development after ICSI/IVF; lower implantation and pregnancy rates and higher miscarriage rate; lower overall live birth rate after IVF compared to normal weight women. If they become pregnant, they have more gestational complications and risk of morbidity, spontaneous preterm birth, reduced rates of live births and greater chance of stillbirth, increased risk of complications during delivery, lower birth weight and health complications for babies.

**Conclusion:** No studies have addressed how women are being counseled and understood about the effects of obesity on reproductive outcomes. An altered metabolic environment negatively affects reproductive outcomes before conception even occurs, therefore it is essential that intervention occurs in the pre-conception period. Thus, it is very important that they are guided about changes in habits, to control insulin levels, through a healthy diet, prioritizing foods rich in fiber and antioxidants, natural and whole, reducing or excluding ultra-processed foods, reduction of saturated fat, trans fat, in order to maintain adequate insulin and blood glucose levels. It is part of this process to include the practice of physical exercises in the daily routine, and weight control for those who are overweight. This can increase fertility and the chances of pregnancy in patients undergoing IVF.

## P-33. Case report: Successful Treatment of a Cervical Heterotopic Gestation after *in vitro* Fertilization with Ultrasound-guided Aspiration, Cervical Curettage and Pessary Use

**Cintia Morel Correa^1^, Sergio Galbinski^2^, FF Bassol^2^, Nilo Frantz^3^, Adriana Bos-Mikich^4^**

*^1^Progest Asssited Reproduction*

*^2^Unidade de Reprodução Humana, Hospital Femina*

*^3^Nilo Frantz Reprodução Assistida*

*^4^Universidade Federal do Rio Grande do Sul.*

**Objective:** The lack of standard treatment guidelines for the management of a cervical heterotopic gestation (CHG) poses a challenging clinical situation, particularly after IVF. Thus, it is foremost to detail situations and treatment procedures that reached a fortunate ending in terms of fertility preservation, gestation and delivery, more specifically after Assisted Reproduction (AR) treatment.

**Methods**: JAR, 41years old nulliparous, with diminished ovarian reserve and two previous IVF attempts with autologous oocytes presented at our AR service. Her AMH was 0.7 ng\ml and both treatment cycles were unsuccessful. In her first IVF attempt using donor oocytes, three blastocysts were created and transferred in two consecutive cycles, but did not result in gestation. Another donor-oocyte cycle followed, and she received two grade 1 fresh blastocysts resulting in β-HCG of 355mUI. Ultrasound 26 days post-transfer showed one intrauterine gestational sac and another in the cervix, with both embryos measuring 0.5cm and with active heartbeat. The patient received extensive information and a US-guided aspiration of the cervical gestational sac was decided, to best preserve the intrauterine gestation. The procedure was performed under general anesthesia, on day 28 after embryo transfer. The cervical gestational sac was punctured using a 17-G single lumen oocyte aspiration needle under US-guidance. The treatment approach was successful in aspirating the contents of gestational sac. The patient was discharged home after 24 hrs observation. The histopathological examination confirmed embryonic remains and products of conception from the aspirate. There was minimal bleeding during and after the procedure. On the second post-operative day, the patient presented with painless vaginal bleeding and was readmitted to the hospital. Trophoblastic tissues of the cervical ectopic conceptus were gently removed using a curette, under ultrasound guidance. Histopathology analysis confirmed the presence of chorionic villi in the gestational tissues. An ultrasound performed four days after the curettage revealed a viable intrauterine gestation. Serial obstetric US six days later revealed the persistence of a heterogeneous, hypervascular mass measuring 2,7 x 2,0 mm in the cervical canal near the internal *os*, corresponding to remains of the cervical gestation chorionic villi. Two weeks after the curettage, the cervical heterogeneous mass persisted and the intrauterine gestation followed normal development. Fortnightly US surveillance revealed a shortened uterine cervix, at 20 weeks gestation. A cervical pessary was inserted at 22 weeks gestation to avoid preterm delivery. The patient was prescribed absolute bed rest until delivery.

**Results:** Labor contractions started at 37 weeks gestation. The pessary was removed and the cesarean section was schedule for the next day. The patient delivered a healthy baby girl weighting 2960g and 46cm.

**Conclusions:** Considering that there is no consensus on the best treatment for CHG, it is important to report the factors that contributed to the successful resolution of the present case. Early detection and US-guided aspiration performed by an experienced operator, careful follow up of the remaining embryonic tissues, monitoring of cervical canal along the gestation to avoid preterm delivery due to a possible shortening of the cervix after curettage and placement of cervical pessary to ensure a full term gestation represent a safe and effective management of CHG, with fertility preservation and favorable live birth outcomes for IVF patients.

## P-34. Effect of the anti-inflammatory diet in the control of oxidative stress and its relation with quality of life and gestational success in patients with endometriosis

**Milena Rojas Antunes^1^, Isabela Clarassoti Simionato^1^, Gustavo Barbosa^1^, Gabriel Monteiro Pinheiro^1^**

*^1^UNISA*

**Objective:** This literature review aims to evaluate the impact of an anti-inflammatory diet in the control of oxidative stress and its relationship to quality of life and gestational success in patients with endometriosis.

**Methods:** Literature review based on 7 articles collected in Pubmed, BVS and SciELO databases, published between 2013 and 2021 in Portuguese and English.

**Results:** Endometriosis is a multifactorial chronic inflammatory disease characterized by the extrauterine growth of endometrial glands capable of generating infertility, dyspareunia, dysmenorrhea, pain, intestinal bleeding, mainly affecting women of childbearing age. It is believed that there is a strong relationship between psychological-environmental factors and an unbalanced diet with the development of endometriosis. These aspects influence body imbalance, generating disparity between pro-oxidant agents (free radicals) and the body’s antioxidant defense mechanisms, promoting a systemic overload and, consequently, oxidative stress.

Nutrient-deficient diets cause changes in lipid metabolism, oxidative stress and contribute to epigenetic abnormalities that may be involved in disease genesis and progression. Therefore, oxidative stress damages mesothelial cells present in the pleural, peritoneal and pericardial cavities, and in the tunica albuginea of the ovary, leading to adhesion of endometrial cells. Therefore, it contributes to the development of endometriosis, in addition to resulting in excessive production of reactive oxygen species, influencing women’s folliculogenesis, oogenesis and fertility.

On the other hand, it was observed that Omega-3, N-acetylcysteine, vitamin D and resveratrol, in addition to a natural and healthy diet, exert a protective effect, with a reduction in the risk of development and possible regression of the disease. The antioxidant vitamins C and E are involved in scavenging free radicals and reactive oxygen species. In addition, B vitamins, particularly pyridoxine, increase the metabolism of estrogen in an inactive form and aid in the conversion of linoleic acid to gamma-linolenic acid, an essential component of anti-inflammatories production, which may inhibit the growth of endometrial tissue.

As a result, it is suggested that endometriosis may, among other causes, be not only associated with oxidative stress, but also have its etiopathogenesis justified by it.

An antioxidant diet with vitamin C, vitamin E, vitamin D and omega-3 have shown good results in decreasing pelvic pain in addition to reducing inflammatory markers. Foods rich in thiamine, folate, vitamin C and vitamin E obtained exclusively from dietary sources may decrease the rate of laparoscopically confirmed endometriosis, but it is not known whether these nutrients themselves, or an unidentified dietary pattern abundant in these nutrients, underlie the associations. observed. In contrast, high consumption of red meat has been shown to increase levels of estradiol and, consequently, inflammation, thus leading to a greater risk of developing endometriosis.

**Conclusion:** This review concluded that there is evidence that food and nutrients influence both the pathophysiology and progression of the disease, leading to the possibility of alternative and adjuvant treatments for those suffering from the disease. Thus, we highlight the role that nutrition plays in the lives of these women in order to prevent, alleviate and/or improve the situation in which they find themselves. Therefore, with a nutrient-deficient diet, the imbalance can contribute to the onset or worsening of diseases other than endometriosis, such as: recurrent miscarriages, premature menopause, unexplained infertility and much more. All things considered, dietary reeducation seems to be an alternative in the prevention and treatment of endometriosis.

## P-35. Is mosaic embryo transfer a possibility? Results and findings from our assisted reproduction clinic in São Paulo, Brazil

**Augusto Azzolini de Melo^1^, Vinicius Genuino^1^, Nicolle Barbosa Katayama^1^, Rafaela Ferrarezi Maluf^1^, Ian Sakiyama de Almeida^1^, Anny Caroline Gomes de Almeida^1^, Bruna Ferreira Rech^1^, Rebeca Santos de Oliveira^1^, Vanessa Rodrigues Alves^1^, Phlip Wolff^1^**

*^1^Clinica Genics.*

**Objective**: To evaluate the possibility of embryo transfer with mosaic result through the preimplantation genetic analysis (PGT-A) report.

**Methods**: 5 to 10 trofectoderm cells from blastocyst stage embryos were biopsied and sent to the genetic analysis laboratory for determination of euploidy of the embryos by next-generation sequencing (NGS) by the preimplantation genetic test for aneuploidy (PGT-A). Statistical analyses of mean and standard deviation were performed for the total age of patients.

**Results**: A total of 1402 Assisted Reproduction cycles totaling 4354 embryos in blastocyst stage from January 2020 to December 2021 from patients aged 37 years on average and standard deviation of ± 3.5 years were analyzed. The results of PGT-A analyses were 2034 (46.7%) normal, 2224 (51.8%) altered, 23 (0.53%) DNA amplification failure and 73 (1.68%) had mosaic alteration. Of the 1402 cycles, 62 (4.42%) obtained mosaic embryos, and of these we obtained 15 cycles with 1 euploid embryo, 31 cycles with 2 or more euploids. Following this, 16 of these cycles did not obtain any euploid embryos. None of the 16 cycles had mosaic embryos with chromosomes considered critical such as 13, 18, 21, X and Y. Taking into consideration low grade mosaicism, only 11 (0.78%) of the cycles obtained this result. To date, only two low-grade mosaic embryo transfers have been performed, and both did not lead to clinical gestation.

**Conclusion**: Our results showed that mosaic embryo rates are low per human reproduction cycle and the possibility of transfer is rare. According to the current literature, the clinical and laboratory findings on mosaic embryo transfer may be interesting for patients who do not have euploid embryos as it may provide a chance for these patients, however the small number of patients who opted for mosaic embryo transfer indicates to us that the possibility of reaching the desired pregnancy is reduced.

## P-36. Evaluation morphocinetic in euploidy embryos, does it matter?

**Ricardo Azambuja^1^, Fabiana Wingert^1^, Shana Flach^1^, Marta Hentscke^1^, Mariagela Badalotti^1^, Alvaro Petracco^1^**

*^1^FERTILITAT*

**Objective**: To evaluate when only euploid embryos were transferred if the morphocinetics observed in these embryos have an effect in the pregnancy rate. The morphocinetcs pattern was performed by KIDscoreTM D5^®^.

**Methods**: This was a retrospective study analyzing the KIDscore of 194 embryos that were submitted to genetics test (PGTA) during the period of 2020 to 2022, and the report came all as euploid embryos. The embryos were divided in three groups according the KIDscore A (1-4), B (4.1-7), C (7.1 - 9,9). Fifty-three embryos were in group A, 91 in group B, and 50 in group C. All 194 euploid embryos were transferred following endometrial preparation, and transfer guided by ultrasound. All embryos were cultured in a time-lapse Embryoscope^®^ (Vitrolife, Canadá) incubator, and all embryos were scored using the Kidscore algorithm prior to genetics analysis. Statistical analysis were performed using qui- square and ANOVA, with p<0.05.

**Results:** The pregnancy rate found for each group were: group A (25/53, 47.2%), group B (47/91; 51.6%), and group C (33/50; 66.%), no statistical difference was observed (*p*=0.13).

**Conclusion**: The results suggest that the highest kidscore group had the highest pregnancy rate. Maybe by increasing the number of embryos in each group, a statistical difference may be found in the future. In this report there is a trend that even for the euploid embryos, the morphocinetic evaluation is important.

## P-37. How reliable is PGT-A? A case report of pregnancy with triploid fetus after normal embryo biopsy

**Alvaro Petracco^1^, Mariangela Badalotti^1^, Isadora Badalotti-Teloken^1^, Catarina Heckmann Petracco^1^, Marta Hentschke^1^, Ricardo Azambuja^1^, Maria Teresa Sanseverino^1^**

*^1^FERTILITAT*

**Objective:** The risk of developing embryos with chromosomal abnormalities increases with maternal age due to oocyte aging. Preimplantation genetic testing for aneuploidies (PGT-A) identifies numerical chromosomal alterations, improving the chance of a healthy baby and shortening the time to pregnancy.

**Methods:** Case Report

**Result:** Couple, both 43 years old, healthy, with a history of pregnancy at 22 years old with spontaneous abortion (12 weeks) and a 1-year *in vitro* fertilization (IVF) cycle without pregnancy (7 eggs, no embryo to transfer). A new IVF cycle with PGT-A was performed. After ovarian stimulation, four mature eggs were retrieved and inseminated by intracytoplasmic sperm injection; 18h after insemination, the four oocytes exhibited two pronucleus (normal fertilization); three reached the blastocyst stage at D+5 and were biopsied and analyzed by New Generation Sequencing (NGS). The analysis showed one euploid embryo (46, XX), one complex aneuploid embryo (+8,+18, +21), and one chaotic aneuploid embryo. One year later, the euploid embryo was transferred, which resulted in clinical pregnancy. Ultrasound at 12 weeks showed reduced fetal movement, holoprosencephaly, inconclusive nasal bone, and ductus venosus pulsatility above the 95th percentile. A non-invasive prenatal genetic test was performed, which showed normality for the 24 chromosomes and the absence of the most common microdeletions. Amniocentesis was performed at 15 weeks of pregnancy, and the fetal karyotype showed triploidy (69, XXX). The pregnancy was interrupted 16 weeks.

**Conclusion:** the PGT-A is an important tool to increase the chances of giving birth to a healthy baby. Although the PGT-A is around 98% accurate, there is a 1-2% risk of a false positive or false negative concerning numerical aneuploidies. Among the limits is the possibility that the test is not informative due to the absence or poor quality of the DNA of the collected cells. Other limitations include the impossibility of identifying some alterations, including polyploidies, as in the case described, parental disomy, balanced structural anomalies, or structural anomalies smaller than the platform can identify. Chromosomal copy number analysis by NGS does not require a large amount of data as it is determined using Shallow Whole Genome Sequencing (sWGS). This data can be obtained within 24 hours, and sWGS by NGS allows the simultaneous analysis of a large number of samples, thus reducing the cost per sample. On the other hand, this method is limited in detecting polyploidy, a common genetic cause of miscarriage. Whereas triploidy containing a Y chromosome can be detected by calculating the sex chromosome ratio, polyploidy without a Y chromosome, such as 69.XXX and 92.XXXX is very difficult to distinguish from 46.XX using NGS-based sWGS. Several studies have tried to overcome limitations with the use of additional techniques for the detection of triploidy, such as the QF-PCR analysis, which allows identifying the parental origin of the chromosomes. In all situations of error or impossibility of diagnosis, an embryo with a genetic abnormality will be transferred. Then, it is crucial to inform the patient about the risks and limits of the exam.

## P-38. Is there an influence of the embryonic development day with the DNA amplification failure and embryonic mosaicism?

**Augusto Azzolini de Melo^1^, Vinicius Genuino^1^, Nicolle Barbosa Katayama^1^, Rafaela Ferrarezi Maluf^1^, Ian Sakiyama de Almeida^1^, Anny Caroline Gomes de Almeida^1^, Bruna Ferreira Rech^1^, Rebeca Santos de Oliveira^1^, Vanessa Rodrigues Alves1, Phlip Wolff^1^**

*^1^Clinica Genics.*

**Objective:** Assess the influence of the embryonic development day, day 5 and day 6 (D5 and D6) with the rates of mosaicism and DNA amplification failure through the preimplantation genetic test for aneuploidies (PGT-A).

**Methods:** 5 to 10 cells of the trophectoderm of embryos at the blastocyst stage have been biopsied, sent to the genetic analysis laboratory for the euploidy determination through the next generation sequencing (NGS) via the preimplantation genetic screening for aneuploidies (PGT-A). The statistical analysis of mean and standard deviation for the total patient’s ages. A chi-square test was used to analyze the proportions of results of mosaicism and DNA amplification failure on PGT-A in D5 and D6.

**Results:** 1402 cycles of human reproduction have been analyzed, with the total of 4267 embryos at the stage of blastocysts from January/2020 to December/2021 with patients that were, in average, 37 years old and standard deviation of ± 3,5 years. The results of the PGT-A analysis were of 2018 (47,29%) normal, 2156 (50,53) altered, 23 (0,53%) DNA amplification failure and 70 (1,64%) had mosaic chromosomal alterations. Of these, 2662 were biopsied on D5 and 1605 on D6, in which the DNA amplification failure were of (14; 0.53%) and (9; 0.56%) on D5 and D6 respectively the Chi-square *p*-value=(0.880). The mosaicism rate was (45;1.69%) and (25;1.56%) on D5 and D6 respectively the Chi-squar *p*-value=(0.740).

**Conclusion:** The incidence of mosaicism and DNA amplification failure is not related on the day which the biopsy has been done, D5 or D6.

## P-39. Patient care in the preparation and use of ovulation inducing medications

**Caroline Arenales Venna^1^, Giovanna Bonatto Luca^1^, Victoria Castardo Navas Bernal^1^, Gabriel Monteiro Pinheiro^1^**

*^1^Universidade Santo Amaro*

**Objective:**The objective of the present study is to analyse the patient’s care to get prepared and use of ovulation-inducing medications.

Methods:It is a narrative review of 13 articles published in English and Portuguese on (PubMed, Scielo, LILACS) platforms between 2000 and 2020.

Results: To choose a ovulation protocol we must first identify the etiology of the patient’s anovulation, and after an individualized analysis, make lifestyle modifications or treat underlying medical conditions that negatively influence the treatment. In addition, checking the conditions of the woman’s reproductive system through ultrasound, as well as evaluating the ovarian reserve is something primordial, to improve the chances of artificial reproductive treatments (ART).

The ovulation protocol is mainly based on the using drugs that promote an increase in FSH supply to the ovaries, the most commonly used are clomiphene citrate, Aromatase inhibitors (AIs), metformin and gonadotropins.

***Clomiphene citrate (CC)*** is considered a first-line treatment drug in unexplained infertility couples, which stimulates endogenous FSH,LH, and GnRH agonists. It is usually used for 5 consecutive days, matching with the beginning of the menstrual cycle, for 3 to 6 monthly cycles. Although ovulation occurs in 70-80% of women, in only 30-50% of cases culminate in pregnancy.

***Aromatase inhibtors (AIs)*** is considered a first-line treatment drug in polycystic ovary syndrome (PCOS) patients with almost the same function as CC, with less endometrial aggression and producing unifollicular cycles, with a higher Life birth rate (LBR) with less multiple pregnancies.

***Metformin*** is a drug that can be used alone to restore folliculogenesis, decreasing insulin resistance, but it is commonly used in association with oral medicines for inducing ovulation. Ovulation with significantly increases the rate of ovulation and pregnancy.

***Gonadotropins*** are used to stimulate ovulation related to low levels of natural gonadotropins or estrogen, and are also an option when clomiphene citrate alone or in combination is ineffective, such as in cases of polycystic ovary syndrome (PCOS).As important as the drugs to induce ovulation are their ***blockers, GnRH Antagonists*** stand out. Because from the moment that the aspiration of the follicles belonging to the selected cohort is done, it is extremely important to obtain control of the hormonal stimulation that may turn the environment for a future nidation of an improper oocyte.It is fundamental the use of ultrasonography to analyze if the follicles are developing as expected, and if there is only one dominant follicle at the end of the process aso that the woman releases only one egg, thus avoiding the risk of multiple pregnancies and ovarian hyperstimulation syndrome (OHSS). Another point that deserves attention is the need for psychological support during the treatment, because anxiety, depression and decreased quality of life can have a negative impact. In addition, many infertile women feel abnormal and incomplete, so it is necessary that the psychologist and the physician make the couple aware of the challenges that will be faced to helping them deal with the uncertainties and the next steps in the treatment.

Conclusion: It is understood that the process of ovulation induction involves a series of steps and multidisciplinary care, starting with the identification of the cause of anovulation, analysis of ovarian reserve and conditions of the reproductive system of the couple. Among the range of drugs used, and ultrasound monitoring is required throughout the treatment. Furthermore, the procedures involved in the diagnosis and treatment of infertility may exacerbate psychological problems, thus emphasizing the importance of the psychological approach which must be instituted from the initial stages of clinical follow-up, persisting until the birth of the fetus.

## P-40. Use of oral progestin during controlled ovarian stimulation in a patient with 45,X/46,XX mosaicism: A case report and literature review

**Fernanda Godoy Cabral de Oliveira^1^, Caroline Castrucci Ingold^1^, Thais Gigliotti Malheiros Luzo^1^, Caio Parente Barbosa^1^, Renato de Oliveira^1^, Bianca Bianco^1^**

*^1^Instituto Ideia Fértil - Centro Universitário FMABC*

**Objective:** Description of a case report of a patient with Turner mosaicism, endometriosis and male factor who achieved pregnancy with the use of oral progestin (PO) in controlled ovarian stimulation (COS) and literature review.

**Methods:** Case report and literature review

**Results:** J.A.M. 35 years old, female, white, nulliparous, regular cycles and the partner F.S.N., 40 years old, with 2 children from a previous relationship, vasectomized for 7 years, with primary infertility for 1 year and 2 months. She has symptomatic endometriosis, clinically diagnosed, and a 45,X[3]/46,XX[47 karyotype, with no other comorbidities. She underwent fertility cryopreservation prior to videolaparoscopy for endometriosis. At COS, the antral follicle count was 10 and was used a protocol with recombinant FSH 200 IU/day (Menopur^®^) for 10 days, inhibited ovulation with dydrogesterone 20 mg/day (duphaston^®)^ and trigger with triptorein 3 ml (gonapeptyl daily^®^). After the identification of 6 pre-ovulatory follicles, 6 oocytes were captured in metaphase II (MII). After 6 months of ovarian blockage with dienogest and symptoms improvement, she chose not to undergo surgery and started endometrial preparation with estradiol 6mg/day and luteal support with micronized progesterone 800 mg/day (utrogestam^®^). Her partner has a 46,XY,+1qh+[20] karyotype, a history of unsuccessful vasectomy reversal 2 years ago (azoospermia) and an indication for PESA (Percutaneous Epididymal Sperm Aspiration). Fresh embryo transfers of 2 embryos on the third day (8A and 8A) was performed, without preimplantation genetic test for aneuploidy (PGT-a) by couple’s option, in addition to cryopreservation of the 3 blastocysts (4BC/3BC/5BC). The patient became pregnant and the first morphological ultrasound indicated a single topical pregnancy compatible with normality.

Turner syndrome (TS) is defined as a set of phenotypic characteristics in women in which there is total or partial loss of the second sex chromosome. Most have a total deletion of one of the X chromosomes; 25%, partial deletion of one of them, and 20%, varying degrees of mosaicism. Generally, there is an accelerated decline in ovarian reserve before puberty, and in cases of mosaicism, the loss may be delayed for a variable period after menarche. However, it culminates in premature ovarian failure. Only 2-10% of patients with TS conceive naturally. Of these, most have mosaicism. Only 5,7% achieve a live birth. In this context, early embryo or oocyte cryopreservation is recommended with PGT-a. For this purpose, COS traditionally uses the GnRH antagonist to block the premature LH surge.Recently, CUI et al., 2021 carried out a systematic review on the use of PO to block ovulation, demonstrating live birth rates, duration of COS and number of MII similar to traditional blocks, being more comfortable by oral administration and least expensive. The peculiarities of this case could limit the COS to traditional protocols. However, this case report aims to contribute to the process of legitimizing a strategy for the use of PO in COS in order to minimize costs and increase comfort by switching from an injectable to an oral medication, even in unusual situations.^(4)^

**Conclusion:** As far as we know, this is the first case report of the use of PO in COS in a patient with Turner mosaicism. Despite the difficulty of pregnancy with own oocytes in patients with this condition and, in addition, the presence of endometriosis and male factor, pregnancy was successful. Thus, we emphasize the feasibility of using PO in COS in situations little exploited, allowing cost reduction and increasing the comfort of the administration route, within a proposal of greater individualization and humanization of treatment.

## P-41. Influence of infertility factor associated or not with endometriosis on ovarian stimulation outcomes in patients undergoing *in vitro* fertilization (IVF)

**Camila Dutra de Souza Francisquini^1^, Giulianna Pereira Tizzot^2^, Leonardo Pinho Carreño^2^, Vinicius Bonato da Rosa^3^, Samara Artuso Giacomin^1^, Alessandro Schuffner^1^**

*^1^Conceber Centro de Medicina Reprodutiva*

*^2^Faculdades Pequeno Príncipe*

*^3^Brown Fertility - Florida Fertility Clinics*

**Objective:** The objective of the present study was to evaluate, according to the infertility factor endometriosis associated or not with another infertility female factor and the male factor, the influence on the response to ovarian stimulation, on the oocyte fertilization and on canceled cycles.

**Methods**: In this retrospective study, the results of the ovarian stimulation, oocyte recovery and fertilization in patients submitted to in-vitro fertilization (IVF) between the years 2012 and 2020 were compared. As inclusion criteria, we utilized just patients aged 24 to 45 years, with their own oocytes recovered from ovarian pick-up and submitted to Intracytoplasmic sperm injection (ICSI) and semen from the fresh ejaculate. The couples were divided into 5 groups regarding to the infertility factor associated or not with endometriosis, being: Group 1 - endometriosis without another infertility factor (n=102); Group 2 - endometriosis + other infertility female factor (FF) (anatomical factor, endocrine factor or ovarian failure) (n=117); Group 3 - endometriosis + infertility male factor (MF) (n=30); Group 4 - Infertility female factor (excluding endometriosis) (n=1095); and Group 5 - Infertility female factor (excluding endometriosis) + infertility male factor (n=384). The parameters that were analyzed are: woman´s age, total FSH cumulative dose (International Unit - I.U.), number of follicles >12mm in the triggering day with hCG, number of oocytes recovered, number of mature oocytes, number of oocytes fertilized and number of cycles canceled. The measures were compared by analysis of variance (ANOVA one- way) by Tukey’s test, with *p*-value of 0.05 and the canceled cycles rates (%) were compared between the groups using the Z-test statistical method for 2 proportions, adopting the significant *p*-value of *p*<0.05.

**Results:** By these means, it can be observed that there was no statistical difference between the groups for age and I.U (*p*>0.05), demonstrating the homogeneity of the groups. In the group “FF with absence of endometriosis + MF” there was a greater production of follicles (11.5±7.2; *p*<0.05) when compared to the “FF with no endometriosis (9.1±6.3)” and “endometriosis+FF (8.3±4.8)” groups. The group “FF (absence of endometriosis)+FM” had a higher number of oocytes recovered (10.1±7.22; *p*<0.05) compared to “FF without endometriosis” (7.9±6.3) and “endometriosis+FF” (7.6 ±5.1). Regarding to the number of MII oocytes, the “FF with absence of endometriosis+FM” group had the highest number (7.4±5.8; *p*<0.05), compared to the “FF with no endometriosis” (5.9±4.9), “endometriosis + FF” (5.7±3.7) and “endometriosis” (6.1±4.3). It was observed that the groups with the highest proportion of canceled cycles (p<0.05) were “endometriosis + FF” (3.41%) and “FF without endometriosis” (4.02%), compared to “FF with no endometriosis + FM” (1.30%), “endometriosis” (0.98%) and “endometriosis + FM” (0%). As for fertilized oocytes, there was no statistical difference between the groups (*p*>0.05).

**Conclusion:** Based on the results presented is possible to conclude that couple where the woman has a female infertility factor, whether anatomical, endocrine or ovarian insufficiency, except endometriosis, associated with the male factor, has a greater number of follicles, a greater number of recovered oocytes and a greater number of mature oocytes in relation to couples who have as an infertility factor only the female factor or endometriosis associated with another female factor. That being sad, endometriosis only causes a decrease in these parameters when associated with another female factor. Whereas, in these 2 groups (Female factor and endometriosis + female factor), they were those where the highest rates of cycle cancellation occurred.

## P-42. Comparative analysis on the difference in the amount of blastocyst formed in icsi cycles using Zymot in the second cycle

**Mariana Santos Costa^1^, Edlla Mikaine Padre e Fechine^1^, Fúlvia Estefânia Padre e Fechine^1^, Patricia Tourinho^1^**

*^1^Fertvida*

**Objective**: Research focused on the study of *in vitro* reproduction shows that the way semen is processed can directly impact the results from fertilization, development and blastocyst formation. There are some seminal processing methods that are already widely used in laboratories, including, for example, density gradient and swim-up. Zymot is a new assisted reproductive technology (ART) used for sperm selection. This device features a separation membrane and, based on sperm motility, selects the best ones to be used in ICSI. This technology began to be used at Fertvida - MA in September 2021 and an increase in the number of frozen blastocysts was observed in patients who used the Zymot plate in the second cycle. This study compared the number of embryos that reached a blastocyst between patients who used conventional methods of seminal preparation in the first cycle and the Zymot plate in the second cycle.

**Methods:** Patients from a single center who had male infertility factors or age and underwent two cycles of ICSI between May 2021 and May 2022 were included in this study. During the first cycle, sperm preparation was performed using gradient or swim-up techniques, and in the second cycle, preparation was performed with the Zymot plate. On day 2 fertilization was observed, and on days 5, 6 and 7, it was observed who reached the blastocyst stage were vitrified. The amount of blastocysts formed in each cycle was compared using a chi-square test, using RStudio^®^ software.

**Results:** A total of 6 patients were selected for the study. The maternal mean is 37 years, and male factors are associated with oligoasthenoteratozoospermia. Patients received the same stimulus medication in both cycles, and a similar amount of oocytes was captured in the first and second cycles. In the first cycle, where the seminal preparation was performed using a density gradient or swim- up, sperm were injected into a total of 42 oocytes, and of these, 7 reached the blastocyst and were vitrified, in the second cycle, using the Zymot technique. , a total of 32 oocytes were injected, and of these, 20 reached the blastocyst and were vitrified. The results show that using the new technology for sperm selection significantly improved results. There was a statistical difference between the results observed using conventional techniques and the use of the Zymot plate (p<0.05).

**Conclusion:** Our data suggest that seminal preparation with Zymot has the potential to increase the amount of embryos formed. This data is based on a total of 6 patients and 12 cycles. A more in-depth study of the impact of seminal preparation using the Zymot plate, when compared to other techniques, is important for a better understanding of the effectiveness of this new technology.

## P-43. Evaluation of DuoStim strategy in patients with poor ovarian response in a private Human Reproduction clinic

**Amanda Lopes de Faria^1^, Luisa Silva Carvalho Ribeiro^1^, Rivia Mara Lamaita^1^**

*^1^Rede MaterDei*

**Objective:** Show the results of the DuoStim strategy in patients with poor ovarian response. The main outcomes were oocyte quantity and quality and embryo development.

**Methods:** Cross-sectional study including 27 patients treated in a private Human Reproduction clinic from September 2019 to March 2022. We used the POSEIDON criteria to classify patients as poor responders. The choice to undergo the DuoStim protocol was based on a shared decision between doctor and patient. Data were collected from electronic medical records. Statistical analysis was performed using the t-Test. The following protocol was used for ovarian stimulation: high-dose (300 UI) recombinant FSH and LH (Pergoveris^®^, Merck^®^) from the 2^nd^ or 3^rd^ day of the menstrual cycle until the trigger date. GnRH agonist starting on day five or six of ovarian stimulation or when the ultrasound showed follicles above 13 or 14 mm. For the trigger, triptorelin at a total dose of 0.2 mg (Gonapeptyl^®^ Daily, Ferring^®^).

**Results:** The total number of eggs collected in the follicular phase was 116 and in the luteal phase 161 the average in the luteal phase was also higher, with statistical significance (mean follicular phase 4.3 and middle luteal phase 6.0, *p*<.05). There was no statistically significant difference between the mean of mature eggs collected in the follicular and luteal phases. Analyzing separately by POSEIDON groups, there was no statistically significant difference between the mean oocytes obtained after a puncture in the follicular phase and luteal phase or the number of mature oocytes between them. Group 3 was not submitted to individual analysis due to the n of a patient. There was no difference in fertilization rates comparing eggs obtained in the follicular or luteal stimulus. Regarding embryos, the mean number of embryos obtained after the second stimulus was higher than that obtained after the first stimulus (1.7 and 0.9, respectively, *p*<.05). This statistical difference was maintained between patients in the POSEIDON 2 group (mean embryos after follicular and luteal stimulation, respectively, 0.8 and 1.6, *p*<.05). There was no difference with the mean embryos obtained after follicular and luteal stimuli in the POSEIDON 4 group. During the study period, there were 13 embryo transfers. Four culminated in a single and eutopic clinical pregnancy, but two resulted in early pregnancy losses while two (successfully developed until term, resulting in two living births. Three patients reported a negative ß-hCG, and the others lost to follow-up after transfer.

**Conclusion:** Our data analysis suggests that luteal phase stimulations can be of great value in improving the amount of oocyte retrieval from poor responders, with no worsening in its quality or maturation status. Also, oocytes obtained from luteal stimulations contributed more to the totality of embryos when compared to those from the follicular phase. The subclassification among POSEIDON groups did not change DuoStim outcomes. Despite its small sample and its cross-sectional analyses, our finds are consonant with those in the literature. The DuoStim approach is a promising strategy for poor responder patients.

## P-44. Influence of follow-up for assessment and adjustments of metabolic parameters on the emotional aspect of women undergoing Assisted Human Reproduction treatment

**Bruna Rafaela De Marchi Facio^1^, Cássio Leão Facio^1^, Ligiane Alves Machado-Paula^1^, Edilberto de Araújo Filho^1^, Leonardo Previato de Araújo^1^, Ligia Fernanda Previato de Araújo^1^**

*^1^Centro de Reprodução Humana de São José do Rio Preto*

**Objective:** To investigate the quality of life, level of anxiety and stress, before and after follow-up for evaluation and adjustments of metabolic parameters in women with indication for *in vitro* fertilization (IVF).

**Method:** This is a descriptive, quantitative and qualitative research. The participants were contacted by telephone by the psychologist of the Assisted Reproduction Center and those who agreed to participate in the research answered a form created on Google Form with questions about how the participant felt about their health, how they classified their quality of life, level of anxiety and stress before and after follow-up for assessment and adjustments of metabolic parameters in assisted reproduction treatment. It was also evaluated how they felt with the proposed changes in the follow-up. The study included 24 married women, aged between 29 and 46 years (mean age 38 years).

**Results:** The answers about religion indicated that 2 (8.33%) consider themselves without religion; 3 (12.5%) spiritists; 4 (16.67%) Evangelicals and 15 (62.5%) Catholics. One has completed high school (4.2%); 23 (95.83%) have higher education, and of these 23, 15 (65.22%) are postgraduates. All work. Regarding the reason for seeking treatment, 21 (87.5%) were female; 1 (4.17%) female and male and 2 (8.33%) without apparent cause. At the time of the interview, 1 (4.2%) was on ovarian stimulation; 1 (4.2%) carried out the follow-up of the follicles by ultrasound; 3 (12.5%) after oocyte collection; 7 (29.2%) awaiting test results and 12 (50%) already had positive Beta hCG after transfer and of these 12, one became pregnant naturally before stimulation. Regarding the practice of physical activity, 17 (70.83%) perform it at least twice a week and 7 (29.17%) do not perform it. All were in metabolic follow-up and 1 (4.17%) in follow-up for 2 years; 12 (50%) 1 year ago; 3 (12.5%) for 5 months and 8 (33.33%) for 3 months.

When answering about health, 1 (4.2%) considered their health to be bad; 4 (16.7%) excellent; 9 (37.5%) good and 10 (41.7%) very good. When comparing with a year ago, before the follow-up, 2 (8.3%) considered that their health was almost the same; 10 (41.7%) a little better and 12 (50%) much better. The level of anxiety before the metabolic follow-up in 1 participant (4.2%) was considered low, 8 (33.3%) moderate and 15 (62.5%) high. This post level for 3 of them (12.5%) was considered low; 7 (29.2%) high and 14 (58.3%) moderate. As for the level of stress before the metabolic monitoring in 3 (12.5%) it was considered low; 8 (36.4%) was considered high and 13 (54.2%) was considered moderate, and the post for 7 (29.2%) was considered low; 4 (16.7%) high and 13 (54.2%) moderate. The quality of life before the metabolic follow-up of 3 (12.5%) was considered very good; 6 (25%) was considered bad and 15 (62.5%) was considered moderate, and the post for 1 (4.2%) was considered bad; 9 (37.5%) were considered moderate and 14 (58.3%) were considered very good. Psychological follow-up during preparation for IVF was performed by 10 patients (41.67%) and 14 (58.33%) did not. All participants (100%) felt better after the dietary changes and metabolic assessment and perceived improvement in sleep, gut health, and overall sense of well-being.

**Conclusion:** The follow-up for evaluation and adjustments of metabolic parameters brought improvement to the body and also to the psychological and emotional aspects, reducing anxiety, stress and bringing an improvement in the quality of life of the interviewees. Participants reported improvement in disposition, food awareness, mood, and the quality of their gametes, and the success of their treatments through Assisted Human Reproduction was remarkable for them.

## P-45. Analysis of ovarian stimulation outcomes, fertilization of 527 oocytes and blastulation rate with different recombinant follicle stimulating hormones (rFSH) associated with purified menotropin (hMG)

**Camila Dutra de Souza Francisquini^1^, Vinicius Bonato da Rosa^2^; Samara Artuso Giacomin^1^, Alessandro Schuffner^1^**

*^1^Conceber Centro de Medicina Reprodutiva*

*^2^Brown Fertility - Florida Fertility Clinics*

**Objective**: The objective of the present study was to compare the response to ovarian stimulation with the recombinant follicle stimulating hormones (FSH): Gonal-F^®^ (Alpha Folitropin) and Rekovelle^®^ (Delta Folitropin) in association with purified menotropin (hMG) Merional^®^ or Menopur^®^.

**Methods:** In this retrospective study, results of ovarian stimulation, oocyte recovery, fertilization and blastocyst formation rate were compiled in patients undergoing *in vitro* fertilization (IVF) between the years 2021 and 2022. As inclusion criteria, we only used patients aged between 30 and 45 years, regardless of the marital infertility factor, and the oocytes were recovered by means of follicular aspiration 35 hours after the trigger (hCG) and submitted to intracytoplasmic sperm injection (ICSI) with semen from fresh ejaculate. Patients underwent ovarian stimulation with antagonist protocol and were divided into two groups: Group 1 (n=28 patients) - Rekovelle^®^ + hMG (Merional^®^ or Menopur^®^) and Group 2 (n=56 patients) - Gonal-F^®^ + hMG (Merional^®^ or Menopur^®^). The averages for patient age (years), number of follicles above 12mm on the triggering day, number of retrieved oocytes, number of mature oocytes and number of fertilized oocytes were compared between the groups, and the averages were compared by the statistical test for analysis of variance (one-way ANOVA) with Tukey’s test, considering the statistical *p* of 0.05. The blastulation rate was compared between groups by the Z-test statistical method for 2 proportions, adopting the significant *p*<0.05.

**Results:** The results showed that there was no statistical difference (*p*>0.05) between the groups for the mean age of the patients submitted to the ovarian stimulation protocol, demonstrating the homogeneity between them (Rekovelle^®^ + hMG: 37.9±3.9 years and Gonal- F^®^ + hMG: 38.9±2.8 years). Furthermore, there was no statistical significance (*p*>0.05) between the Rekovelle^®^ + hMG and Gonal-F^®^ + hMG groups when comparing the number of follicles greater than 12mm on the triggering day (9.2±6.1 and 8.0±5.6, respectively), also when analyzing the mean number of oocytes recovered from ovarian pick-up (Rekovelle^®^ + hMG: 8.2±6.3 and Gonal-F^®^ + hMG: 7.2±5.5, p>0.05). For the number of mature oocytes, the Rekovelle^®^ + hMG group had a mean of 6.7±4.9 oocytes and the Gonal-F^®^+hMG group had a mean of 6.0±4.7, with no statistical difference between them (p>0.05). After 16 to 20 hours after ICSI of a total of 527 mature oocytes, a check for fertilization of the oocytes was performed, punctuated by the presence of 2 pronuclei in the cytoplasm of the oocyte and 2 polar bodies in the perivitelline space of the oocyte. A mean of 4.5±3.2 fertilized oocytes was obtained for the Rekovelle^®^ + hMG group and 4.4±3.4 for the Gonal-F^®^+hMG group, with no statistical difference between the groups (*p*>0.05). The blastulation rate, although higher in the Rekovelle^®^ + hMG group (57.1%) compared to the Gonal-F^®^+hMG group (52.8%), were statistically similar (*p*>0.05).

**Conclusion:** Based on the above, we can infer that regardless of the recombinant FSH used (Rekovelle^®^ or Gonal-F^®^) associated with hMG, the same efficiency was obtained in relation to the results of ovarian stimulation, oocyte recovery, fertilization and blastulation rate.

## P-46. Follicular aspiration in a patient carring ectopic ovaries and Mayer-Rokitansky- Kuster-Hauser syndrome - a case report

**Ana Carolina Possebom^1^, Viviane Margareth Scatamburlo Niehues^1^, Isadora Terumi Saruhashi^1^, Kahisa Natiele Fontana^1^, Marcos Antonio Trippia^2^, Lidio Jair Ribas Centa^1^**

*^1^Androlab*

*^2^Instituto Roetgen diagnóstico*

**Objective**: a case report about bilateral ectopic ovaries, leading to transabdominal aspiration for oocyte retrieval.

**Methods:** a 29 years-old woman with Mayer-Rokitansky-Kuster-Hauser syndrome and bilateral ectopic ovaries that desires maternity due to replacement uterus and her own oocytes. Because of her ovarian position, the aspiration for oocyte retrieval was via transabdominal ultrasound. In order to perform the controlled ovarian stimulation and trigger, it was used gonadotrophins, ovarian inductor progesterone and dual trigger. Follicular growth follow-up was via abdominal ultrasound.

**Results:** 16 follicles were aspirated and 16 oocytes were recovered. 9 oocytes were submitted to intracitoplasmatic sperm injection (ICSI), of which 5 were fertilized. Embryos were maintained in blastocyst culture for embryo biopsy. 3 embryos were biopsied with euploidy results. Embryos were cryopreserved for transfer in the next cycle in the delivery uterus. The first cryopreserved embryo transfer (CET) had a negative result. The second CET was positive, and it is still and ongoing pregnancy.

**Conclusion:** It is crucial to a Human Reproductive specialist to know other options of aspiration, always on behalf of the patient. Thus, acquiring the ability to perform the procedure via the transabdominal route is a possible path and must be followed.

## P-47. Ovarian stimulation outcomes in patients with unexplained infertility undergoing *in vitro* fertilization (IVF) with different types of gonadotropins: morphological analysis of 615 oocytes

**Camila Dutra de Souza Francisquini^1^, Carolina Kleemann^2^, Vinicius Bonato da Rosa^3^, Samara Artuso Giacomin^1^, Alessandro Schuffner**

*^1^Conceber Centro de Medicina Reprodutiva*

*^2^Faculdade Evangélica Mackenzie do Paraná*

*^3^Brown Fertility - Florida Fertility Clinics*

**Objective:** The present study aimed to evaluate the influence of gonadotropin used for ovarian stimulation in *in vitro* fertilization (IVF) cycles on the number of follicles, mature oocytes and oocyte morphology in couples with unexplained infertility.

**Methods:** Therefore, a retrospective study was carried out in patients undergoing ovarian stimulation for IVF in the years 2018 to 2021, aged between 21 and 45 years, with unexplained infertility and at least 1 oocyte recovered after follicular aspiration. Patients underwent ovarian stimulation with an antagonist protocol and were divided according to the medication they received for follicular recruitment and stimulation, as follows: human menopausal gonadotropin (hMG); recombinant follicle stimulating hormones (rFSH) and rFSH associated with hMG. Among the groups, were compared the averages for patient age (years), total FSH cumulative dose (International Units - IU), number of follicles above 12 mm in triggering day, number of oocytes recovered, number of mature oocytes, number of morphologically normal oocytes and number of morphologically altered oocytes (both intracytoplasmic and extracytoplasmic defects), considering the statistical p of 0.05. The averages were compared using the statistical test for analysis of variance (one-way ANOVA) with the Tukey test, and rates (%) were compared between groups using the Z-test statistical method for 2 proportions.

**Results:** The results obtained indicate that there was no statistical difference (*p*>0.05) between the age groups submitted to therapies with rFSH, hMG or rFSH + hMG, demonstrating the homogeneity between the groups. Furthermore, it was found that the total dose of gonadotropin was higher (*p*<0.05) in patients undergoing ovarian stimulation with hMG (2879.1±885.5 IU) compared to those undergoing therapy with rFSH (1760.6±523.0 IU) and rFSH + hMG (1946.0±597.0 IU). In addition, there was no statistical significance (*p*>0.05) between the treatment groups when compared the number of follicles (>12mm) in triggering day (hMG: 11.0±5.8; rFSH: 13.3±5.1; rFSH + hMG: 10.8±5.3), as well as when analyzing the number of mature oocytes (hMG: 6.7±5.0; rFSH: 8.9±4.7; rFSH + hMG: 7.4±4.5; *p*>0.05). Therefore, we can infer that the hMG group required a greater amount of I.U. of gonadotropin for the same number of follicles and mature oocytes. However, there was a greater recovery of oocytes (12.6±5.3, *p*<0.05) in the group submitted to treatment with rFSH, in contrast to 9.4±6.2 oocytes recovered in the hMG group and 9.9±5.2 oocytes in the rFSH + hMG group. In relation to oocytic morphology, 64% of the oocytes in the rFSH group were classified as morphologically normal, 62% in the hMG group and 53% in the treatment with rFSH + hMG (*p*<0.05), that is, 47% of the patients undergoing treatment with rFSH + hMG showed morphologically altered oocytes, while this percentage was 36% and 38% in the treatment with rFSH and hMG, respectively (*p*<0.05).

**Conclusion**: Based on the results presented, we can conclude that for couples with unexplained infertility, ovarian stimulation with recombinant FSH results in greater efficiency, since with a lower total dose of gonadotropin used, there was a greater recovery of oocytes after follicular aspiration. Additionally, it showed a higher percentage of morphologically normal oocytes in relation to the groups: hMG and rFSH associated with hMG.

## P-48. Colocalization of EPPIN and its potential interactors GAPDS and AKAP4 in mouse spermatozoa during capacitation

**Natália Calixto Miranda Santos^1^, Noemia Aparecida Partelli Mariani^1^, Gledson Vinicius Miranda dos Santos^1^, Alexandre Dorth Andrade^1^, Hélio Kushima^1^, Leandro Alves dos Santos^2^, Luciane Alarcão Dias-Melicio^3^, Erick José Ramo Silva^1^**

*^1^Department of Biophysics & Pharmacology, Institute of Biosciences, São Paulo State University (UNESP), Botucatu-SP, Brazil*

*^2^UNIPEX - Research Experimental Unity of Medical School (FMB), São Paulo State University (UNESP), Botucatu-SP, Brazil*

*^3^Department of Patology, Medical School (FMB), São Paulo State University (UNESP), Botucatu-SP, Brazil*

**Objective:** Almost half of the male infertility cases are idiopathic. Alterations in post- testicular sperm maturation, such as sperm maturation in the epididymis and capacitation in the female reproductive tract, which promotes sperm fertilizing ability, could be responsible for at least part of these idiopathic cases of male infertility. It is recognized that protein-protein interactions (PPI) play essential roles in promoting sperm maturation and function. The sperm binding protein Epididymal Protease Inhibitor (EPPIN) is a central hub for a PPI network in spermatozoa during post-testicular maturation. EPPIN expression in spermatozoa is positively correlated with maturation in the epididymis. After ejaculation, the seminal plasma protein semenogelin-1 (SEMG1) binds EPPIN on the sperm surface leading to transient inhibition of sperm motility and capacitation. Sperm motility is acquired after the prostate-specific antigen (PSA) cleaves SEMG1. Single nucleotide polymorphisms in the *EPPIN* gene have been associated with idiopathic male infertility. These findings showed the crucial roles of EPPIN on sperm motility and male fetility. Our previous study using the mouse as a translational model pointed to novel putative EPPIN interactors in mature spermatozoa, including glyceraldehyde 3-phosphate dehydrogenase (GAPDHS), a biomarker candidate for infertility, and A-kinase anchor protein 4 (AKAP4), a protein of fibrous sheath of the sperm flagellum essential for motility. Herein, we aimed to evaluate the colocalization of GAPDHS and AKAP4 with EPPIN in mouse spermatozoa during capacitation.

**Methods**: Adult Male Swiss mice (90-120 days old) were euthanized, and their cauda epididymides were dissected for isolation of spermatozoa using the “swim-out” method. Aliquotes of sperm samples were or not submitted to *in vitro* capacitation for 1 h (95%/5% CO_2_/air). Another group of male mice was mated with estrous-induced females to isolate spermatozoa from different regions of the female reproductive tract (vagina, proximal uterus, distal uterus, and oviduct), representing *in vivo* capacitation. The sperm smears were processed for immunofluorescence assays using antibodies to assess the colocalization of EPPIN with GAPDHS and AKAP4, later analyzed by confocal microscopy (CEUA/IBB 5402261121 and 5219150420).

**Results:** We demonstrated that EPPIN is localized in the sperm head (anterior acrosome and post-acrosomal region) and flagellum (abundantly in the midpiece and in the proximal region of the principal piece near the annulus) in non-capacitated spermatozoa. We observed a similar staining pattern when spermatozoa were submitted to *in vitro* capacitation or isolated from all regions of the female reproductive tract. GAPDHS is localized only in the principal piece of the flagellum, whereas AKAP4 is present in both midpiece and principal piece with eventually positive-immunostaining in the anterior acrosome and equatorial segment of the head. EPPIN colocalized with GAPDHS in the principal piece of the flagellum in both non-capacitated and capacitated spermatozoa. EPPIN colocalized with AKAP4 in the midpiece and partially in the principal piece of the flagellum. Curiously, EPPIN and AKAP4 did not colocalize in the sperm head.

**Conclusion**: Our findings clarify that EPPIN is not lost during the sperm journey to fertilization. Its colocalization with GAPDHS and AKAP4 suggests that the EPPIN PPI network may regulate sperm motility and male fertility via multiple mechanisms. Further studies are needed to evaluate the specific roles of EPPIN and its interactors on fertilization-associated events. Financial support: PIBIC/UNESP; CAPES and FAPESP.

## P-49. Oocyte retrieval in a patient with an important anatomy distortion - a Case Report

**Ana Carolina Possebom^1^, Regis Yukio Cho^1^, Isadora Terumi Saruhashi^1^, Kahisa Natiele Fontana^1^, Marcos Antonio Trippia^2^, Lidio Jair Ribas Centa^1^**

*^1^Androlab*

*^2^Instituto Roetgen diagnóstico*

**Objective**: transabominal follicular aspiration due to ectopic ovaries caused by large intramural myoma - a case report

**Methods**: a 39 years-old woman with low ovarian reserve and married to 36 years-old man, diagnosed with teratozoospermia. During the ultrassound exam, a large uterine myoma was identified, which could be and obstacle for the follicular aspiration.

**Results:** Because of the anatomic distortion, the oocyte retrieval was via transabdominal ultrasound. Three follicles were aspirated and two mature oocytes were colected. Both oocytes underwent intracitoplasmatic sperm injection (ICSI), and were fertilized. Embryos were maintained in culture to a blastocyst stage transfer plan. On day 6 of development, one of the embryos showed the beginning of blastulation, but on day 7, cell arrest was observed and there was no viable embryo for transfer.

**Conclusion:** In spite of the absence of embryos for transfer, which was expected according to the couple prognosis, this case highlights the importance to individualize patient follicular aspiration.

## P-50. Fertility in trisomy 21: a narrative review

**Ana Karoline Machado da Rosa1, Fernanda Perini Concatto^1^, Vítor Hugo Píccolo Bogo^1^, Maria Eduarda Leite Simões^1^, Carolina Comissoli Fernandes^1^, Mauricio Rouvel Nunes^2^**

*^1^Liga Acadêmica de Reprodução Humana - UFCSPA*

*^2^Programa de Pós-graduação em Patologia - UFCSPA*

**Objective:** This review aims to gather information about fertility in individuals with Down Syndrome.

**Methods:** The present study is a narrative review of the literature searching the PubMed and EMBASE databases for the descriptors MeSH: “infertility”, “down syndrome”, “trisomy 21”, “47,XY,+21” e “47,XX,+21”.

**Results:** A total of 588 articles were found, removing duplicates and unavailable articles, by the eligibility criteria 6 studies remained for analysis. According to the findings for this study, men with Down Syndrome are considered in most cases infertile, as researchers address hormonal aspects, morphological changes in the gonads such as Sertoli and Leydig cell dysfunction, abnormal spermatogenesis, azoospermia or oligospermia, physical and psychological factors related to mental retardation, as well as social problems as the source of the problem. This low number of spontaneous conception in men with DS may be due to the small percentage of patients who are sexually active or wish to have children, but it may also be due to gonadal dysfunctions. Although this patient profile is considered infertile, cases of paternity by parents with Down Syndrome have been described in the literature. Women, on the other hand, are confirmed fertile, and many cases of maternity have been reported. The literature only mentions the lower number of ovarian follicles by these patients, related to a deficiency in the production of the hormone estrogen, a factor that induces early entry into the menopausal period.

**Conclusion:** Despite advances in the lines of scientific research that seek their mapping as well as their possible treatments, evidence regarding the reproductive health of individuals with trisomy is scarce. However, case reports demonstrate that there is a possibility for these individuals to procreate and conceive chromosomally normal children.

## P-51. Impact of ovarian stimulation and *in vitro* fertilization in adult mice offspring: physiological indexes

**Caroline Mantovani da Luz^1,2^,Thalita de Souza Berteli^1,2^, Marília Alves Caetano^1,2^, Rui Alberto Ferriani^1,2^, Paula Andrea Navarro^1,2^**

*^1^Department of Gynecology and Obstetrics, Human Reproduction Division, Ribeirão Preto School of Medicine, University of São Paulo, 3900 Bandeirantes Avenue, Ribeirão Preto, SP, Brazil.*

*^2^National Institute of Hormones and Women’s Health (INCT), National Council for Scientific and Technological Development (CNPq), Porto Alegre, RS, Brazil.*

**Objective:** Evaluate the physiological indexes and the reproductive parameters in adult mice offspring obtained through ovarian stimulation and *in vitro* fertilization (IVF).

**Methods:** An experimental study using a mouse model, approved by the ethics committee on the use of laboratory animals. Natural group; offspring from natural mating cycle and in vivo fertilization. To verify the reproducibility of the results and maintain genetic variability were used three mice couples for the formation of the Natural group. IVF group; offspring from superovulated females and IVF from fresh oocytes. Follow up until the 20th week of offspring for both groups. At weaning (fourth week), the anogenital distance was measured. Body weight was monitored from the 4th to the 20th week. Systolic blood pressure (SBP) was measured by tail plethysmography (10th, 15th, and 20th weeks), and glucose, cholesterol, and triglycerides level were measured by point of care methodology (16th and 18th weeks). After euthanasia (21st week), the weight of organs was analyzed, moreover glucose, total cholesterol, and triglycerides level were measured by colorimetric methodology, and in males, sperm analyses were performed.

**Results:** The three mice couples gave birth to 12, 14, and 14 pups in the Natural group. After weaning, six (three females and three males), eight (four females and four males) and eight (four females and four males) offspring were randomly chosen from each litter. In the 15th week, one male died. Thus, the Natural group had 21 animals (11 females and 10 males). In the IVF group, the fertilization rate was 76% (307/402), blastocyst formation 60% (73/121), pregnancy 75% (3/4), and live births 31% (20/64). The three females that became pregnant gave birth to six (three females and three males), eight (four females and four males), and six (three females and three males) pups. In the 10th week of life, two sibling males died. Thus, the IVF group had 18 animals (10 females and eight males). Differences were observed between the Natural and IVF offspring. The weight of the animals showed differences when compared regardless of sex and stratified by sex. The animals in the IVF group presented higher body weight when compared to Natural group. The SBP (10th week) and glucose level (16th week) were higher in males from the IVF group than in the Natural group. The triglycerides levels (16th week) and total cholesterol levels (after euthanasia) were higher in females from the IVF group than Natural group. These findings could suggest a potential increase in cardiovascular risk in the IVF group, which needs to be better elucidated by expanding the sample size and evaluating additional outcomes. The females of the Natural group had a shorter anogenital distance than the females of the IVF group. When analyzing the sperm parameters of males from both groups, we observed differences only in sperm morphology. The IVF group had a lower percentage of morphologically normal sperm than the Natural group. No differences were observed between groups regarding the weight of abdominal fat and femoral muscle, nor in the reproductive organs weight. **Conclusion:** This study provided relevant inquiry on the impact of ovarian stimulation and IVF on adult mice offspring. Given the obstacles and delay in obtaining clinical data, such findings have potential applicability in human-assisted reproduction.

**Financial Support:** The National Council for Scientific and Technological Development (CNPq), grant number 426486/2018-8. National Institute of Hormones and Women’s Health (INCT-Hormona), grant number 465482/2014-7.

## P-52. Using CHLOE-EQ to automatically monitor embryo development, identify abnormal embryos and monitor Vienna Consensus Key Performance Indicators (KPIs)

**Iris de Oliveira Cabral^1^, Adelino Amaral Silva^1^, Antônio César Paes Barbosa^1^, Hitomi Miura Nakagawa^1^, Adriana Brualla^2^, Alexa Zepeda^2^, Cristina Hickman^2^, Bernardo Moura^3^, Edson Lo Turco^3^**

*^1^Genesis*

*^2^Fairtility*

*^3^Embriologica*

**Objective:**To automatically assess Vienna Consensus KPIs and identify abnormal embryos (pronucleate and cell division abnormalities) and understand the implications towards pace of embryo development.

Methods: Following insemination, 387 zygotes from 41 patients were cultured in a time- lapse incubator (Embryoscope, Vitrolife, Sweden). CHLOE-EQ (Fairtility, Israel) was used to automatically assess time-lapse videos. CHLOE-EQ is an Artificial Intelligence (AI) based assistant that supports embryo evaluation. Embryos were cultured following routine clinical procedures. CHLOE-EQ was used to identify pronucleates (PNs), Direct Unequal Cleavage (DUCs), blastulation and morphokinetic parameters. Morphokinetic development for different PN anomalies were assessed using Kruskal Wallis. Proportion of anomalies versus blastulation were assessed using Chi-square. Key performance indicators were compared with competency value as published in the Vienna Consensus (ESHRE SIG et al., 2017).

Results: Of the 387 zygotes (average 9 zygotes per patient), CHLOE-EQ was able to assess PN count in 97.4% of the zygotes. Normal fertilisation rate (2PN/zygotes assessed) was 66% (247/377); polyploidy rate (3PN/zygotes assessed) was 5.6% (21/377); 1PN rate was 5.8% (22/377); Degeneration rate was 8% (31/377) and fertilisation rate (two or more PN/zygotes assessed) was 71%. Blastulation per cleaved embryo was 50% (140/281). All of these rates were at least within the competency range according to the Vienna Consensus. The 83 DUCs were identified in 28% (70/247) of 2PNs, 41% (9/22) of 1PNs and 19% (4/21) of 3PNs. In this data set, none of the DUCs were able to form a blastocyst, whilst non-DUCs had a 71% (140/198) blastulation rate.Severe fragmentation was observed in 2% (5/247) of 2PNs and 14% (3/22) of 1PNs and none of the 3PNs. 1PN embryos were less likely to blastulate than 2PN and 3PN embryos [1PN: 24%(4/17) vs 2PN: 52% (126/244) vs 3PN: 50%(10/20); *p*<0.05]. 2PNs had significantly faster morphokinetic median timings to t2 (*p*=0.046), t3 (*p*=0.008), t4 (*p*<0.001), t5 (*p*<0.001), t6(*p*<0.001), t7(*p*<0.001), t8(*p*<0.001), t9(*p*<0.001),

tM (*p*=0.026). However, this difference was no longer significant at the blastocyst stages [tsB (*p*=0.055), tB (*p*=0.3) and teB(*p*=0.3)]. 1PN embryos that blastulated had equivalent pace of embryo development to 2PNs that blastulated.

Conclusion: With no human input, CHLOE-EQ is able to automatically process time-lapse videos into data that is useful for monitoring laboratory operational KPIs as well an opportunity to learn from the data. Many IVF clinics follow the policy of a blanket discard of 1PN and DUC embryos which are not considered for treatment. However, recent publications have suggested that these unusual embryos may be considered for treatment when they follow a normal pace of embryo development all the way to the blastocyst stage. CHLOE-EQ can help identify these embryos, and further identify when these embryos are developing normally by automatically tracking their morphokinetics and supporting the embryologist in determining when unusual embryos should be considered for treatment, thus avoiding the discarding of potentially viable embryos and protecting the cumulative chances of success to individual patients.

## P-53. Time lapse, evolution of embryos cultivated in the MIRI TL” time lapse model: Primary outcomes compared to the ideal average time preestablished by manufacturer. *In Vitro* Fertilizartion (IVF) center “Laboratório de Reprodução Assistida” Federal University of Goiás ( LABREP-HC/UFG)

**Arsele Yvan Tcheuffa^1^, Mário Silva Approbato^1^, Fabiana Carmo Approbato^1^**

*^1^Universidade federal de Goiás*

**Objective**: to study the first outcome of embryos cultivated in a time lapse present in an IVF center and compare with the ideal data preestablished by the “miri TL” manufacturer.

**Methods**:395 embryos of 50 pacients undergoing IVF treatement in LABREP were cultivated in a time lapse from 2019 to 2022. Pacients were divides into two groups: group 1, 35 years old or younger and group 2 more than 35 years. It was identified the time to reach two(T2), four(T4) cells division, and morula/blastocyst time (tMB) then compared to the ideal time preestablished by the “MIRI T manufacturers.

**Results:** the average age was 37 years old (± 4); 76% of the embryos came from patients up to 35 years, only 23% of patients 35 years or younger. More than 26% of the patients had a total number of progressive mobile sperm near to zero. 35% of our patients had severe male factor. only 23% of our patients had a normal semen. In comparation with the ideal average time predetermined, Considering all (age) patients, The average time for this center to T2 was 30.7hours (2.7 hours later), 44.4hours (7 hours later) to T4 and 87.1hours (7.3 hours later) to tMB. In the group of patient of 35 years or younger, 52% reached the ideal average time to T2, 42.8% T4 and 14% reached tMB . In the group of patients over 35 years, 62.5% reached T2, 50% T4 and 43% to tMB in the ideal average time. Discussion: This study showed a low fertilization rate compared to 87.1% of Mizobe Y et al 2017. event when we separated patients in two groups we have 29% of fertilization for patients 35 years or younger against 24,6% for patients over 35 years old. Embryos in this study displayed a later first division average time compared to the ideal time of the “MIRI TL”. This results can be explained by the quality of our sample (more than 76% of patients over 35 years, poor responders and 35% of all patients with severe male factor who didn’t accept semen donation) Kirkegaard K et al’s have shown that early-cleavage embryos had strong developmental potential. Mizobe Y et al 2017 found that the blastocyst and good-quality blastocyst formation rates were high for the embryos that exhibited an early first division (before 25.90 hours), he also declares that good- quality blastocyst formation rate was not improved by the selection of embryos that displayed a late first division with an early synchrony to first division, possibly as more of these embryos featured morphological abnormalities.Otsuki J et al( 2004) observed that the decision to perform an early embryo transfer must be taken by at least day 2 of culture, as delay in starting luteal support can reduce the pregnancy rate.^4^

**Conclusions**: 57.25% of all embryos in this center reached the average time preestablished by“ MIRI TL” to T2, 46% to T4 and 28.5% to tMB. This embryos shall be transferred first. The result exposed user’s profile of this center, in other hand this profile explains the results and prognostic of this study. “olders patients with poor sperms”, produce poor quality embryos identifiable in the early division T2 and T4. Knowing that can be helpful for appropriate and quick clinical decision.

## P-54. Effect of follicular flush on embryo development of patients with low ovarian reserve

**Karla Pacheco de Melo^1^, Luciana Semião Francisco^1^, Amanda Sartor^1^, Ingrid Sene Rosa^1^, Luana Carolina Oliveira Rodrigues^1^, Lorena Ana Mercedes Lara Urbanetz^2,3^, José Maria Soares Junior^2^, Joji Ueno^2,3^**

*^1^LabFIV/EPM - Unifesp*

*^1^LabFIV/ EPM - Unifesp*

*^2^HCFMUSP*

*^3^Clínica Gera*

**Objective**: To assess whether flushing is effective in retrieving oocytes in patients with low ovarian reserve, as well as its effect on embryo development.

**Methods**: Retrospective study with data collected between 2020 and 2022. Oocyte retrieval by flushing was performed in patients with low ovarian reserve, who were included in the study according to the Bologna Criteria. Follicular flush had already been implemented in the laboratory by doctors who observed difficulty in recovering oocytes in patients with low ovarian reserve. Data on fertilization rate, presence of good embryos at D3 and blastocyst rate were compared with women of the same mean age who underwent an *in vitro* fertilization procedure in the laboratory.

**Results**: Data analysis showed that follicular flush is effective in recovering oocytes up to the third syringe washing the follicle, where 30% of the total oocytes collected are recovered in the first syringe, 34% are recovered in the second syringe and 20% in the third. As for the analysis of embryonic development, we observed that patients with a mean age of 39 years, matched by age as patients with low ovarian reserve, have a lower fertilization rate compared to patients who underwent follicle flush (t(231)=- 1.924; *p*<0.05), however, patients with low reserve have a lower average of good embryos on the third day of development (t(231)=3.630; *p*<0.05). Comparison of blastocyst formation rates showed no statistical difference between groups. We also observed that one patient, whose oocyte was recovered by flushing, is pregnant (1 in 6 embryo transfers performed, with 2 clinical pregnancy in the selected period).

**Conclusion**: Despite not altering blastocyst formation rates, follicular flush is effective in retrieving oocytes from patients with few follicles that were not aspirated during puncture. We observed that the oocyte collected is usually mature and has better fertilization rates compared to patients of the same mean age. Although the embryos formed have a lower quality on the third day of development, this decreased quality does not impact the blastocyst formation in these patients. It is important to remember that follicular flush may offer a chance to patients with low reserve, decreasing the time of infertility treatment and the need for a new ovarian stimulation.

## P-55. How reliable is PGT-A? A case report of pregnancy with triploid fetus after normal embryo biopsy

**Alvaro Petracco^1^, Mariangela Badalotti^1^, Isadora Badalotti-Teloken^1^, Catarina Heckmann Petracco^1^, Marta Hentschke^1^, Ricardo Azambuja^1^, Maria Teresa Sanseverino^1^**

*^1^FERTILITAT*

**Objective**: The risk of developing embryos with chromosomal abnormalities increases with maternal age due to oocyte aging. Preimplantation genetic testing for aneuploidies (PGT-A) identifies numerical chromosomal alterations, improving the chance of a healthy baby and shortening the time to pregnancy.

**Methods:** Case report study that included one patient. Data were collected from electronic records from a prospective database. The study was approved by the ethics and research committee of PUCRS, Porto Alegre.

**Results:** CASE REPORT: Couple, both 43 years old, healthy, with a history of pregnancy at 22 years old with spontaneous abortion (12 weeks) and a 1-year *in vitro* fertilization (IVF) cycle without pregnancy (7 eggs, no embryo to transfer). A new IVF cycle with PGT-A was performed. After ovarian stimulation, four mature eggs were retrieved and inseminated by intracytoplasmic sperm injection; 18 h after insemination, the four oocytes exhibited two pronucleus (normal fertilization); three reached the blastocyst stage at D+5 and were biopsied and analyzed by New Generation Sequencing (NGS). The analysis showed one euploid embryo (46, XX), one complex aneuploid embryo (+8, +18, +21), and one chaotic aneuploid embryo. One year later, the euploid embryo was transferred, which resulted in clinical pregnancy. Ultrasound at 12 weeks showed reduced fetal movement, holoprosencephaly, inconclusive nasal bone, and ductus venosus pulsatility above the 95th percentile. A non-invasive prenatal genetic test was performed, which showed normality for the 24 chromosomes and the absence of the most common microdeletions. Amniocentesis was performed at 15 weeks of pregnancy, and the fetal karyotype showed triploidy (69, XXX). The pregnancy was interrupted 16 weeks.

**Conclusion**: The PGT-A is an important tool to increase the chances of giving birth to a healthy baby. Although the PGT-A is around 98% accurate, there is a 1-2% risk of a false positive or false negative concerning numerical aneuploidies. Among the limits is the possibility that the test is not informative due to the absence or poor quality of the DNA of the collected cells. Other limitations include the impossibility of identifying some alterations, including polyploidies, as in the case described, parental disomy, balanced structural anomalies, or structural anomalies smaller than the platform can identify. Chromosomal copy number analysis by NGS does not require a large amount of data as it is determined using Shallow Whole Genome Sequencing (sWGS). This data can be obtained within 24 hours, and sWGS by NGS allows the simultaneous analysis of a large number of samples, thus reducing the cost per sample. On the other hand, this method is limited in detecting polyploidy, a common genetic cause of miscarriage. Whereas triploidy containing a Y chromosome can be detected by calculating the sex chromosome ratio, polyploidy without a Y chromosome, such as 69.XXX and 92.XXXX is very difficult to distinguish from 46.XX using NGS-based sWGS. Several studies have tried to overcome limitations with the use of additional techniques for the detection of triploidy, such as the QF-PCR analysis, which allows identifying the parental origin of the chromosomes. In all situations of error or impossibility of diagnosis, an embryo with a genetic abnormality will be transferred. Then, it is crucial to inform the patient about the risks and limits of the exam.

## P-56. Influence on Body Mass Index in Ovulation Frequency in Infertile Women

**Arsele Yvan Tcheuffa^1^, Fabiana Carmo Approbato^1^, Mário Silva Approbato^1^, Beatriz Bacheschi do Carmo Benetti^1^**

*^1^Universidade federal de Goiás*

**Objective:** Effect of weight variation on ovulation in patients being treated for infertility. To assess changes in physical parameters (BMI) over a wide age range (21-42 years) of 413 infertile women.

**Methods:** This was a cross sectional study in which 413 infertile women of Human Reproduction Laboratory (LabRep) from 2014 to 2019. This work was the result of part of the doctoral thesis. They were divided into five age groups. The control group was the age group from 21 to 28 years old (G1). As a factor of exposure, the age of the patients (aging) was categorized in different age groups G1 (21 to 28), G2 (29 to 33), G3 (34 to 36), G4 (37 to 39) and G5 (40 to 42) years. Exclusion factors were patients with surgery for Bilateral Tubular Ligature (BTL), removal of ovarian endometriomas, oophorectomized, with reductive gastro-duodenal surgeries for weight reduction, with clinical hypo or hyperthyroidism or in knowing use of medications that alter the compared variables. Mann-Whitney statistics were used to compare the medians and the Chi-square was used to compare the percentages. The p rejection level was 5% (*p*=0.05). This study was approved by the Ethics Committee (CEP) of the Hospital das Clínicas (HC), number 2.964.622.

**Results:** In this study, the BMI of the 413 patients ranged from 17.3 to 41.7. The mean age of the patients was 34.9 years (±4.5sd). The BMI had a population average of 25.1 kg/m2 (±4.3sd). There was a significant difference in BMI only in groups G2 and G3 when compared to group G1.

**Conclusion:** Even with the BMI treatment limit of 31, 44 patients (10.6%) were treated despite not having reduced weight, after approximately one year of weight loss recommendations. In this study, BMI was significantly lower in groups G2 (29-33) and G3 (34-36) when compared to group G1 (21-28).

## P-57. Experience of 152 cases of Magnetic Resonance Hysteroresonance

**Júlia Garcia Greggio^1^, Rafaela dos Santos Pinto Ferreira^1^, Mariana Augusto Lisboa^1^, Leandro Accardo de Mattos^2,3^, Luiz Fernando Pina de Carvalho^4,5^**

*^1^University Nove de Julho - São Paulo, Brazil*

*^2^hospital israelita Albert Einstein*

*^3^Laboratório Alta excelência diagnóstica*

*^4^Baby Center - Center for Reproductive Medicine - São Paulo, Brazil*

*^5^College - Institute of Clinical Research and Teaching Development - São Paulo, Brazil*

**Objective:** The study aims to review 152 cases of patients who had been submitted to hysterosalpingography by magnetic resonance imaging (HSR-MR) and report the advantages of the exam in comparison to the conventional method. The exams currently used for infertility investigation are: pelvic ultrasonography, hysterosalpingography by X-ray (HSG-XR), the standard method, and hysterosalpingography by magnetic resonance imaging (HSG-MR), a promising technique that in many cases allows women to perform a single test and determine the cause of their infertility. The HSG-XR is currently the gold standard, although it exposes the female reproductive system to ionizing radiation and provides a limited evaluation for infertility cases, requiring additional tests and the consequent delay in diagnosis and beginning of treatment. The evaluation of the 152-case HSR-MR experience provides a practical and comparative overview of this technique, enabling advances in the current model of infertility research.

**Method:** A sample of 152 patients who underwent HSR-MR was obtained from the files of Clínica Baby Center Medicina Reprodutiva and of Alta Diagnósticos, which 95 were retrospectively collected from 2019 to 2021, and 57 were prospectively collected from June 2021 to July 2022. From the prospectively collected data, demographic characteristics - age, BMI, skin color, type of infertility, smoking, menstrual cycle - of 24 participants were analyzed, representing demographically the profile of the patients studied. A literature review on the subject was also conducted using the PUBMED research platform, searching for the keywords: hysterosalpingography, magnetic resonance imaging, infertile women and endometriosis. The pre-established including criteria were English or Portuguese language, articles published between 2012-2022, and tests performed in humans. 182 articles were found, of which 17 were incorporated into the experiment.

**Results:** The HSR-MR enabled the evaluation of tubal permeability, mucosal relief changes, ectasia, hydrosalpinges and adherential processes, evidencing permeability in 86.18% of right fallopian tubes and 79.61% of left fallopian tubes; presence of hydrosalpinx in 5.92% of right tubes and 11.18% of left tubes; 34.87% of alterations in right mucosal relief and 43.42% of alterations in left mucosal relief; 23.68% of ectasia in the right tube and 25.66% of ectasia in the left tube, with symmetric excretion in 47.37% of the patients. It also showed pelvic adhesions or contrast collection in the pelvis in 39.47% of the cases. Myomas were found in 25% of the patients; polyps in 7.89%; adenomyosis in 19.74%; synechia in 4.61%; endometriomas in 43.42% and deep endometriosis in 60.53%. The pain level reported by patients during the exam reveals a 1.81 weighted average pain grade, on a 0-10 numerical scale (0 no pain and 10 highest pain). The age range of 87% of the patients was between 30 and 40 years, with a mean age of 34.7 years. 95% of the indications were for primary infertility, 100% of them with regular menstrual cycle, lasting 5-7 days and 66% with BMI within normality.

**Conclusion:** HSR-MR is an innovating method, and demonstrates to be an adequate diagnostic imaging exam, free of ionizing radiation, with minimal pain and high accuracy for the major pathologies responsible for infertility, such as adenomyosis, leiomyomas, endometriosis and congenital abnormalities of the genital tract. It involves a technique with high spatial resolution, multiplanar reconstruction capacity, and therefore is highly accurate in the investigation of tubal permeability and intrauterine abnormalities, being the only method capable of identifying tubal alterations and endometriosis simultaneously. Thus, it has demonstrated to be a method of high potential for implementation as a gold standard technique in the study of female infertility, surpassing in several aspects the conventional examination (HSR-RX).

## P-58. Ultrasound guided cesarean scar pregnancy treatment: case report and literature review

**Eduardo Lima da Rocha^1^, Luanna Ferreira Ayala Farias^2^, Eduardo de Paula Miranda^1^, Sebastião Evangelista Torquato Filho^1^**

*^1^Sollirium Health Group*

*^2^Núcleo de Endometriose do Ceará*

**Objective:** To demonstrate the advantages of minimally invasive techniques in the treatment of ectopic pregnancy in uterine scar from cesarean delivery through a case report and review the literature.

**Methods**: Diagnosis of ectopic pregnancy can avoid complications such as hemorrhage due to uterine rupture. When performed in early stages of pregnancy, it allows minimally invasive therapy guided by TVUS. Ultrasound criteria for this diagnosis were described by Vial et al in 2000. TVUS-guided puncture was performed and followed by aspiration of the liquid content and injection of methotrexate directly into the gestational sac, under sedation, on an outpatient basis. A complementary intramuscular dose was given 48 hours after the procedure. The patient underwent serial control with TVUS and quantitative dosage of BHCG, demonstrating resolution of the pregnancy. The specific literature on this approach was reviewed.

**Results**: Despite representing the rarest form of ectopic pregnancy and the scarcity of data in the literature, evidence corroborates the advantages observed in this reported case, where TVUS-guided puncture and methotrexate infiltration was effective as treatment of ectopic pregnancy with a live embryo in cesarean uterine scar, with only one intralesional application, complemented by a single systemic dose, performed on an outpatient basis, without complications, without post-procedure discomfort, allowing return to activities 24h later, preserving fertility and avoiding surgical procedure.

**Conclusion**: Minimally invasive treatment of ectopic pregnancy in cesarean scar using TVUS-guided puncture and methotrexate infiltration is an effective, more comfortable and fertility-preserving technique. This technique should be considered as an alternative to conventional surgical treatment in patients in early ectopic uterine scar pregnancy.

## P-59. The impact of male age on embryo euploidy rate

**Mariangela Badalotti^1^, Ricardo Azambuja^1^, Adriana Cristine Arent^1^, Fabiana Mariani Wingert^1^, Isadora Badalotti-Teloken^1^, Gustav Peter Foerster^1^, Victoria Campos Dornelles^1^, Marta Ribeiro Hentschke^1^, Claudio Telöken^1^, Alvaro Petracco^1^**

*^1^Fertilitat*

**Objective:** Many studies evaluate the effect of increasing female age on embryo euploidy rate, suggesting a negative correlation between them. However, there are few reports about the influence of male age. This study aimed to evaluate the impact of male age on embryo euploidy rate.

**Methods:** Retrospective cohort study, using data collected from an electronic database. A total of 748 ICSI cycles (male age from 30 to 55 years old (yo) and female age from 30 to 45 yo) using fresh oocytes and ejaculated sperm performed between 2017 and 2021, were included. Couples with altered karyotypes and frozen embryos were excluded. Fresh embryo biopsies were performed at the blastocyst stage, and the analysis was made using Next Generation Sequencing. Firstly, the embryos were divided in groups according to the male age (30-34 years old, M1; 35-39, M2; 40-44, M3; 45-49, M4, and 50-55, M5), the euploidy rates were compared and a cutoff point was found. Then, to clarify female age’s impact on our results, the embryos were divided into two groups according to female age: young age (30-37 yo) and old age 38-45 yo). The age of 37 was selected after an analysis that determined this age as the cutoff point. Finally, we compared the male age groups with female age groups. Statistical analysis: chi-square test, ANOVA test, and linear regression considering p<0.05 significant.

**Results**: A total of 2260 embryos were analyzed, and 704 (31.3%) were euploid. The results according to male age showed a significant reduction in euploidy rate starting at 40 years, and another significant reduction after 45 years: M1 = 39.6%, M2 = 36.3%, M3 = 28.9%, M4 = 22.8% and M5 = 22.9%; *p* < 0.001 (M1 vs. M2, *p* = 0.358; M1 vs. M3, *p* = 0.003; M1 vs. M4, *p* < 0.003; M1 vs. M5, *p* = 0.001; M2 vs. M3, *p* = 0.002; M2 vs. M4, *p* < 0.001; M2 vs. M5, *p* = 0.002; M3 vs. M4, *p* = 0.036; M3 vs. M5, *p* = 0.115; M4 vs. M5, *p* = 0.855). The embryo euploidy rate comparing female age 30-37 yo (338/735 embryos) vs. 38-45 yo (370/1525 embryos) showed 46.0% vs, 24.3% respectively (*p*<0,001; OR=2.66, 95% C.I. = 2.20 to 3.22). In the younger women group, no differences on embryo euploidy rates were found when comparing the male age group 30-39 yo (229/496, 46.2%) vs 40-55 yo (109/239, 45.6%) (p=0.875) or 30-44 yo (269/643, 46.0%) vs. 45-55 yo (40/88, 45.7%; (p=0.910). In the older women group, the embryo euploidy rate decreased with increasing male age: 30-39 yo (167/585, 28.5%) vs 40-55 yo (203/940, 21.6%) (*p*=0.002; OR 0.69, CI 95% 0.54 to 0.87) and 30-44 yo (311/1170, 26.6%) vs. 45-55 yo (59/355, 16.6%) (*p* < 0.001; OR 0.55, CI 95% 0.40 to 0.75).

**Conclusion:** These data suggested that for women younger than 37 years old, the male age does not impact the embryo euploidy rate. However, for women older than 37 years old, the euploidy rate was negatively affected by male age over 40 and even more over 45 years.

## P-60. INFERTILE COUPLE’S EXPECTATIONS ABOUT PGT-A

**Karolyne Vale de Sá^1^, Fernanda Sanchez Bachega^1^, Gabriel Monteiro Pinheiro^1^, Marina Gelego Teixeira^1^**

*^1^Universidade Santo Amaro.*

**Objective:** To investigate infertile couples’ expectations about the use of preimplantation genetic testing for aneuploidies (PGT-A) on the understanding of the test indication and pregnancy perspectives after the application of the technique.

**Methods:** Exploratory descriptive and qualitative research conducted with 27 patients from an assisted reproduction clinic who had or will have embryos undergoing PGT-A. A 15-question questionnaire was administered to the patients using the “Google Forms” platform, after signing the Informed Consent Term digitally. The study met all the recommendations of the Ethics and Research Committee of the UNISA, being approved.

**Results**: The questionnaire obtained 27 answers, of which 88.9% were female, 88.18% are over 35 years old and 77.8% declared they do not have biological children. Regarding the demographic profile of the group surveyed, 59.3% said they identified themselves as white, 33.3% as brown, and 7.4% as yellow; as to religion, 37% declared themselves Christian, 5.26% spiritist, 2.7% Buddhist, and the remaining with no defined religion; as for the level of education, 48.1% said they had a post-graduate degree, 48.1% complete or attending to higher education, and 3.7% high school. Regarding the period of trying to get pregnant, 33.3% are trying for less than 1 year, 22.2% for 1-2 years, 22.2% for 2-3 years, and 22.2% for more than 5 years. Regarding the time of treatment, 63% have been in treatment for less than 1 year in treatment and 14.8% between 2 and 3 years. About the respondents’ knowledge about what the PGTA analyzes and the benefits of the test, the answers, in their majority were about the “genetic analysis of the embryo” or “selection of a healthy embryo”, demonstrating that there is basic knowledge about the purpose of the test, but not a full understanding of it. The majority of patients pointed out as the reason for the exam being that they are older than 39 years old, due to the drop in embryo quality and a considerable increase in the risk of genetic alterations. It is known that assisted reproduction treatment can be long and with uncertain results, with no guarantee of success. However, regarding the expectation about PGT-A, most of the of the interviewees showed confidence in relation to the exam and the belief that it can be effective for a healthy pregnancy without genetic alterations. More than 66% of participants stated that they either fully or partially believe that PGT-A can shorten the time between treatment and pregnancy. In fact, literature shows that despite live birth rates being similar between groups undergoing or not this embryo biopsy, the treatment time is approximately 4 months shorter among those those who had PGT-A. When asked whether performing the test meant a guarantee of successful pregnancy, 66.6% of the participants agreed totally or partially with the statement. However, although patients who underwent PGT-A generally present a lower rate of pregnancy loss and implantation failure, it is not possible to claim that the test guarantees the success of a pregnancy, because there are numerous factors involved for a healthy pregnancy to occur beyond aneuploidies.

**Conclusion:** The study showed a good expectation and confidence of patients in the benefits of PGT-A. However, despite a potential decrease in treatment time to pregnancy, especially among those with a history of repeated implantion failures or repeat miscarriages, the test is not a guarantee of success of assisted reproduction treatment and does not have a higher rate of live births, pointing to a need for greater understanding among couples with embryos that have undergone the test with the expected outcomes and objectives. A research presented with the limitation the low number of respondents.

## P-61. Fertility preservation in trans women: a case series

**Isadora Badalotti-Teloken^1^, Mariangela Badalotti^1^, Catarina Petracco^1^, Débora Farinati^1^, Adriana Arent^1^, Alvaro Petracco^1^**

*^1^Fertilitat, Reproductive Medicine Center.*

**Objective**: Gender transition implies hormonal treatment (HT) or surgery, both of which can lead to fertility problems. Fertility preservation options must be offered to transgender patients, preferably before the transition begins. Cryopreservation should ideally be done before the start of HT. However, it is possible to do it after treatment if the patient suspends the use of HT and if the testicles produce sperm. Trans women can have their semen frozen from the ejaculate, even if the seminal production has low quality. The objective of this work is to present three cases of seminal cryopreservation with a view to future parenting in trans women.

**Methods**: Report of 3 cases through the review of electronic medical records of a human reproduction clinic.

**Results: CASE 1**: 18-year-old patient, single, student with incomplete higher education. She asks for seminal cryopreservation before starting hormone therapy. The semen samples were collected by masturbation - 1st collection: sperm concentration 60 million/ml; 2^nd^ collection: sperm concentration 50 million/ml. The patient has been keeping the material frozen for 5 years. She has opted for post-Morten disposal. **CASE 2**: 21-year-old patient, single, student. She is interested in cryopreservation but has decided not to pursue any treatment. The semen samples were collected by masturbation - 1st collection: sperm concentration 160 million/ml; 2nd collection: sperm concentration 110 million/ml. She asked for the material to be discarded 3 months after the cryopreservation, as she was no longer interested in it. **CASE 3**: 22-year-old patient, single, autonomous worker, undergoing HT. Interested in cryopreservation before conversion surgery but did not want to stop the hormonal medication. The semen samples were collected by masturbation - 1st collection: sperm concentration 2.6 million/ml; 2nd collection: sperm concentration 3.0 million/ml. She has been keeping the material frozen for 3 years.

**Conclusions**: Two patients underwent cryopreservation before starting hormone therapy and had normal seminal characteristics. One patient was using hormone therapy, did not want to suspend it for cryopreservation, and presented oligospermia in the samples collected. Hormone therapy in transgender women consists of the use of estrogen and androgen suppressors. The two combined actions cause a decrease in GnRH, with a consequent decrease in LH and FSH, leading to testicular regression (Leydig cell atrophy and fibroblast proliferation) with a decrease in serum androgens that results in alteration or suppression of spermatogenesis. In the case of hormonal treatment, it is not known whether the alteration in spermatogenesis is complete, universal, related to demographic factors, or dependent on the dose, regimen, and time of exposure to estrogen therapy. However, studies show that more than half of the patients may have complete spermatogenesis damage, demonstrating the importance of being alerted about these alterations and preserving fertility before instituting treatment. For some trans women, collecting sperm by masturbation can be psychologically traumatic, as it may conflict with their female identity. The health team must be attentive and sensitive to this fact. If the patient is not comfortable collecting semen by masturbation, surgical collection could be an option. Issues related to the reproductive future should be discussed before beginning gender transition treatment, even if the desire for children is not present at the moment, and it can be a delicate process to address this issue.

## P-62. Infertility in the Brazilian Unified Health System: When and how to stop this being a reality for few

**Karolyne Vale de Sá^1^, Ana Alice Soares Orçay^1^, Clara Carolina Godoy da Silva^1^, Gabriel Monteiro Pinheiro^1^**

*1Universidade Santo Amaro*

**Objective**: To discuss the scenario and problems of Assisted Reproduction in the Brazilian Unified Health System (SUS).

**Methods:** Bibliographic review in the databases of Scielo and Pubmed platforms and analysis of legislations and ordinances regarding assisted reproduction in the public health systems of Brasil and other countries, such as Argentina, France, Spain, England and Canada.

**Results:** In Brazil, the State guarantees access to reproduction methods by the Federal Constitution and the Family Planning law 9263/96, offering conceptive and contraceptive methods and techniques. However, access to assisted reproduction techniques (ART) is still restricted in terms of socioeconomic profile, due to high treatment costs and limited access to information. The costs of highly complex ART depend on the technique and professional chosen, the woman’s age and other factors, ranging from R$8,500 to R$30,000 per cycle of *in vitro* fertilization (IVF). The high budget restricts access and, based on this, the ordinance N. 3149 of 2012 brings to couples who cannot afford their treatment in private clinics, the opportunity to perform it through the public health system. However, challenges still persist. Currently, only ten out of more than 180 assisted reproduction centers in the country perform highly complex procedures through SUS. Consequently, the waiting list to undergo the treatment is, on average, 3 to 4 years, a factor that influences the maternal age and the success of the treatment. In other countries that offer ART through the public health service, such as Spain, France, and Canada, the waiting time is around one year, while in England, it varies according to the place of residence, with an estimated average of 4 months. In South American countries, such as Argentina, the waiting list is similar to the one in Brazil. Regarding maternal age, the SUS reproduction centers have selection criteria ranging from 35 to 40 years old, as in Spain. In France and England, the maximum age is 42 years, while in Argentina, the limit is 44 years for procedures with own eggs. The maximum number of IVF cycles that can be performed varies according to the reproduction center, on average 2 to 3 attempts per couple, but there are centers that do not stipulate limits of attempts. The Brazilian scenario in this regard is similar to other countries, which guarantee an average of 3 highly complex cycles. In some reproduction centers covered by SUS, medications are not included, which implies difficulty of access for low-income couples.

**Conclusion:** In general, Brazil is similar in some criteria to other countries that offer ART through public health systems; however, the reduced number of sites that offer the treatment makes the waiting time significantly longer, representing a failure in the guarantee of family planning foreseen in the Constitution. This situation could be reversed with the increase in the number of centers able to serve by SUS, including teaching and research centers in this area and philanthropic entities. Moreover, the user paying for drugs and exams in some services becomes an obstacle to access, and may cause the user to give up the treatment. Still, to improve service’s coordination, Primary Health Care (APS) should be better trained to identify infertility and its early diagnosis, guidance on access to ART by SUS and referral. The guarantee of full access to reproductive rights in the country still faces obstacles, mostly socioeconomic, being necessary to expand both investments and the debate on the subject, considering the importance of assisted reproduction in family planning.

## P-63. Clinical characteristics of female homosexual couples with desire to conceive

**Mariangela Badalotti1, Marta Ribeiro Hentschke1, Isadora Badalotti-Teloken1, Victoria Campos Dornelles^1^, Débora Farinati^1^, Alvaro Petracco^1^**

^1^Fertilitat, Reproductive Medicine Center

**Objective**: The family formed by spouses of the same sex is a reality, and with the advancement of assisted reproduction techniques, so is conceiving with their own gametes. However, the number of patients who seek clinics is still small, and studies in this area are still scarce. In this way, the objective of this study was to present clinical characteristics of the attendance of female same-sex couples with the desire to conceive.

**Methods:** Cross-sectional study. Data from 44 couples who underwent 53 cycles of ovarian stimulation for *in vitro* fertilization (IVF) performed from 2016 to 2021 were included. Patients were aged between 19 and 56 years old. The appointments were carried out in an assisted reproduction clinic, by specialist doctors. Data were computed and presented as mean, number and percentage.

**Results:** Most couples were referred to the reproduction center by doctors (40.9%), followed by referrals by friends (29.5%), internet/social networks (15.9%), and other patients (6.8 %); 6.8% did not inform. As for the profession, most patients worked in the health area (31.8%), followed by the business area (12.5%), teaching (9.1%), law (5.6%), and customer service (5.6%). The age difference between the partners was found from the same age to 29 years of difference, with the average age difference being five years. The relationship time ranged from 1 to 16 years, with an average of 6.7 years. As for sexual and reproductive history, in at least 14 couples, one of the partners reported having had heterosexual sexual relations in the past; three patients already had children; two had a history of abortion, one spontaneous and one induced. The time between the first consultation and the completion of treatment ranged from two to 50 months, with a mean of nine months. The mean age of patients who underwent stimulation was 33.9 years. As for treatment, 37 couples had a single IVF cycle, and three had two cycles (one of them for a second child). In four cases, both performed stimulation, but only one transferred. All couples used a semen bank, six participated in a shared egg donation program, and seven underwent PGT-A. Regarding the evolution of the cases, the average number of oocytes per patient was 16.7; inseminated eggs was 9.8; fertilized eggs was 7.6; divided embryos was 7.5, and, finally, the mean number of blastocysts per patient was 5.1. Twenty-two fresh transfers were carried out (1.4 embryos/patient), resulting in nine clinical pregnancies (40.9%). In 13 cases, the embryo generated by the egg of the one who stimulated was transferred, which resulted in 6 pregnancies (46.2%); in nine couples, the embryo was transferred to the other partner’s uterus, which resulted in three pregnancies (33.3%). The demand for IVF by homosexual couples increased 150% from 2019 to 2020, from 6 to 15 patients.

**Conclusion:** The present study showed that in five years, only 44 couples sought care, less than nine couples per year (less than one per month), but with a significant increase in 2020. Most couples were referred by doctors, and the great most patients were from the health area. The age difference does not seem to interfere with the process and may vary considerably. It is important to consider the percentage of pregnancy when the ROPA (reception of oocytes from partner) method was used, which was lower than when the oocyte and uterus were from the same patient.

## P-64. Will science be able to use tubes in uterine transplant: The role of MRI - HSG in preoperative planning of uterine transplantation

**Juliana Sabio Lira^1^, Luiz Fernando Pina Carvalho^2,3^, Leandro Accardo de Mattos^4,5^**

*^1^Universidade Anhembi Morumbi, São Paulo, Brazil*

*^2^Baby Center - Center for Reproductive Medicine, São Paulo, Brazil*

*^3^College - Institute of Clinical Research and Teaching Development, São Paulo, Brazil*

*^4^Alta Excellency Diagnostic/DASA Department of Diagnostic Imaging/DASA, São Paulo Brazil,*

*^5^Department of Diagnostic Imaging, Federal University of São Paulo, São Paulo, Brazil*

**Objective**: The aim of the study is to evaluate the use of resonance hysterography in the preoperative planning of uterine transplantation.

**Methods**: A potential living donor was evaluated by imaging prior to uterine donation for uterus transplantation. The imaging protocol was divided into three phases: in the first phase T1- and T2- weighted sequences were performed for evaluation of pelvic structures with approximately 15 minutes duration. In phase 2 cervical cannulation was performed with a 5F balloon catheter coupled to the specific HSG equipment. About 15 ml of a solution made of gadolinium (Dotarem^®^) and 0.9% saline (1: 100) were administered, 20 acquisitions were obtained to allow detailed assessment of the morphology and contrast flow dynamics through the uterine cavity and tubes, along with peritoneal spillage. Soon after, a VIBE sequence with high spatial resolution was performed- lasting approximately 3 minutes. In phase 3 the patient was encouraged to move freely and, after approximately 15 minutes, returned for the acquisition of a last T1-weighted fat-suppressed sequence (high spatial resolution VIBE, lasting around 30 seconds) to check for contrast dispersion in the peritoneal cavity and retention of residual contrast in the tubes. When there was still contrast in the uterine cavity, we repeated this step after another patient ambulation.Some adjustments of the examination protocol were made throughout the study, especially regarding the dynamic sequence, in order to obtain higher spatial resolution. In order to reduce bowel peristalsis related artifacts, intravenous antispasmodic (1 ml Buscopan^®^ 20mg/ml) was administered in two doses, immediately before the start of the scan and before endocervical contrast injection. Image analysis was performed on a workstation with Picture Archiving and Communication System (Carestream PACS) by a radiologist (LAM) with over 10 years’ experience in gynecological imaging. Imaging scans were interrogated for infertility-related uterine and extrauterine abnormalities

**Results**: The image reveals a uterus in anteversoflexion and permeable cervix of normal caliber. Uterine body cavity with anatomical shape, capacity and contours, with no evidence of filling failures inside. Bilateral permeable fallopian tubes with intact mucosal relief in their various segments, positive Cotte sign bilaterally. Contrast-injected tube with normal peritonization. Withdrawal of the cannula with ambulation for 15 minutes, wide spread of contrast after two deambulation cycles. The size and quality of the uterine venous drainage via uterine or utero-ovarian veins are within the normal range. Absence of abnormalities, malformations, impermeability and extravasation of contrast.

**Conclusion**: The uterus transplant is a complex procedure, performed with the purpose of promoting fertility and consequent pregnancy in the patient, so it combines solid organ transplant techniques and assisted reproduction techniques. It is necessary to have a detailed study of images, so that the possible intraoperative findings could be minimized through a careful analysis of the images of both the donor and the recipient. Most importantly, close coordination between the transplant team and an experienced radiologist was valuable to identify patients with relative or absolute contraindications to transplantation such as significant uterine arterial atherosclerosis, uterine anomalies in the donors, adenomyosis, etc. And also to plan the graft procedures appropriately. Through experience it is possible to perceive that the evaluation of the tubes through the resonance in the process can bring future applicability for the uterus transplant including the structure.

## P-65. Prisoners and reproduction: the restriction of the right to procreate as part of the punishment?

**Rafaela dos Santos Pinto Ferreira^1^, Thiago Delmondes Feitosa^2^, Júlia Garcia Greggio^1^, Mariana Augusto Lisboa^1^, Luiz Fernando Pina de Carvalho^3,4^**

*^1^University Nove de Julho ,São Paulo, Brazil*

*^2^Hospital Maternidade de Vila Nova Cachoeirinha*

*^3^Baby Center , São Paulo, Brazil*

*^4^College - Institute of Clinical Research and Teaching Development, São Paulo, Brazil*

**Objective:** The objective of the study is to collect data on the prison population in Brazil and analyze the length of sentence, number of children, average age, among other topics, in order to correlate with reproductive desire and family constitution.

**Method:** Using the PUBMED platform , a search was performed for articles with the following descriptors: pregnancy and aging; ovarian reserve and age. The inclusion criteria were the English language, in the period between 2010 and 2022 and that were suitable for the discussion of the topic . We also searched for terms that correlated prisoners and pregnancy “prisoners and fertility”, “prison and IVF”, but no articles were found. At the same time, data provided by the National Penitentiary Department from July to December 2021 was analyzed in order to obtain information about inmates in prisons in Brazil . These data exclude prisoners who are in the custody of the Judiciary Police, Police Battalions and Military Firefighters, as well as those in the home regime.

**Results:** From July to December 2021, there were 670,714 inmates in tax cells, of the total, 30 ,625 are female. There was an intense growth of them in prisons, in 2000 there were 6 thousand and in 2020 they exceeded 30 thousand, that means a growth of 500%. Data collected from 968 inmates shows the length of time in prison for these women: 54.11 % spent 4 to 15 years in prison. Detainees who have their data collected on the number of children are only 27.88% of the total. It was described that 47.69% of them did not have children; 24.05% had only 1 child; and only 14.22% had 2 children; the remainder, 14.04%, had 3 children or more. Their ages vary, 59.81% are between 18 and 34 years old; 21.95% are between 35 and 45 years old and only 18.24% are over 60 years old or there is no information about their ages.

**Conclusion:** It is known that the human ovary contains a fixed number of antral follicles established before birth that decline with increasing age, culminating in menopause around the age of 50. Wallace and Kelsey, 2010 estimated that for 95% of women at age 30, only 12% of their maximum population of antral follicles is present and at age 40 only 3% remain. *In vitro* fertilization techniques open up new questions and offer many possibilities for men and women who remain incarcerated for years. It was possible to conclude that 81.76% of the imprisoned women are of childbearing age and many of them spend long periods without freedom, this directly impacts their reproductive capacities, often contributing to the non-constituting of a family. It is questioned whether adequate gynecological and reproductive care is offered in this place and it is suggested that a more focused research on this topic within prisons is essential to investigate the possible deprivation of the right to conception to this marginalized population. The authors propose social preservation of fertility as a method of choice for the incarcerated population.

## P-66. Obstetric and neonatal outcomes between donors and recipients in oocyte donation FET cycles

**Marta Ribeiro Hentschke^1^, Mariangela Badalotti^1^, Natália Fontoura de Vasconcelos^1^, Vanessa Devens Trindade^1^, Victoria Campos Dornelles^1^, Isadora Badalotti-Teloken^1^, Débora Farinati^1^, Alvaro Petracco^1^**

*^1^Fertilitat, Reproductive Medicine Center*

**Objective:** To analyze if there is any difference in obstetric and neonatal outcomes between egg donors and recipients in frozen embryo transfer (FET) oocyte donation cycles.

**Methods:** Retrospective, observational study using data from 247 FET cycles from egg donor program, performed between 2008 and 2021. It includes only frozen cycles due to the possible influence of high estrogen levels in the endometrium, pregnancy, and fetal development in fresh donor cycles. Of the 247 cycles, 143 were from donors and 104 from recipients. The embryos were obtained from fresh eggs using the ICSI technique and vitrified at the blastocyst stage of development. Regarding the endometrial preparation, most were prepared with estradiol valerate (over 90% in both groups). Exclusion criteria included preimplantation embryo biopsy. Statistical analysis was performed using the student t-test and chi-square test, considering p<0.05.

**Results:** The whole clinical pregnancy rate was 40.9% (101/247), resulting in 81 births or ongoing pregnancies (32,8%). When comparing Donors (G1) *versus* Recipients (G2), the following results were found: maternal age (years) (31.3 ± 3.8 *vs*. 44.0 ± 4.9, *p*<0.001); paternal age (years) (35.9 ± 5.7 *vs*. 43.1± 6.4, *p*<0.001); single embryo transferred percentage (83/143, 58.0% *vs*. 58/104, 55.8%, *p*=0.72); pregnancy rate, (72/143, 50.3% *vs*. 50/104, 48.1%, *p*=0.72), clinical pregnancy rate (60/143, 42% *vs*. 41/104, 39.4%, *p*=0.69), live birth rate (per clinal pregnancy) (44/60, 73.3% vs. 25/41, 61.0%, *p*=0.18); miscarriage per clinical pregnancy (9/60, 15% vs. 11/41, 26.8%, *p*=0.14); single fetus rate (37/44, 84.1% vs. 21/25, 84.0%, *p*=0.99); gestational age (GA) at delivery (weeks), (37.7 ± 2.7 vs. 36.8 ± 2.8, *p*=0.22), birth weight (g) (3026.5 ± 723.9 vs. 2838.5 ± 775.8, *p*=0.32), birth length (cm) (47.5 ± 3.30 vs. 46.7 ± 4.0, *p*=0.33), birth percentile (1st newborn), (small for gestational age, SGA, 4.8% vs. 0.0%; adequate for GA, AGA, 73.8% vs. 80.0%; large for GA, LGA, 21.4% vs. 20.0%, *p*=0.55).

**Conclusion:** Recipients presented similar outcomes compared to donors. Even though not statistically significant, recipients showed slightly lower gestational age and birth weight, which could be associated with advanced maternal age. By including only FET cycles, we could minimize the estrogen effect on endometrial receptivity and fetal development in donors.

## P-67. Is embryonic quality limited to compulsory notification rates?

**Rafaela dos Santos Pinto Ferreira^1^, Thiago Delmondes Feitosa^2^, Luiz Fernando Pina de Carvalho^3,4^**

*^1^University Nove de Julho, São Paulo, Brazil*

*^2^Hospital Maternidade de Vila Nova Cachoeirinha*

*^3^Baby Center - Center for Reproductive Medicine - São Paulo, Brazil*

*^4^College - Institute of Clinical Research and Teaching Development, São Paulo, Brazil*

**Objective:** The objective is to analyze the data available in the National Embryo Capture System (SisEmbrio) and compile rates obtained by compulsory notification in the last 10 years, as well as to question the quantitative evaluation in opposition to qualitative data.

**Methods:** A total of 13 published reports in SisEmbrio were analised, started in 2007 and updated annually; following the reports the variables collected in human reproduction treatments with number of oocytes collected after ovarian stimulation; fertilization rate: expressed as the number of oocytes, number of embryos transferred; it also includes freezing rates.

**Results:** It was possible to verify that SisEmbrio is indoctrinated to be limited with quantitative data, brings from the sixth reports the embryonic cleavage rates, *in vitro* fertilization rate (this indicator has behaved uniformly since 2011, with stable values within the range of 73% to 74%.), average oocytes per woman (SisEmbrio data showed that this indicator has behaved uniformly since 2011, stable values within the range of 8.7 to 9.2.); produced from the *in vitro* fertilization cycles performed. In 2019, the region with the lowest mean oocytes per woman was the Southern region (7.5). The one with the highest average was the Midwest (9.4), but this region had the lowest fertilization rate (73%) of the national average (76%).

**Conclusion:** The compulsory notification proposed in the Ministry of Health with information on human reproduction should follow rigorous routines, after all there are methodologies proposed by the Interagency Health Information Network that developed the form of quality indicators in Germ Cell and Tissue Banks. Both quantitative and qualitative indices are evaluated; however, it is observed in the SisEmbrio reports the prevalence of quantitative evaluations, limiting human reproduction to prospective numbers and calculations, is questioned whether such evaluation is really effective, or as efficient, as qualitative parameters to reduce marital infertility.

## P-68. Role of epigenetics in egg donation, what do we already know?

**Gabriela Gouveia^1^, Gabriela Wroblewski^1^, Gabriel Monteiro Pinheiro^1^, Karolyne Vale de Sá^1^, Mariana Kasuga Morya^1^**

*^1^Universidade Santo Amaro*

**Objective:** The objective of the present study was to assess existing scientific evidence of **a** correlation between egg donation and epigenetics.

**Methods:** A systematic literature review was performed by searching PubMed (Medline) and Google Scholar. A total **of** 18 studies between January 2002 and May 2020 were found. However, only 11 studies showed a correlation between egg donation and epigenetics.

**Results:** The egg donation is a reproduction technique on the rise not only in Brazil but all around the world. One of the factors that explains the rising number of egg donation procedures is the superior quality of some of the eggs, which is used for infertility treatments in order to achieve pregnancy. However, many potential recipients end up rejecting the egg donation given that the genetic information between the mother and future baby would be different. The available literature research into epigenetics has demonstrated that the prenatal uterine environment is crucial in fetal brain development and as part of other physiological functions, such as metabolism, body structure, and the immune system. The exposure of preimplantation embryos to environmental influences induce altered fetal development with postnatal and prenatal consequences. Some of the consequences that have been reported include: fetal growth restriction, congenital malformations, imprinting disorders, risk of gamete/embryo diseases originated from donated eggs, such as early-onset diabetes and cardiovascular diseases, mitochondria! heteroplasmy (including associated abnormalities in mitochondria) translation products), altered patterns of X inactivation, and altered levels of histone acetylation. In addition, the production of early stage nuclear transfer embryos derived from egg donation failed to fertilize oocytes, revealing that most embryos are karyotypically abnormal, resulting in developmental arrest. On the other hand, advances in assisted reproduction therapies are decreasing the amount of epigenetic changes in gametes and embryonic stages of egg donation.

**Conclusion:** The scientific evidence showed that some subfertile couples have a genetic predisposition to epigenetic instability, which makes their offspring more susceptible to epigenetic changes. However, advances in assisted reproduction technology are decreasing the amount of epigenetic changes in gametes making them less susceptible to abnormal modifications.

## P-69. Analysis of KIR receptors in repeated implantation failures in Assisted Reproduction cycles

**Vitória Luiza Batalhoti Brogiato^1^, Leticia Vezneyan Povia^1^, Karolyne Vale de Sá^1^, Nesrin Klaled Yassine^1^, Maria Fernanda Marques dos Santos1, Gabriel Monteiro Pinheiro^1^**

*^1^Universidade Santo Amaro.*

**Objective**: To analyze and relate the role of KIR receptors and HLA particles in assisted reproduction treatments and in fertilization failures.

**Methodology:** Bibliographic review based on the selection and analysis of articles published in the last ten years on PubMed, Lilacs and Scielo platforms, using the keywords “assisted reproduction”, “Killer-cell immunoglobulin-like receptors” “HLA-C” “infertility”, in addition to being limited to Portuguese, English and Spanish.

**Results:** After analyzing the characteristics of each of the articles studied on the subject, we see that the role of the immune system, especially the set of Natural Killers (NK) cells, in pregnancy and abortion has been increasingly studied in order to elucidate recurrent failures in fertilization treatments. These cells are important in the body’s defense mechanism and are characterized by the CD56 surface receptor, which have a cytotoxic action and lead to cell destruction. It has been known since the confirmation of the missing-self hypothesis , that the killer immunoglobulin- like receptors (KIR) on NK cells mediate the recognition of the HLA-C antigen present in the embryo to determine the action of NK on this one. It is believed that the gestational product has 50% of the genetic material of paternal origin, and that uterine NK cells bind to HLA-C molecules on placental invading trophoblast cells. Both KIR and HLA-C receptors have subtypes genetically inherited differences, which influence the blockage or activation of the NK cell after contact with the embryo, considering that, the negative and positive influences on the development of pregnancy are analyzed; it is established that women with a history of failure in assisted reproduction treatments have higher levels of NK cells, and the values considered acceptable for implantation vary between studies, so changes in the number and/or activity of these cells may trigger an increase in its cytotoxicity, generating reproductive failures or gestational complications. In a retrospective study including 291 women with Recurrent Implantation Failure who had 1304 IVF cycles, it was found that assisted pregnancies differ from unassisted pregnancies, in that patients receive mainly DTE (Double Embryo Transfer) with more antigens. Non-self HLA presented to the mother’s KIR compared to “normal” pregnancies. Furthermore, donor oocytes are often used in assisted reproduction, and no other report has studied the impact of KIR-HLA-C on donor oocyte cycles. Another study of 668 euploid embryo transfers with stored maternal DNA and available pre-amplification DNA from previous trophectoderm biopsy samples concluded that carriers of KIR A haplotypes experienced less pregnancy loss than carriers of KIR B haplotypes after single-embryo euploid transfer. However, this risk was modified by the HLA-C alleles present in the embryo. High-risk combinations (KIR A/C2 homozygous and KIR B/C1 homozygous) resulted in a 51% increased risk of loss over all other combinations.

**Conclusion**: The findings of this study point to the importance of the specific combination between the receptors for KIR and HLA-C activation, especially in cases of failure in ovoreception, and their maternal-fetal interaction for reproductive success. It is pointed out that in the future the selection of combinations of variants will be useful to reduce the cases of fertilization and embryo implantation failure, thus improving the quality of assisted reproduction treatment. In addition to the transfer of a single embryo, thus decreasing the cytotoxic reaction of NK cells to different types of RA techniques.

## P-70. Progestin-Primed Ovarian Stimulation Protocol for Patients in ART Abstract

**Maria Fernanda Marques dos Santos^1^, Vitória Luiza Batalhoti Brogiato^1^, Beatriz de Moraes Valery^1^, Gabriel Monteiro Pinheiro^1^**

*^1^Universidade Santo Amaro*

**Objective**: This article seeks to analyze the use of an alternative strategy, progestin, in pituitary block, from the perspective of cost, applicability, risks and mainly results when compared to protocols Pituitary block standard in controlled ovarian stimulation (COS).

**Methodology**: Bibliographic review based on the analysis of articles published in the last ten years on PubMed and Scielo platforms, in addition to being limited to the languages: Portuguese, English and Spanish. The research was carried out from the keywords: Therapy with Drugs, Progestins, Progestagens, GnRH, Assisted Reproduction Techniques.

**Results:** Controlled ovarian stimulation (COS) is a key component of assisted reproductive technologies (ART), shifting clinical practice from natural monofollicular cycles to stimulated multiple follicle cycles in IVF treatment. There are different protocols for ovary stimulation in assisted reproduction cycles, these aim to improve oocyte quality and quantity. Gonadotropin- releasing hormone (GnRH) is responsible for stimulating the pituitary gland to release gonadotropin and luteinizing follicle-stimulating hormone necessary for gametogenesis and steroidogenesis; thus, to prevent ovulation and premature luteinizing hormone (LH) increase is essential the administration of GnRH agonists or antagonists , but they have distinct disadvantages such as the increased risk of ovarian hyperstimulation syndrome (OHSS), formation of ovarian cysts, hypoestrogenic symptoms, higher rate of cycle cancellation, in addition, these treatments are expensive and their administration is subcutaneous, being, therefore, often not advantageous for patients. Aiming at this, studies began to be carried out to find other means of suppressing the pituitary gland. Thus, synthetic progestogens, called progestins emerged as alternatives to GnRH analogues, being a drug with progesterone receptor agonist action, which has the function of simulating the effect of this hormone . In a recent study, overall live birth, ongoing and clinical pregnancy rates by embryo transfer were found to be similar with progestins and GnRH analogues. However, when progestins were compared with GnRH agonists, sensitivity analyzes including women with polycystic ovary syndrome and short GnRH agonist protocols showed significantly higher clinical pregnancy rates with progestins, i.e. there are ovarian response, pregnancy and similar embryo euploidy. An article that included 304 patients undergoing ovarian progestin stimulation and 152 patients undergoing a short protocol from April 2014 to July 2019 showed that patients stimulated with progestins had a higher implantation rate (43.4% vs. 31.9% , *p* < 0.05), clinical pregnancy rate (61.8% vs. 47.4%, *p* < 0.05) and live birth rate (48.4 % vs. 36.8%, *p* < 0 .05) when compared to those who underwent stimulation via a short protocol.

**Conclusion**: Ovarian stimulation with progestin proved to be effective in inhibiting spontaneous ovulation without affecting the number of oocytes recovered and the quality of the embryo. The use of progestins allows for better control of LH concentrations, lower costs and easier (oral) administration. Therefore, ovarian stimulation with progestin may be the first choice for fertility preservation, oocyte donation and pre-implantation genetic testing cycles in philanthropic services or services that serve the Unified Health System (SUS). In addition, progestin can be used in cycles of poor responders who would be willing to perform oocyte accumulation without excessive expenditure with the pituitary blockers of Standard protocols.

## P-71. Turner mosaicism and the management of fertility preservation

**Isadora Badalotti-Telöken^1^, Marta Ribeiro Hentschke^1^, Maria Teresa Sanseverino^1^, Victoria Campos Dornelles^1^, Ricardo Azambuja^1^, Alvaro Petracco^1^, Mariangela Badalotti^1^**

*^1^Fertilitat- Centro de Medicina Reprodutiva, Porto Alegre, RS*

**Objective:** To report a case of a 19-year-old patient with Turner mosaicism and the management of fertility preservation.

**Methods:** Case report study that included one patient. Data were collected from electronic records from a prospective database. The study was approved by the ethics and research committee of PUCRS, Porto Alegre.

**Results:** A 19-year-old patient comes for her first gynecological appointment with a history of menarche at age 13 and long menstrual cycles with a heavy flow for 7-8 days. Denies hair growth and acne and has occasional headaches. She had never had sex and expressed a great desire for motherhood. She mentioned using growth hormone after taking a genetic test at 11 years old due to her short stature but doesn’t have the result. She had healthy parents and siblings. Physical examination: height = 1.57 m; weight = 60 kg; some facial and upper limb nevus; normal secondary sex characteristics; no signs of hyperandrogenism or Turnerian stigmata. Karyotype, androgens, AMH and pelvic ultrasound were requested. Karyotype demonstrated 4% Turner mosaicism and chromosome 1 inversion. Pelvic ultrasound with a normal uterus and right ovary, and reduced left ovary. AMH = 1.8 ng/ml. Normal level of androgens. Due to the risk of early ovarian failure - besides the mosaicism, AMH lower than expected for the age and reduction in the volume of the left ovary - and the possibility of discrepancy between blood karyotype and gonadal involvement, cryopreservation of eggs was indicated and performed, with a genetic study of follicular cells fluid. Ovarian stimulation was performed with recombinant FSH and hMG in a GnRH agonist regimen. Twelve follicles were aspirated and ten mature oocytes were cryopreserved. The follicular fluid (FF) was centrifuged, and the cells were sent for karyotyping and FISH analysis. The FF karyotype, in the analysis of 71 metaphases, showed 45,X,inv (1)(p11q12)[3] / 46,XX,inv(1) (p11q12)[68]. In the FISH analysis, 500 nuclei were analyzed, and mosaicism was observed for two cell lines: 52% of the nuclei showed two signals for the X chromosome, Xcen(DXZ1x2), consistent with an XX sex chromosome complement, while 48% of the nuclei showed only one signal for the X chromosome, Xcen(DXZ1x1), consistent with monosomy for the sex chromosome X - .nuc ish Xcen(DXZ1x1)[240]/Xcen(DXZ1x2)[260].

**Conclusion:** Patients with Turner are at high risk for premature ovarian failure, and fertility preservation is recommended in adolescent or young adult patients. Until recently, this group of patients depended on egg donation or adoption to become mothers. Egg freezing has been presented as an excellent option for these patients. Since oocyte depletion can vary after birth, it is difficult to determine how early to perform the procedure. Studies show that the response to ovarian stimulation in adolescents is consistent with that of adults but may be lower compared to girls of the same age. Some authors argue that egg cryopreservation is not necessary if the mosaicism degree is low. However, the mosaicism degree in the gonad may be higher than in peripheral blood, as in the presented case. In case of pregnancy, it is advised that patients undergo IVF with a preimplantation genetic test due to the increased risk of aneuploidy in fetuses. Moreover, it is important to emphasize the increased risk of maternal gestational complications such as hypertension, diabetes, cesarean section due to cephalopelvic disproportion, aortic dissection and death. This case emphasizes the need for fertility preservation counseling as an integral part of these patients’ care, given the risk of early ovarian failure and the possibility of difference between peripheral and gonadal karyotype, as well as advice on pregnancy-related issues.

## P-72. Outcomes in ART according to couples BMI’s categories

**Victoria Campos Dornelles^1^, Isadora Badalotti-Teloken^1^, Marta Ribeiro Hentschke^1^, Natália Fontoura de Vasconcelos^1^, Vanessa Devens Trindade^1^, Ricardo Azambuja^1^, Alvaro Petracco^1^, Mariangela Badalotti^1^**

*^1^Fertilitat - Reproductive Medicine Center*

**Objective**: To analyse if the outcomes of assisted reproduction techniques (ART) are influenced by the couple’s body mass index (BMI).

**Methods**: Retrospective cohort study performed at an assisted reproductive clinic in the period of 2013 to 2020. Data from 399 couples submitted to ART were collected from electronic records. First analysis: Group 1, eutrophic couples (n = 85) vs. Group 2, couples presenting male overweight/obesity (n = 211) vs. Group 3, couples presenting female overweight/obesity (n = 18), vs. Group 4, couples presenting overweight/obesity (n = 85). Second analysis: Group 1, eutrophic men (n = 103) vs. Group 2, overweight and obese men (n = 296). Variables were expressed as n (%). Chi-square and Mann-Whitney U tests were applied, *p* < 0.05.

**Results**: Mean female and male ages were similar between groups (years old),36.0 *vs.* 38.0, *p*>0.05. The following rates were found comparing groups 1, 2, 3 and 4, respectively: fertilization 83.3% vs. 76.9% vs. 85.4% vs. 75%, *p*=0.120; clinical pregnancy 44.6% vs. 37.6% vs. 61.1% vs. 41.2%, *p*=0.522; prematurity 14.8% vs. 23.3% vs. 20% vs. 25%, *p*=0.798, small for gestational age (SGA) 35.7% vs. 9.4% vs. 0% vs. 9.1%, *p*=0.096; adequate for GA (AGA) 64.3% vs. 81.3% vs. 100% vs. 90.0%, *p*=0.289; large for GA (LGA) 0% vs. 9.4% vs. 0% vs. 0%, *p*=0.430. On second analysis, statistically significant differences were found comparing groups 1 vs. 2 on fertilization 83.3% vs. 76.9%, *p*=0.001 and clinical pregnancy rate 47.5% vs. 38.3%, *p*=0.001. Significant linear association (*p*=0.031) between male BMI and neonatal percentile (*p*=0.031) were found (SGA 29.4% vs. 9.3%, p=0.049; AGA 70.6% vs. 83.7%, *p*=0.252; LGA 0% vs. 7%, *p*=0.264).

**Conclusion:** Overweight and obesity on male partners were associated with lower fertilization and clinical pregnancy rates, suggesting an isolated negative impact on ART outcomes. Higher male BMI was associated with higher birth percentiles, independently of maternal BMI. These results reinforce the importance of attending female and male weight during ART.

## P-73. Laparoscopic treatment of cesarean-induced isthmocele with the aid of hysteroscopy - Case Report

**João Paulo de Oliveira Timbó^1^, Renata Bezerra Amorim^1^, Anne Caroline Amorim Oliveira^1^, Sebastião Evangelista Torquato Filho^2^**

*^1^Hospital Cesar Cals*

*^2^Sollirium*

**Objective:** Describe the case report of a 37 year-old-woman with endometriosis and isthmocele, which was repaired with the aid of hysteroscopy during the laparoscopic procedure.

**Methods:** Case report of a private clinic patient on Fortaleza, Brazil, about isthmocele and infertility.

**Results:** Cesarean-induced isthmocele is a pouch defect on the anterior wall of the uterine isthmus located at the site of a previous cesarean delivery scar. The diagnosis is based on transvaginal and hysteroscopy. Isthmocele is a underrecognized cause of menstrual spotting, postmenstrual anormal uterine bleeding, chronic pelvic pain and infertility, mainly secondary infertility. This last one is associated because the persistence of the menstrual blood in the cervix may negatively influence the mucus quality, obstruct sperm transport through the cervical canal, affect sperm quality or eventually interfere with embryo implantation. The surgical treatment in the symptomatic cases can be hysteroscopic or laparoscopic. The decision about it involves the residual myometrial thickness overlying the isthmocele: on cases > or = 2.5-3.5mm, the safest and most effective strategy is hysteroscopic approach. On cases with < 2,5mm, laparoscopic and vaginal surgeries may be the preferred options. The case we are describing is about a 37-year-woman, married, diagnosed with endometriosis leading to infertility and *In Vitro* Fertilization successful ten years ago, leading to cesarian section on 2012. After this pregnancy, she had two ectopic pregnancies, first one leading to a tubal abortion on 2014 and second resolved with laparoscopic bilateral salpingectomy with ressection of endometriosis focus, on 2016. The second embrionary transfer on 2018 was unsuccessful and she was diagnosed with a big isthmocele with 2,6mm margin of myometrial thickness, suggesting surgical treatment before a new transfer. The surgical approach involved laparoscopie repair with hysteroscopic during the procedure, guiding the exact local of the deffect by transillumination of the uterine wall during laparoscopic and checking it with histeroscopy after correction, making sure it was properly corrected.

**Conclusion**: This type of approach is not a routine, and not described yet, but with successful treatment as a minimal invasive strategy to correct isthmocele in this case, showing the importance of the both inside and outside uterus view during the isthmocele correction. Also, we can reiterate the importance of isthmocele correction before a new embryo transfer, to restore the endometrium and lead it to a viable pregnancy.

## P-74. Frozen human semen stored for 24 years resulted in live births

**Thais Tiemi Higa^1^, Brenda Campos Villa^1^, Mariana Moraes Piccolomini^1^, Edson Guimarães Lo Turco^2,3^, Oscar Barbosa Duarte Filho^1,4^, Fernanda Prado Ferreira^3,4^**

*^1^Labforlife*

*^2^UNIFESP*

*^3^Neo Vita*

*^4^Vida Bem Vinda*

**Objective:** The objective of this case report is to show that long-term semen cryostorage does not affect clinical results, and that maintains its ability to result in live birth through ICSI (Intracytoplasmic sperm injection).

**Methods:** In August 1996, a 29-years old man donated his sperm to a Brazilian sperm bank. The semen was frozen using a freezing medium made in-house (yolk buffer medium with 7 % glycerol) in sealed straws. This semen was used for an IVF (*in vitro* fertilization) treatment at LabForLife reproductive laboratory in 2020. The patients who had chosen this donor were a same-sex couple. They contacted the donor sperm bank and according to the donor’s characteristics, they chose his semen. The process started in January 2020, when they decided to start their reproductive journey, more precisely using IVF-with-ROPA (Reception of Oocytes from Partner). By the time, the patient who provided the eggs was 22 years old and the patient who became pregnant was 28 years old. Also, for financial reasons, they had chosen to donate a part of the retrieved mature eggs to make their treatment affordable. The 22-years old patient received controlled ovarian stimulation with a follitropin delta protocol. The ovarian stimulation lasted 11 days until the egg retrieval day. A total of 33 oocytes were recovered, of which 31 were mature. 21 oocytes were donated anonymously, and 10 oocytes were fertilized via ICSI with the sperm donor. The donor semen was thawed at room temperature for at least 10 minutes. The volume of semen was 0.5 ml, and the total motile count post-thawing was 12.4 million. After processing with a density gradient, the total motile count was 3.2 million. All the 10 oocytes that underwent ICSI resulted in normal zygotes. Two top- quality blastocysts were frozen on Day 5, and the other two lower-quality blastocysts were frozen on Day 6. In the meantime, the “recipient” 28-years old partner had prepared her uterus for implantation through an endometrial preparation treatment. And in the following month, in February 2020, the two top-quality Day 5 blastocysts were transferred to her uterus.

**Results:** There was implantation in the first frozen embryo transfer (FET) cycle. Two blastocysts whose quality was B5AA and B5BB were transferred and resulted in dizygotic twins. The only complication during pregnancy was Hyperemesis gravidarum, but with no high repercussion. Thus, in October 2020, two healthy boys were delivered at the gestational age of 37 weeks. The babies weighed 2475 g and 2250 g, their length was, respectively, 45 and 43 cm. They remain healthy and in normal development to date.

**Conclusion:** This report demonstrates that human semen stored for the long term (24 years) is suitable for ICSI, resulting in live births.

## P-75. Serum progesterone level and pregnancy rates in embryo transfer in artificial cycles in a human reproduction center in the south of Brazil

**Sofia Bulla Paviani^1^, Fabio Firmbach Pasqualotto^1^, Eleonora Bedin Pasqualotto^1^**

*^1^Universidade de Caxias do Sul*

**Objective:** The importance of progesterone to improve endometrial receptivity is unquestionable, leading to an adequate transformation in the luteal phase, helping embryo implantation and maintenance of pregnancy. Recent evidence supports the importance of serum progesterone levels prior to embryo transfer as an independent factor for live-born rates, and inadequate levels of progesterone are related to a significantly higher rate of miscarriages and lower live births. The aim of this study is to evaluate serum progesterone (P) dosage the day before frozen-thawed embryo transfer (FET) and fresh embryos in oocyte donation patients and to correlate with rates of positive Beta-HCG and clinical pregnancies comparing groups with serum P <10ng/mL and >10ng/mL in cycles of artificial endometrial preparation.

**Methods:** This is a longitudinal retrospective study that followed embryo transfers from January 1,2021 to August 31,2021 at the Conception Human Reproduction Center in Caxias do Sul. Data from medical records were evaluated and the serum progesterone dosage was performed the day before embryo transfer, at the Conception clinic, between 8:00 - 10:00 am, and all samples were sent to the same laboratory and analyzed by chemiluminescence. Patients who underwent FET and fresh embryo transfer from oocyte donation with artificial endometrial preparation, with stage D3 and D5 embryos, with or without PGT-A were included. Patients with serum P <6ng/mL, endometrial thickness <7mm or uterine abnormalities were excluded. The variables studied were time and cause of infertility, ovarian stimulation (number of developed follicles and oocyte retrieval), embryo transfer, embryo stage and whether or not preimplantation genetic testing for aneuploidies (PGT-A) were performed.

**Results**: A total of 70 transfers met the inclusion criteria. Maternal age, together with couple comorbidities, as well as time of infertility, did not have a statistically significant result. Regarding ovarian stimulation, the median number of follicles developed (22.0 IIQ 17.0 - 32.0) and the median number of oocytes retrieval (15.0 IIQ 12.0-21.0) were higher in patients with clinical pregnancy rates and showed significant p values, respectively *p*<0.01 and *p*=0.04. Embryo transfers at the blastocyst stage also showed statistical significance for the clinical pregnancy outcome, with *p*<0.01, where 17 of the 19 cases of clinical pregnancy were from D5 embryos, and only 2 were from D3 embryos. PGT-A analysis were not correlate with higher chances of pregnancy in our study. Although not significant, in the P>10ng/mL group there was a trend towards a higher rate of positive Beta-HCG (37% vs 31.3% *p=*0.67) and a higher rate of clinical pregnancy (29.6% vs 18.8% *p=*0.39). The ROC curve describes a significant positive predictive value for serum progesterone levels the day before embryo transfer, with AUC = 0.629 (95%CI: 0.47 - 0.78) *p=*0.10. Median P was higher in the group with clinical pregnancy 13.5ng/mL (11.0 - 21.4) vs 11.5ng/mL (10.0 - 14.5) than the group with no pregnancy.

**Conclusions**: In line with recent evidence our study showed that in the P>10ng/mL group there was a trend towards a higher rate of positive Beta-HCG and a higher rate of clinical pregnancy. it is hoped that this study will serve as basis for future research and that it can collaborate with current scientific evidence. Future studies and a large sample are necessary to look for specific cut-off values, ways to increase progesterone levels and to assess whether such increases will result in better pregnancy outcomes.

## P-76. Ethanol sclerotherapy for ovarian endometriomas complicated by spontaneous ovarian abscess

**João Paulo de Oliveira Timbó^1^, Renata Bezerra Amorim^1^, Anne Caroline Amorim Oliveira^1^, Eduardo Lima Rocha^2^**

*^1^Hospital Cesar Cals*

*^2^Hospital Antonio Prudente*

**Objective:** To report a case of Endometrioma complicated with ovarian abscess, expose and discuss the therapeutic methods associated with the purpose of disseminating this rare alteration and establishing the benefit of radiointerventional treatment for the case.

**Methods:** information was obtained through electronic medical records review and literature review.

**Results:** Patient seen in the emergency room with a history of colicky, acyclic pain in the right iliac fossa, beginning approximately one month ago, associated with vaginal discharge and fever. Transvaginal ultrasound demonstrated a 12 cm cystic ground-glass image in the right ovary, signs of endometriosis and posterior cul-de-sac block confirmed by magnetic resonance. Two procedures were performed with endometrioma alcoholization using 95% Ethanol, intravenous antibiotic therapy and maintenance dienogest 2mg/day. The evolution was satisfactory, without fever or discharge with significant improvement in pain. The patient is under ultrasound follow- up for identification of recurrence.

**Conclusion:** ovarian cystectomy may lead to a decreased ovarian reserve owing to removal of adjacent healthy ovarian tissue and/or excessive electrocoagulation of the ovary for hemostasis. These findings have led to a more conservative treatment approach. An alternative technique to spare the ovarian reserve is chemical ablation with ethanol sclerotherapy. Literature data have demonstrated the benefit of ethanol therapy in order to preserve an ovarian reserve in patients who may still get pregnant.

## P-77. Intracytoplasmic sperm injection into 306 in-vitro matured oocytes: fertilization and embryo quality

**Camila Dutra de Souza Francisquini^1^, Vinicius Bonato da Rosa^2^, Samara Artuso Giacomin^1^, Alessandro Schuffner^1^**

*^1^Conceber Centro de Medicina Reprodutiva*

*^2^Brown Fertility - Florida Fertility Clinics*

**Objective:**The objective of the present study was to evaluate fertilization and embryo quality after intracytoplasmic sperm injection (ICSI) of *in vitro* matured oocytes (IVM).

**Methods:**In this retrospective observational study, data were compiled on IVM oocytes from patients undergoing *in vitro* fertilization (IVF) between 2014 and 2020. As inclusion criteria, we used patients aged between 24 and 45 years, regardless of couples infertility factor, with oocytes retrieved by follicular aspiration 35 hours after the trigger (hCG). After 3 hours of follicular aspiration, the oocytes were denuded and classified as to their maturity. Those oocytes that were immature, that is, in stage of metaphase I (MI) and with the presence of the germinal vesicle (GV) were kept in culture in the incubator and re-evaluated the following day (about 24 hours after follicular aspiration). Those oocytes that were in metaphase II stage, that is, *in vitro* matured, were injected with fresh ejaculated semen. The IVM oocytes (n=306) were morphologically classified and divided into 2 groups: Normal IVM Group (n=183 oocytes) and Altered IVM Group (n=123 oocytes). After 16 to 20 hours of ICSI, the fertilization of the oocytes was evaluated and the parameters were compared between the groups: Normal fertilization - presence of 2 pronuclei (PN) in the cytoplasm of the oocyte and presence of 2 polar bodies (PC) in the perivitelline space of the oocyte (%), abnormal fertilization - 1PN, 3PN or 4PN (%) and post-ICSI degeneration (%). For those fertilized correctly, embryo quality was monitored until day 3 of development, and the parameters were compared between groups: Embryos discarded due to developmental arrest or degeneration (%), viable embryos eligible for transfer or cryopreservation (%) and good embryos on day 3 of development (%). The good embryos at day 3 were those with 7 to 10 cells, 0% of fragmentation, symmetry and normal blastomere morphology. Embryos from IVM oocytes that were transferred before day 3 of development were excluded from the study.

**Results:** Mean for age of patients was compared by statistical test for analysis of variance (one-way ANOVA) with Tukey’s test and did not differ between groups (36.0±4.0 and 36.8±4.1, *p*>0.05) and rates were compared by the Z-test statistical method for 2 proportions, adopting the statistical significant *p*<0.05. The normal fertilization rate for the normal IVM oocytes group was 56% while for the altered IVM oocytes group it was 42% (*p*<0.05). The normal IVM group had a 7% abnormal fertilization rate, which was statistically lower (*p*<0.05) than the altered IVM group with 16% abnormal fertilization. The post-ICSI degenerated rate was statistically higher (*p*<0.05) in the altered IVM group (22%) compared to the normal IVM group (4%). For those fertilized correctly, in both groups, they had the same rate of non-viable embryos that were discarded (75% for both; *p*>0.05). The same occurred for the rate of viable embryos, where in both groups it had a rate of 32%, not being significant (*p*>0.05). For the rate of good embryos, there was no difference between the groups (*p*>0.05) with normal IVM (30%) and altered IVM (27%).

**Conclusion:** In absolute numbers IVM ICSI produced only 9 good embryos at day 3 of development (6 from normal IVM oocytes and 3 from altered IVM oocytes) out of a total of 306 IVM oocytes injected, demonstrating that IVM ICSI does not result in an outcome expressive for IVF patients. And that despite a higher fertilization rate in normal IVM oocytes, embryonic development and their quality does not differ according to IVM oocyte morphology.

## P-78. Analysis of the repercussions, in a public service of assisted reproduction, of the limitation imposed by Resolution CFM 2294/21 regarding the number of embryos generated in IVF laboratory

**Yamara Alves de Macedo^1^, Victor Edgard Tavares Sousa^1^, Ingrid de Oliveira e Silva^1^, Natalia Ivet Zavattiero Tierno^1^, Mariana Fonseca Roller^1^**

*^1^Hospital Materno Infantil de Brasília*

**Objective**: The present study aims to analyze the impacts, in a public service of assisted reproduction, of the limitation imposed by Resolution CFM 2294/21 regarding the number of embryos generated in IVF laboratory since its publication on 2021/05/27.

**Methods**: Retrospective study, including all fresh cycles of *in vitro* fertilization (IVF) performed at the Assisted Reproduction Center of the Hospital Materno Infantil de Brasília (CEPRA/HMIB), occurred between 2021/05/28 and 2022/07/07. Were assessed the number of oocytes collected, mature oocytes (MII) inseminated, normal mature oocytes discarded (dMII), fertilization rate, number of embryo vitrification procedures, number of embryo transfers (ET), number of cryopreserved and transferred embryos, factor of infertility and age of the patients.

**Results:** 114 IVF cycles were performed, of which 105 resulted in at least one MII retrieved. Among the 860 oocytes collected, 609 were mature. Of these, 555 were inseminated and 134 viable embryos were vitrified, making up 50 cryopreservation procedures; 49 fresh ET were performed, with 84 embryos transferred. The fertilization rate was 64.5%. Patients had a mean age of 37.0 ± 3.3 years. The proportion of cycles that presented above eight MII captured was 21.9%, causing the discard of 54 normal MII oocytes, aiming to limit the production of embryos in the laboratory. The patients had a mean age of 36.9±2.5 years among the 17 cycles in which normal MII was discarded. 14 cycles had an indication for freeze-all due to clinical impediment to fresh ET, and in only one there was cryopreservation of ET surplus embryos. Two procedures did not result in fresh ET or cryopreservation due to lack of viable embryos.

**Conclusion:** The imposed changes in the ethical norms for assisted reproduction procedures interfered with the center’s protocols, limiting clinical-laboratory practice by disregarding infertility factors and patient’s age, in addition to affecting the couple’s autonomy in deciding on their own family planning . Under Resolution CFM 2294/21, following the guidelines of the Brazilian Society of Assisted Reproduction (SBRA), CEPRA/HMIB began to limit the amount of mature inseminated oocytes, discarding those that were in excess, so as not to generate more than eight embryos in IVF laboratory. As a result, 22% of IVF cycles were impacted in some way. The frequency of embryo cryopreservation procedures was not affected by the current standard, but when extrapolating the rates of viable embryo formation, the dMII would present considerable probabilities for the formation of viable embryos. Considering indirect effects, not yet observed, 14% of procedures performed with total cryopreservation may not have euploid embryos due to the limited amount of injected oocytes, maternal age and infertility factor, as widely demonstrated in the literature. Furthermore, patients with a poor prognosis, a history of low fertilization rates, advanced age and without children, are subjected to the same limits as those with an excellent prognosis, with previous pregnancies and successful cycles. Such facts can compromise the pregnancy results, implying additional costs, procedures and risks. Is it fair to sacrifice patients’ chances of success, considering an emotionally and financially costly treatment, in order to limit the number of embryos generated in the IVF laboratory?

## P-79. Incidence of thrombophilia in IVF patients with history of recurrent abortion and/or implantation failure

**Edilberto de Araújo Filho^1^, Leonardo Previato de Araújo^1^, Cássio Leão Fácio^1^, Ligiane Alves Machado-Paula^1^, Ligia Previato^1^**

*^1^Centro de Reprodução Humana de São José do Rio Preto (CRH Rio Preto), São José do Rio Preto, SP, Brasil*

**Objectives:** Evaluate the incidence of thrombophilia (TB) in patients undergoing IVF with history of recurrent implantation failure and/or abortion, because complications due to antiphospholipid antibodies (AFA) are well known such as: recurrent abortions, pre-eclampsia, intrauterine growth retardation, abruptio placenta. Our secondary objective was to evaluate the incidence of TB in the different causes of infertility.

**Methods:** Retrospective observational study from Jan/2018 to May/2022. Age of the patients varied from 27 to 40 years. A total of 74 patients were included in the study. Inclusion criteria: ≥ 2 history of clinical abortion and/or ≥ 2 implantation failure (≥ 2 tranfers of good quality blastocysts (≥ 3 blastocysts)) in cycles of fresh and/or frozen embryo transfers. Causes of infertility included in the study: endometriosis, tubal factor, male factor, unexplained infertility, polycystic ovary syndrome (PCOS), low ovarian reserve. Blood was collected of all patients to test for: anticardiolipin antibody (ACL); antiphosphatidyl serine antibody (AFS); antiphosphatidyl ethanolamine (AFE); FAN; Lupic anticoagulant (AL); anti TPO; antithyroglobulin; metilenotetrahydrofolate reductase mutation (MTHFR); mutation in Factor II and V, protein S and C; antithrombin III; Beta 2 glycoprotein I.

**Results:** Forty nine out of 73 patients (67.1%) had high levels (>20.0ng/ml) of AFS/AFE; 7 out of 73 (9.6%) had high levels of ACL (>20.0ng/ml), 4/73 (5.5%) positive FAN (>1/160); 4/73 (5.5%) had anti TPO/thyroglobulin elevated; we had no case of positive lupus (AL); 30/73 patients (41%) had MTHFR heterozygous mutation; 12.3% homozygous mutation for MTHFR; 2/73 (2.7%) heterozygous mutation for Factor V; none for Factor II; no cases for protein C; 2 cases (2.7%) of low protein S (after two dosages) and no case of Beta 2 glycoprotein I or antithrombin III. Twenty four of 27 (89%) of patients with severe endometriosis had elevated (>20.0ng/ml) AFS/AFE; 3 out of 27 (11%) increased ACL (> 20.0 ng/ml); 3/27 homozygous mutation; 1/27 mutation for Factor V; 6/27 (22,2%) heterozygous mutation on MTHFR. Eight out of 11 (72.2%) of patients with tubal factor had elevated levels of AFS/AFE; no cases of ACL, lupus, mutation of factor II, V, protein S and C, antithrombin or anti TPO. Two cases of heterozygous mutation on MTHFR. Eleven out of 15 (73.3%) of patients with unexplained infertility had elevated AFS/AFE; 2/15 (13.3%) of ACL; 2 (13.3%) positive FAN; one case (6.6%) of heterozygous mutation on Factor V and one (6.6%) of homozygous mutation on MTHFR and 8/15 (53.3%) heterozygous mutation on MTHFR. Five out of 9 (55,5%) of patients with severe male factor had elevated AFS/AFE, one case (11%) of positive FAN and 2 cases (22%) of heterozygous MTHFR. Two cases out of 5 with PCOS patients (40%) had elevated AFS/AFE and no cases of other thrombophilia and 2 out of 5 (40%) patients with low ovarian reserve had elevated AFS/AFE.

**Conclusions:** The high incidence of elevated antiphospholipid antibodies in patients with recurrent implantation failure and/or recurrent pregnancy loss abortion may justify its investigation and treatment prior new IVF attempts. Others antiphospholipid antibodies than only anticardiolipin should be analyzed to better evaluate thrombophilia. High incidence of antiphospholipid antibodies in patients with endometriosis, unexplained infertility, tubal fator (DIP) as observed in the literature.

## P-80. Main infertility factors associated with the arrest of embryonic development in patients undergoing IVF cycles

**Atila Sena Almeida^1^, Daniele Pinheiro Freitas^1^, Fabio Vieira Vilela^1^, Joao Pedro Alves de Freitas^1^, Fernanda Cristina Souza Batista^1^, Erica Suzanne Soares Leal^1^, Valmira Bispo de Oliveira^1^, Rafaela Guimarães Diniz Cunha^1^, Genevieve Marina Coelho^1^**

*^1^IVI Salvador*

**Objective:** To investigate the infertility factors associated with the arrest of embryonic development in patients undergoing IVF cycles.

**Methods:** Retrospective cohort study in which a total of 121 patients undergoing *in vitro* fertilization (IVF) cycles from January to December 2019 at IVI Salvador were analyzed . Data such as causes of infertility [advanced maternal age, low ovarian reserve, male factor, endometriosis and others (tubal factor, polycystic ovary syndrome )], seminal and oocyte quality and the types of assisted reproduction techniques that were present in the cases of embryonic arrest : intracytoplasmic sperm injection (ICSI), preimplantation genetic diagnosis (PGT) or egg donation. Patients who did not achieve an embryonic block outcome were excluded.

**Results**: The mean age of the patients was 38.5 ± 4.7. Regarding the type of infertility, it was observed that 55% of patients who had embryonic blockage as an outcome had maternal age over 37 years, 27% with male factor, 7% with low ovarian reserve, 6% with endometriosis and 5% with other causes (tubal factor, polycystic ovary syndrome ). The analysis of seminal quality showed that more than half of the patients (58%) had abnormal morphology, 30% regular and 15 % normal. The influence of oocyte quality on embryonic development was also investigated, where it was found that 57 % of the patients had oocytes considered of low morphological quality (above 3 cytoplasmic alterations), 36% regular (from 2 to 3 alterations ) and 7% normal (maximum of 1 change ). By observing the type of procedure indicated for these patients, it was found that 51% were cases with indication of preimplantation genetic diagnosis (PGT), 44 % only ICSI and 5% egg donation program.

**Conclusion:** Through the analysis of the data obtained, it was noted that more than half of the IVF cases that presented as an outcome the arrest of embryonic development were linked to advanced maternal age and male factor , demonstrating how these factors influence the embryonic quality from birth. fertilization until its development to the blastocyst stage. It is important to note that the oocyte and seminal qualities interfered in the embryonic blockade, since patients who had gametes with morphological alterations presented compromised embryonic development and, in most cases, resulting in the arrest of their embryos. However, further studies that present a greater total number of patients are necessary for a better explanation of the real influence of these factors on the evolution of embryos , since the arrest of the embryo ‘s development is often related to a multifactorial system.

## P-81. The use of Oxitocin-Ornithine-Acid (Atosiban) preembryo transfer in cases of repeated implantation failure (RIF) in IVF

**Edilberto de Araújo Filho^1^, Leonardo Previato de Araújo^1^, Cássio Leão Fácio^1^, Ligiane Alves Machado-Paula^1^, Ligia Previato^1^**

*^1^Centro de Reprodução Humana de São José do Rio Preto (CRH Rio Preto), São José do Rio Preto, SP, Brasil*

**Objective:** Atosiban (AT) is a modified oxytocin that inhibits uterine contration, acting as tocolytic agent. The objective of this study was to evaluate if the use of this drug one hour before embryo transfer increases pregnancy rate in patients with ≥ 2 implantation failure.

**Methods:** Prospective study with 40 patients from January 2019 to March 2022 submitted to IVF cycles of fresh and frozen embryo transfer. Protocol of ovarian stimulation with Recombinant FSH (150UI) from day 2 to 6 + human menopausal gonadotropin (HMG) (150 to 300IU) from day 2 to 6. Ultrasound was made on day 2 of the cycle and then on day 7 and after that was individualized as well as the HMG dose. HCG recombinant or GnRh agonist was administered when ≥2folicles reached 20mm of diameter. Oocyte aspiration guided by ultrasound was performed 36h after. Embryos were all taken to blastocyst stage and 1 or 2 embryos transferred fresh or frozen/thaw. In the frozen/thaw transfer cycles the endometrium preparation was performed with Valerate estradiol 2mg every 8h from day 2 on until the endometrium reached a minimum of 8mm at transvaginal ultrasound, when natural progesterone (200mg) was started at the dosage of 400mg every 12h and when 120h of progesterone administration was completed, the embryo transferred was done. Clinical pregnancy: gestational sac + embryo heartbeat. The particularity of the study is that we selected only patients where we observed endometrial waves during ultrasound all made by the same ultrasonographist. All patients had previous history of ≥2IVF failures with good quality blastocysts (AA, AB, BB, BA), at least 3 embryos transferred in 2 attempts. We used the patient as her own control comparing one fresh or frozen/thaw cycle without AT with a cycle in which the used endovenous A 1 hour before embryo transfer. Statistical analysis was performed using *Chi*-square to compare the two groups in the study.

**Results:** Age of the patients ranged from 27 to 41 years and the causes of infertility were endometriosis, unexplained, tubal factor, severe male factor, habitual abortion (≥ 2 losses). Ten of the 40 patients of the study underwent PGT-A and nine had euploid embryos to transfer and six got pregnant in the first single embryo transfer without using AT (66.7% pregnancy rate). Another 10 patients of the study had all the embryos frozen in the stimulated cycle due to risk of severe ovarian hyperstimulation syndrome (OHSS). Seven got pregnant in the first frozen/thaw embryo transfer. So, from the 40 patients of the study, 13 got pregnant in the first IVF transfer without AT. Twenty-six patients completed the study as designed and one did not come back for transfer. Of the 26 patients that first transfer without AT, six got pregnant and 1 had an abortion and 5 group had their babies and come back for transfer with AT and 3/5 got pregnant again. So, to compare pregnancy rate between the two groups, we have to consider in the NON-AT group the seven patients that froze their embryos for risk of OHSS and the 26 patients that completed all the study (33 patients). Therefore, in the NON-AT group, 13 patients got pregnant out of 33 (clinical pregnancy rate: 39.4%) and one had an abortion in the AT group, of the 26 patients that had embryo transfer 15 were clinically pregnant (PR: 57.7%). The difference between the two groups is not statistically significant (*p*<0.05).

**Conclusions:** In our study we found no statistically difference in terms of pregnancy rate or abortion rate between the two groups, but the number of cases in the study is not powered.

## P-82. Does stress levels influence on the IVF results?

**Lister de Lima Salgueiro^1^, Ellen Cortês Correa^1^, Bernardo Lamounier de Moura^1^, Suelen Patrícia de Lima Pinheiro^1^**

*^1^Clínica Fértilis*

**Objective**: The study was performed, to evaluate if the level of stress/anxiety, affects the pregnancy rate in women submitted to IVF.

**Methods**: In a retrospective study, we analyzed the IVF results in 76 patients classified according to their stress level. Before the treatment, the patients were interviewed by our clinical psychologist, and classified according to their stress levels (Moderated, High and Exacerbated). The evaluation included questions about self-perception, reality perception, professional production, social and affective relationship, defenses and characteristic of psychological symptoms. The pregnancy rate was compared according to the levels. The data were analyzed using Qui square Test for categories variable and “*T Student Test” for numerical variables*.

**Results:** The results showed that in 38 (50%) of all the cases the stress/anxiety was elevated. The pregnancy rate in patients with moderated stress was 65% (50 cases). In the groups with high and Exacerbated stress the pregnancy rate was 36% (28 cases) and 44% (33 cases) respectively.

**Conclusions:** As others studies shows this study suggests also suggests that stress and anxiety in high and exacerbated levels could affect the IVF results.

## P-83. Uterine arteriovenous malformation in a patient undergoing assisted reproduction treatment: a case report

**Fernanda Polisseni Souza^1^, Iara Carlin Torres^1^, Luisa Antunes Queiroz Guarçoni de Almeida^2^, Matheus Monteiro de Oliveira^2^, Marcella Barroso Marques Martins^2^, Luana Werneck Rodrigues de Melo^2^**

*^1^Nidus Medicina Reprodutiva*

*^2^UFJF*

**Objective:** To report the case of a patient with secondary infertility undergoing assisted reproduction treatment, who developed an uterine arteriovenous malformation (UAVM) after a complete spontaneous abortion without curettage or other intrauterine manipulation. The patient was treated by selective arterial embolization of the UAVM, with her reproductive capacity preserved. She is now 15 weeks pregnant. Because UAVM is a rare condition, this case report can potentially contribute to the available literature on therapeutic options and outcomes observed in patients who wish to preserve fertility.

**Methods:** For the elaboration of the case report, data contained in the electronic medical record and the patient’s discharge form were used. Laboratory and imaging tests with their respective reports were also used to illustrate the case evolution. In order to compare the reported case with the available literature, a bibliographic research on the topic was carried out in the PubMed database platform.

**Results:** Based on the clinical picture of prolonged and intermittent genital bleeding, after complete resolution of the miscarriage, the diagnosis of UAVM was made by Transvaginal Doppler ultrasound and confirmed by magnetic resonance imaging of the pelvis. The procedure of choice for the treatment of UAVM was selective arterial embolization, and its success was verified by the same imaging tests used for the diagnosis. Seven months after treatment, the patient underwent transfer of two frozen blastocysts from *in vitro* fertilization performed in 2020. The procedure was successful and the patient currently presents with a singleton pregnancy of 15 weeks and satisfactory evolution.

**Conclusion:** The approach proposed for this case was successful in the treatment of UAVM in addition to maintaining the patient’s fertility, with subsequent pregnancy. More studies are needed in order to establish the most effective diagnosis and treatment methods, especially in patients who have the desire to conceive.

## P-84. Nutritional therapy leading to an increase in anti-Mullerian hormone levels: a case report

**Yohanne Almeida^1^, Evangelista Torquato^1^, Raquel Mattos^1^, Eduardo Miranda^1^**

*^1^Sollirium group.*

**Objective:** The aim of this report was to address the potential positive impact of nutritional therapy on anti-Mullerian hormone (AMH) values.

**Methods**: Case report

**Results:** This is a case report of a female patient, 36 years old, white, weight 51.2kg, height 164cm, a medical radiologist who sought care for infertility workup after 12 months of unprotected attempts. Data were collected according to the determinations for research with human beings (protocol number: 5,245,491), upon patient consent and without conflicts of interest. The patient had previous diagnoses of irritable bowel syndrome and lactose intolerance. She complained of aerophagia and had history of gastritis, frequent diarrheic episodes, abdominal distension, excessive flatulence and hemorrhoids. In addition, the patient reported bruxism, muscle tension, joint pain and difficulty gaining weight. Full laboratorial panel revealed: AMH 0.44ng/mL, vitamin D 37.6ng/dL and ferritin 28.7ng/dL, without signs of reactive autoimmunity, and normal thyroid and gonadal hormonal levels. Through pelvic mapping she was diagnosed with mild endometriosis. Patient was treated with normocaloric, slightly hyperproteic, normolipidic and normoglycemic nutritional therapy with a personalized supply of vitamins and minerals, whose main metabolic targets were intestinal repair and inflammatory control. On her latest follow-up visit after 12 months of therapy, the patient had no intestinal complaints and her new lab values were: AMH 1.1ng/mL, vitamin D 49.8ng/dL and ferritin 35.26ng/dL.

**Conclusion**: It is a fact that both inflammatory bowel diseases and endometriosis are pathologies associated with weaknesses in the intestinal barrier, sharing metabolic and immunological triggers that hinder digestive and absorptive processes. These conditions associated with low food quality and highly stressful environments might trigger metabolic changes that interfere with the reproductive system. Therefore, poor diet is an important factor for failures in pelvic metabolism and, consequently, in the development of diseases. Malnutrition together with maternal advanced age of couples trying to conceive might lead to constant deficits of nutrients to the ovarian follicles, which results in low AMH values and worse follicular quality. Although AMH levels may fluctuate, several studies have already demonstrated an association of low AMH values and poor lifestyle habits. However, the exact magnitude of this influence remains controversial. As nutritional therapy was the only modified variable in the life of this patient between the AMH dosages, the present case report suggests that strengthening of the intestinal barrier, digestive and absorptive improvement, as well as an adequate dietary supply to the body may have had a positive impact on the improvement of AMH levels. Although it is still not possible to identify a “fertility diet”, there are several indications that adequate nutritional therapy can be a positive factor for a better AMH response and, consequently, a woman’s fertility.

## P-85. Progestin-Primed Ovarian Stimulation (PPOS) compared to Gonadotropin Releasing Hormone (GnRH) Antagonist Protocol when Corifollitropin alfa is used for ovarian stimulation. A retrospective study

**Ricardo Mello Marinho ^1,2^, Luciana Campomizzi Calazans^1^, Ana Luisa Menezes Campos^1^, Ana Luiza Prates Campos^2^, Adhara de Queiroz Muradas^2^, Erica Becker Sousa Xavier^1^, João Pedro Junqueira Caetano^1^**

*^1^Clínica Huntington Belo Horizonte*

*^2^Faculdade de Ciências Médicas de Minas Gerais*

**Objective:** In the current stage of ART development, in which embryo freezing is an increasingly common practiceand provides good results, PPOS is a new option for controlling the LH peak, showing comparable results to the GnRH Antagonist Protocol, with more flexibility and lower cost. Although there are many papers published on this topic, there is few data regarding its use with corifollitropin alfa, a long-acting recombinant human FSH (rFSH). Because of a higher half- life, one single subcutaneous injection can replace the first 7 daily injections of gonadotropins, a protocol that can make the treatment even more patient-friendly.

This study aims to find out if ovulation blockade with dydrogesterone in corifollitropin alfa stimulated cycles for Assisted Reproduction Techniques (ART) show similar results to GnRH antagonist blockade.

**Methods:** We obtained data from 267 IVF/ICSI and 54 for egg freezing cycles at Clínica Huntington Belo Horizonte, Minas Gerais, Brazil. All cycles were evaluated for the number of mature oocytes obtained, while the number of fertilized eggs, cleaved embryos in day three and blastocysts were obtained in the IVF cycles. Exclusion criteria were women older than 42 years. The primary outcome was the number of mature oocytes retrieved. The secondary outcomes were duration of stimulation, number of fertilized oocytes and blastocysts obtained in the IVF cycles. Another end point was the occurrence of premature ovulation. The comparison of the variables between the protocols were performed through the Mann-Whitney test. The analyses were performed in software R version 4.0.3 and was considered significance level of 5%.

**Results:** Our populations had a mean age of 37.6±3.7 years with no statistical difference between the two groups. The body mass index (BMI) and the number of antral follicles were also similar.

The duration of stimulation was 11.1 days in the progestin group and 10.3 days in the antagonist group. Our results also showed that a 6.5±4.8 and 7.3±4.7 (*p* = 0.488) MII oocytes and 2.8 ± 2,6 and 2.5 ± 2,5 (*p*=0.560) blastocysts were obtained in the progestin and antagonist groups, respectively. There was no significant difference in any of these endpoints. Premature ovulation did not occur in any cycle.

**Conclusion:** We found comparable number of mature oocytes (MII) and blastocysts in PPOS compared to Antagonist Protocol when corifollitropin alfa was used for ovarian stimulation.

One limitation of our study was the small size of the Antagonist group, explained by the fact that we have been using PPOS as a first option recently. Also, we used laboratory outcomes instead of clinical findings since in many cases the embryos obtained have not yet been transferred. The use of progestins to block the LH peak has the advantages of ease of oral administration and lower cost compared to GnRH antagonists. When associated with a long-acting gonadotrofin, less injections are required, making treatment more comfortable for the patient with no negative impact on the results

## P-86. Characterising Direct Unequal Cleavage using CHLOE-EQ

**Daniela Paes de Almeida Ferreira Braga^1,2^, Amanda Souza Setti^1,2^, Livia Vingris^1^, Patricia Guilherme^1^, Edson Lo Turco^3^, Tayara Vergueiro^3^, Alexa Zepeda^4^, Adriana Brualla^4^, Edson Borges Jr.^1,2^, Cristina Hickman^4^**

*^1^Fertility Medical Group*

*^2^Instituto Sapientiae*

*^3^Embriológica*

*^4^Fairtility*

**Objective**: Direct Unequal Cleavage (DUCs) has been associated with reduced embryo viability in terms of blastulation, ploidy and implantation. The objective of this study was to assess whether DUCs are associated with oocyte quality, whether DUCs have an impact on multinucleation or blastocyst quality.

**Methods**: Retrospective assessment of time-lapse data from a single clinic using manual annotation of oocyte quality and automatic detection of DUC using CHLOE-EQ (Fairtility, Israel), an artificial intelligence (AI) based assistant that supports embryologists with embryo assessment. Categorical data was assessed using Chi-square test.

**Results**: DUCs had a compromised ability to blastulate compared to non-DUCs [DUC: 68% (21/31) vs non-DUCs: 94% (289/307), *p*<0.001]. DUCs were not associated with the presence of a smooth endoplasmic reticulum (SER) in the oocyte [6% (6/98) of DUCs were derived from SER oocytes vs 5% (20/388) of non-DUCs, NS]. DUCs were not associated with whether the oocyte was dark, granular, homogeneous, had an inclusion or was normal (NS). All DUCs had thick zona pellucida (9/9) compared to 68% of non-DUCs (15/22, *p*=0.054). DUCs were more likely to have a non-uniform zona pellucida compared to non-DUCs [DUCs: 7% (6/82) vs non-DUCs: 18% (66/366), *p*=0.01]. DUCs were 4-fold less likely to be multinucleated at the 2-cell stage than non-DUCs [DUC: 7% (2/29) vs non- DUCs: 30% (91/305), *p*=0.03]. DUCs were 7-fold more likely to be multinucleated at the 4-cell stage than non-DUCs [DUC: 7% (2/29) vs non-DUCs: 1% (3/302), *p*=0.06], although this difference did not reach significance. DUCs are more likely to have C-grade quality trophectoderm at the blastocyst stage than non-DUCs [DUC: 53% (10/19) vs non-DUCs 26% (75/285), *p*<0.05]; although the ICM quality was unaffected [DUC: 16% (3/19) vs non-DUCs 14% (39/285), NS]. Patient age was not associated with DUCs (*p*=0.4).

**Conclusions**: Given the growing evidence that DUCs have compromised viability, it is important to understand the biology in how DUCs impact embryo selection. The ability to use AI to detect DUCs to avoid such important information being missed during embryo selection can assist embryologists in maximising their efficacy of embryo selection.

## P-87. The ratio of Anti-Mullerian Hormone per Antral Follicle does not discriminate ovarian responsiveness to gonadotropins for IVF

**Rita Chapon^1^, Vanessa Genro^1^, Rafaela Donato^1^, Camila Bessow^1^, Tatiane Souza^1^, João Sabino L.Cunha Filho^1^**

*^1^Insemine*

**Objective:** The main objective of this research is to investigate if the ratio of anti- Mullerian Hormone per antral follicle is a good marker for Antral Follicle responsiveness to exogenous gonadotropins for IVF.

**Methods:** We prospectively studied 310 IVF -ET (first IVF-embryo transfer, tubal or masculine etiologies) candidates, 24 - 45 years of age, during 2020-21. All of the studied patients met the following inclusion criteria: (i) both ovaries present, with no morphological abnormalities (such as cysts, endometriomas, etc.), no history of past surgery and adequately visualised in transvaginal ultrasound scans; (ii) regular menstrual cycle lengths ranging between 25 and 35 days; (iii) no current or past diseases affecting ovaries or gonadotrophin or sex steroid secretion, clearance or excretion; (iv) no clinical signs of hyperandrogenism; (v) BMI ranging from 16 to 30 kg/m2 . Serum AMH levels were determined using a ‘second generation’ enzyme- linked immunosorbent assay. AFC was counted at baseline; ovarian ultrasound scans were performed using a 5.0- 9.0 MHz multi-frequency transvaginal probe. Recombinant FSH therapy was then initiated, at an individual dosage according to the physician’s option and continued until the day of hCG administration. From the 6th day of recombinant FSH therapy onwards, daily FSH doses were adjusted according to the number of growing follicles. Oocyte retrieval was performed under sedation and embryologists evaluated the number of mature oocytes in the lab. Our data were analyzed using a non-parametric correlation (Spearman) and multivariable analysis (Linear regression). Furthermore, we compared both groups to the ovarian response (poor ovarian response: number of mature oocytes < 4 or normal response: number of collected oocytes > 3) using t student test and multivariable analysis. P was considered significant when < 5%.

**Results:**The mean age of the patients was 35 years (23-45), with a mean and standard deviation of 10±5 for AFC. The mean of AMH was 3 ng/ml (0.05-10.00) and the mean of the ratio of serum AMH per antral follicles was 0.29 (0.01-0.97). Moreover, the mean number of mature oocytes was 6 (0-38). All correlations (Spearman’s rho) between the number of mature oocytes with age (-0.262), AFC (0.533), AMH (0.423) and AMH per AFC (0.179) were significant (*p*<0.001). However, in a linear regression model, only AFC became significant. In addition, grouping patients into poor responders (< 4 mature oocytes, n=95) and comparing them with those with more than 3 mature oocytes; age, AFC and AMH were prognostic factors (*p*<0.001), but the ratio of AMH per AFC did not discriminate poor ovarian response to exogenous gonadotropin (*p*=0.390).

**Conclusions:**AMH per AFC is stable during the spam reproductive life and was not a good marker for ovarian response to exogenous gonadotropins. Age, AMH and AFC are better and could be used to individualize controlled ovarian stimulation and discriminate poor responders.

## P-88. Woman´s Couple and Assisted Reproduction: the means used for a double motherhood in Brazil (Review)

**Carolina Souza Delvali^1^, Bruna Santiago Martins^1^, Michelli Riviero Montaño^2^**

*^1^Centro Universitário das Faculdades Metropolitanas Unidas (FMU)*

*^2^IVI Global Education*

**Objective:** In this study we group and evaluate assisted human reproduction techniques in lesbian couples and the right to dual motherhood in Brazil. There are few materials found about this subject in a single article.

**Methods:** We conducted a literature review using Google Scholar, Scielo and PubMed as a means of research. We obtained articles for which we used only those dealing with lesbian couples and dual motherhood.

**Results:** With these data, we were able to assess how the choice of technique to be used is performed, how it is performed, average cost and risk factors associated with the procedures. We were also able to verify the laws that guarantee the right to civil registration in the name of the two mothers. From the least invasive to the most invasive techniques: Intrauterine Insemination (IUI) Home Insemination (CI), *In Vitro* Fertilization (IVF), Intracytoplasmic Sperm Injection (ICSI), Reception of Oocytes from the Partner (ROPA).

**Conclusion:** We conclude that individualized attention is necessary for the choice of technique, aiming to reduce the number of attempts, expenses and frustrations, thus contributing to the success of the procedure. Civil registration is guaranteed by law for all assisted human reproduction techniques addressed.

## P-89. The Lighting of the National Congress in the Color Orange A Milestone in the Assisted Human Reproduction Area

**Flávia Giacon^1^, Viviã de Sousa^2^**

*^1^Clínica de Reprodução Humana Mater Prime*

*^2^Revista Evolution*

**Objective:** The objective of this work is to report on the lighting project of the National Congress in orange, as an important milestone in the area of Assisted Reproduction in Brazil. This project, among its purposes, aims to draw attention to a public health issue, of rights guaranteed by law, such as the right of access to health and the right to family planning. We emphasize the importance of promoting the discussion about infertility, which is considered by the World Health Organization (WHO) as a disease and a public health issue that affects 6 to 8 million Brazilians, also presenting itself as a social problem, widely discussed in scientific articles.

**Methods:** The method chosen was to mobilize Parliament, to give visibility and voice to millions of Brazilians who suffer from infertility and who need reproductive treatments. The project was successful due to the essential support of the Parliamentary Advisory of the Brazilian Hospital Federation (FBH) and assistance from Federal Deputy Pedro Westphalen (PP/RS). The report can be used for indexing in future scientific works that will encourage research in the segment.

**Results:** The results of this article indicate that the lighting project of the National Congress in orange was a relevant milestone for the entire Human Reproduction sector and makes it possible to turn our eyes to this theme, opening space for important dialogues between the public and private spheres. It was an event that added up considerable efforts to alert and inform all civil society about the issue of infertility, the importance of early diagnosis, and the need to expand access to specialized services of Assisted Reproduction for those who wish to have children.

**Conclusions:** The results of this article emphasize the importance of achieving the Illumination of the National Congress in orange, as a milestone in the area of Human Reproduction. Illuminating the National Congress was a way of drawing the attention of all civil society to the cause of infertility and reproductive treatments. The actions are many and involve every segment in favor of the same objective, which is to raise awareness of infertility that affects a large number of Brazilians, in addition to encouraging the discussion about expanding access to reproductive treatments. Illuminating the National Congress was an achievement for the sector and a milestone for the area of Assisted Human Reproduction in Brazil.

## P-90. A first look: The secretome of embryo implantations in sequential culture medium

**Edson Guimarães Lo Turco^1^, Fernanda Rodrigues Bernarde^1^, Diogo Oliveira-Silva^2^, Maryke Wijma^3^, Ivan Henrique Yoshida^4^, Caio Parente Barbosa^5^, Fernando Prado Ferreira^6^**

*^1^Universidade Federal de São Paulo, Departamento de Urologia, Setor de Reprodução Humana*

*^2^Universidade Federal de São Paulo, Departamento de Química*

*^3^IonMedicine, Clinical Metabolomics*

*^4^Instituto Ideia Fértil de Saúde Reprodutiva*

*^5^Faculdade de Medicina do ABC*

*^6^Universidade Federal de São Paulo, Departamento de Ginecologia*

**Objective:** This study aimed to evaluate the effects of the embryo secretome and if it can predict the real potential of implantation.

**Methods:** Embryos of 25 patients were individually cultivated in sequential culture medium for 5 days, until they reached the blastocyst stage when the biopsies were performed. Controlled ovarian stimulation and dose adjustments were carried out according to the response of each patient, and there was an equal distribution between the groups. Fifty microliters of the culture media of the embryos that were transferred and cultured, were collected. All of the transferred embryos were classified as euploid by PGT-A. The samples were individually prepared for metabolite extraction and protein precipitation. Targeted metabolomics analyses were conducted with the aim of detecting and carrying out the absolute and/or relative quantification of 337 metabolites using liquid chromatography coupled to triple-quadrupole mass spectrometry. The top 15 metabolites with the highest VIP scores from the PLS-DA analyses were selected to construct ROC curves in order to illustrate the possibility of predicting implantation.

**Results:** After transferring 25 euploid embryos, 9 were not implanted and 16 were implanted. Comparing the quantitative targeted metabolomic results of the embryo culture mediums, increases in Cystine, Inosine and Methionine Sulfoxide were observed in euploid embryos that were not implanted, and increases in Guanosine, Citrulline, Lysine, Adenine and Glycine were observed in the euploid embryos that were implanted. Through the construction of ROC curves, we observed an AUC of 0.81. This result suggests that metabolomics analyses of thawed culture mediums of euploid embryos can be used as a complementary approach to predict the chance of the implantation of euploid embryos.

**Conclusions:** In summary, metabolomics analyses of euploid embryos and the creation of prediction models high accuracies, might be considered as tools to predict the chance of implantation chance. However, these findings need to undergo validation steps, and further studies are needed to understand the molecular mechanisms involved in embryo metabolism and implantation capacity.

## P-91. Pregnancy rates in oocyte receptors with or without embryo biopsy

**Raquel di Falco Cossiello^1^, Camila Kristine Gallo Liupkevicius^1^, Talita Biude Mendes^1^, Carlos Alberto Petta^1^**

*^1^Clínica Fertilidade & Vida*

**Objective**: Evaluate pregnancy and miscarriage rates in oocyte receptors with or without embryo biopsy.

**Methods:** A survey of 286 patient cycles (2017 to 2021), aged between 27 and 58 years, was carried out at the Fertilidade & Vida clinic. Receptors of fresh and frozen oocyte were included in this analysis. Only first transfers were included. Cases with severe male factor were excluded. From 286 cycles, 217 transfers were included in this study. The patient cycles with transfers were divided into 4 groups: G1 (n=35): Fresh oocytes with embryo biopsy; G2 (n=101): Fresh oocytes without embryo biopsy; G3 (n=17): Frozen oocytes with embryo biopsy and; G4 (n=64): Frozen oocytes without embryo biopsy. The biopsy was performed on the 5th day of the embryo development. The following rates were calculated and compared: Pregnancy - at least one beta hCG above 20mIU/mL; Clinical Pregnancy - presence of at least 1 yolk sac (YS) on ultrasound (US) at 7 weeks and positive fetal heartbeat; Biochemical pregnancy - beta hCG value above 20mIU/mL and absence of YS at 7 weeks US; Miscarriage - bleeding with elimination of the YS (confirmed via US) or absence of fetal heartbeat and; Ongoing Pregnancy - presence of at least 1 YS at 7 weeks US, positive fetal heartbeat and no miscarriage). Data were evaluated using Fisher’s test.

**Results:** There was no statistical difference in pregnancy, miscarriage and ongoing pregnancy rate when comparing the studied groups (Table 1).

**Conclusion:** We observed that donated oocytes, whether fresh or cryopreserved, have the same treatment success rates. In addition, the embryo biopsy did not bring any significant benefit or harm for the treatment of these patients. Therefore we concluded that treatment with donated oocytes has good success rates and that the status of these oocytes at the time of reception (cryopreserved or fresh), as well as embryo biopsy had no impact on the clinical performance of the procedures.

**Table 1:** Comparison between pregnancy rates in the treatment of oocyte receptors of fresh or cryopreserved oocytes with or without embryo biopsy.

PARAMETERS	FRESH DONOR OOCYTES (n=136)		FROZEN DONOR OOCYTES (n=81)	
	With biopsy (n=35)	With biopsy (n=101)	With biopsy (n=17)	With biopsy (n=64)
Female age^*^	41.29±3.92	41.47±5.32	42.24±2.93	42.53±5.08
**Pregnancy**	23 (65.71%)	75 (74.26%)	11 (64.71%)	47 (73.44%)
**Clinical pregnancy**	20 (57.14%)	65 (64.36%)	9 (52.94%)	38 (59.38%)
**Biochemical**	3 (8.57%)	10 (9.90%)	2 (11.76%)	9 (14.06%)
**Miscarriage**	0/23 (0.0%)	8/75 (10.67%)	1/11 (9.09%)	7/47 (14.89%)
**Ongoing pregnancy**	20 (57.14%)	57 (56.44%)	8 (47.06%)	31 (48.44%)

Analysis of Fischer-test.

* Values expressed as mean±standard deviation.

## P-92. Use of high dose Cetrorelix in the treatment of Ovarian Hyperstimulation Syndrome

**Lister de Lima Salgueiro^1^, Bernardo Lamounier de Moura^1^, Suelen Patricia Pinheiro Machado^1^, Adrielly Moura^1^, Edson Guimarães Lo Turco^2^**

*^1^ Clínica Fértilis*

*^2^ Universidade Federal de São Paulo, Disciplina de Urologia, Departamento de Cirurgia*

**Objective**: Analyze the clinical cases with Ovarian Hyperstimulation Syndrome (OHSS) data, when we used High Dose Cetrorelix (4 syringes at the same time), as a treatment, plus symptomatic medications.

**Methods**: A retrospective observatory study, with the evaluation of, clinic evolution data. fifty- five patients with OHSS were treated with high-dose Cetrorelix, plus symptomatic medications.

**Results**: Of 55 patients with OHSS, submitted to the protocol, (more than 15 eggs or more than 3000 pg/ml), 13 patients (24.0%), did not have symptoms, 30 (55.0%), had a 4 days period with symptoms, 8 (14.0%), had 7 days with symptoms, and 6 (7.0%), had 10 days with symptoms. Sixteen patients 16 (29%) were submitted to a Culdocentesis in the clinic, due to the ascites, and there were no hospitalizations. The OHSS was classified as mild, in 39 cases (70.0%), moderate in 16 (29.0%), and there were no severe or critical cases. Concerning age, 30 (55%) had less than 35 years, 20 (36%) had between 35 and 39 years, and 5 (9.0%) had more than 40 years. In 33 cases (60.0%), were diagnosed with PCO, 11(20%) with Endometriosis, and 13 (23.6%) with low testosterone levels.

**Conclusions:** The OHSS is a clinical entity, with an iatrogenic origin, in almost all cases. Is classified, into mild, moderate, severe, and critical levels. The treatment varies, with the severity of the case; it could be on an outpatient basis, or hospitalization, in the Intensive Care Unit, depending on the case. The treatment with high dose Cetrorelix, just after egg collection, showed efficiency in the control of OHSS. The action of the Cetrorelix is fast (24h), but a higher dose seems to have a more drastic effect on the hormonal block. The treatment with Cetrorelix in high doses, is made on an outpatient base, avoids hospitalizations, shortens the time, diminishes the level of the symptoms, and could be an alternative to conventional treatments.

## P-93. Impact of the FMR1 CGG repeat lengths in women with alleles classified within the normal range on the ovarian reserve and retrivied oocytes: A pilot study

**Ana Carolina Vasconcelos Nunes^1^, Julia Azevedo de Sá Nunes^1^, Denise Maria Christofolini^1^, Caio Parente Barbosa^1^, Bianca Bianco^1^**

*^1^Faculdade de Medicina do ABC/Centro Universitário FMABC*

**Objective:** Infertility affects about 20% of couples of reproductive age. Genetic variants that may be associated with infertility, including the dynamic mutation in the 5´untranslated region of the *FMR1* gene. Previous studies suggested that the premutation (55-200 CGG repeats) can interfere in the prenatal development of the oocyte pool by reducing the number of viable oocytes. Also, premutation is associated with irregular menstrual cycles, infertility, increased risk of early ovarian failure, impairing the reproductive outcomes. However, the risk of reduction of ovarian reserve associated with the number of *FMR1* CGG repeats is still unclear. We aimed to evaluate the impact of the number of *FMR1* CGG repeats in infertile women and alleles classified within the normal range on ovarian reserve, namely FSH, AMH, and antral follicle count (AFC) and the number of retrieved oocytes.

**Methods:** A cross-sectional study was carried out comprising 61 infertile women undergoing *in vitro* fertilization treatment. Inclusion criteria were women aged ≤ 40 years old, FSH (≤12.0 IU/L), TSH (≥0.5 ≤ 4 IU/L), serum prolactin levels (<25 ng/mL), body mass index >18.5<30, ovulatory cycles (25-35 days), the presence of both ovaries without any malformations, 46,XX karyotype and < 45 CGG repeats alleles . FSH and AMH serum levels were measured in the follicular phase of the menstrual cycle, as well as the AFC immediately before controlled ovarian stimulation (COS). COS was performed using exogenous recombinant FSH (rFSH) at a daily fixed dose of 150/200 UI , starting on the second or third day of the menstrual cycle. The *FMR1* CGG repeat length was investigated by polymerase chain reaction followed by capillary electrophoresis, and classified as low CGG repeats (<26), normal (26-34) and high (35-44). Patients were grouped according to allele combinations of low-low, low-normal, low-high, normal-normal, normal-high, and high-high. The statistical analysis considered only the genotypes low-low, low-normal, and normal-normal.

**Results:** The median age of the patients was 33 (32-35) years, FSH 7.0 (6.5-7.4) mUI/mL, AMH 1.6 (1.2-2) ng/mL, AFC 7 (7-9.6) and retrieved oocytes 7 (5-8). Considering the alleles of the *FMR1* gene, 37.7% (n=23) of the women carried alleles classified as low-low, 39.4% (n=24) low-normal, 1.6% (n=1) low-high, 16.4% (n=10) normal-normal, 1.6% (n=1) normal- high and 3.3% (n=2) high-high. Women carrying alleles classified as low-low presented significantly lower FSH levels than those carrying normal-normal alleles (6.4 mIU/mL versus 8.0 mIU/mL, p=0.006). AMH levels, antral follicle count and number of retrieved oocytes were not different among groups.

**Conclusion:** The low number of *FMR1* CGG repeats impacted FSH levels, but not the other markers of ovarian reserve and oocyte retrieved in infertile women

**Grants:** PIBIC-CNPq and Fundação de Amparo a Pesquisa do Estado de São Paulo.

## P-94. The average number of oocytes to obtain a euploid blastocyst

**Jhuly Laurentino Nunes^1^, Lucileine Keico Nishikawa^1^, Ianae Ichikawa Ceschin^1^, Paulina Alejandra Santander Perez^1^, Alvaro Pigatto Ceschin^1^**

*^1^Feliccità Instituto de Fertilidade*

**Objective**: Estimate the average number of injected oocytes needed to obtain a euploid blastocyst, based on maternal age.

**Methods**: A retrospective analysis was carried out in the period from 2020 to 2022, of the cases of embryo biopsy in the ART center. Embryo biopsy cases that had at least one embryo biopsied were included in the study, regardless of the factor and period of infertility. Patients were allocated according to age into the following groups: Group A - <35 years; Group B - 36-39 years old; Group C - >40 years. The number of injected mature oocytes, the number of blastocysts biopsied and the number of euploid embryos per group was used to carry out the calculations.

**Results**: Intracytoplasmic sperm injection (ICSI) was performed in 384 mature oocytes. In the end, 130 blastocysts were biopsied, resulting in 46 euploid embryos. The distribution of patients between the groups was: Group A - 10.8%; Group B - 34.9%; Group C - 54.3%. Regarding the number of oocytes injected, the rate of blastocyst formation was respectively 45.65% (Group A), 36.74% (Group B), and 28.80% (Group C). The percentage of euploid embryos, according to the age of the patients, was 80% for Group A, 38.8% for Group B, and 14.5% for Group C. Based on the results, the mean number of oocytes injected to obtain a euploid embryo was: Group A - 2.7; Group B - 7.42; Group C - 21.87. The mean number of blastocysts biopsied to achieve a euploid result was 1.23 for Group A, 2.57 for Group B, and 6.87 for Group C.

**Conclusion**: To obtain a euploid embryo at the end of an IVF cycle, an average of 2.7 oocytes are needed for patients up to 35 years of age, 7.42 oocytes for patients between 36-39 years, and 21.87 oocytes for patients above 40 years. This paper shows an estimate of the number of gametes to obtain a euploid embryo, helping with relevant information in clinical practice.

## P-95. Premature Progesterone Elevation during the Final Follicular Phase in infertile patients undergoing controlled ovarian stimulation for *In Vitro* Fertilization: A Multivariable Analysis for associated factors

**João Sabino Cunha-Filho^1^, Tatiane Souza^1^, Rita Chapon^1^, Rafaela Donato^1^, Camila Bessow^1^, Vanessa Genro^1^**

^1^ Centro de Reprodução Humana INSEMINE

**Objective**: To investigate some demographic, reproductive and hormonal variables that could be associated with an enhanced progesterone secretion during the late follicular phase in infertile patients undergoing controlled ovarian stimulation for IVF.

**Methods:** We prospectively studied 117 infertile patients undergoing the first IVF cycle using four different controlled ovarian stimulation protocols (FSH, HMG, FSH + LH and Long-acting FSH), during 2021-22. Patients were divided into two different groups according to the serum progesterone level on HCG day. Group 1 was composed by patients with serum progesterone level < 1.5 ng/ml (n=74) and Group 2 composed by those patients who presented serum progesterone > 1.5 ng/ml (n=43). All of the studied patients met the following inclusion criteria: (i) both ovaries present, with no morphological abnormalities (such as cysts, endometriomas, etc.), no history of past surgery and adequately visualised in transvaginal ultrasound scans; (ii) regular menstrual cycle lengths ranging between 25 and 35 days; (iii) no current or past diseases affecting ovaries or gonadotrophin or sex steroid secretion, clearance or excretion; (iv) no clinical signs of hyperandrogenism; (v) BMI ranging from 16 to 30 kg/m^2^. Progesterone, estradiol and LH were collected on HCG-day and analysed using chemiluminescence immunoassay. We compared age, AFC, AMH, number of collected oocytes, mature oocytes, TOP embryos (A+B on day 3), LH, estradiol and the number of follicles > 17 mm on HCG day. Our data were analyzed using parametric and non-parametric correlation tests and multivariable analysis (Linear regression). P was considered significant when < 5%.

**Results:** Mean age (36.4±4 x 35.0±4) and serum LH during the late follicular phase (2.9±4 x 1.8±1) were not different between both groups (*p*>0.05).

However, Antral Follicle Count (8±5 x 12±5), AMH (1.9±1.7 x 2.9±2.2), number of collected MII oocytes (5.±4 x 8±4) and number of embryos (4.5±4 x 7.0±4), serum estradiol levels on HCG-day (2042±1596 x 4187±3039) and number of follicles > 17 mm (3±2 x 5±3) were significantly (higher in Premature Progesterone elevation) different between both groups (P<0.05). Patients receiving daily FSH administration also presented a higher risk of Premature Progesterone Secretion (*p*=0.001) in comparison to HMG, long-acting FSH or FSH+LH. Furthermore, using a logistic regression analysis considering estradiol, AFC and AMH as covariates to Serum Progesterone >1.5ng/ml, only serum estradiol on HCG-day was a prognostic factor (*p*=0.004). Moreover, Premature Progesterone elevation was not associated with TOP embryo development 0.46 (-0.45-1.31).

**Conclusion:** Premature Progesterone Elevation is related to the kind of prescribed gonadotrophin and is more prevalent in higher responders (elevated AMH/AFC). Serum estradiol levels were also increased in those patients. On the other hand, Premature Progesterone increase was not linked to embryo development.

## P-96. Final Follicular Phase LH, estradiol and progesterone secretion in Long-acting FSH versus daily FSH administration for controlled ovarian stimulation for *In Vitro* Fertilization

**João Sabino Cunha-Filho^1^, Tatiane Souza^1^, Rita Chapon^1^, Rafaela Donato^1^, Camila Bessow^1^, Vanessa Genro^1^**

*^1^Centro de Reprodução Humana INSEMINE.*

**Objective**: To investigate the serum progesterone, LH and estradiol levels during the late follicular phase in infertile patients undergoing controlled ovarian stimulation for IVF, using two different FSH protocols (long-acting versus daily).

**Methods:** We prospectively studied 37 infertile patients undergoing the first IVF cycle using two different protocols for controlled ovarian stimulation: group 1 (n=20) was composed of patients receiving daily FSH and GnRH antagonist and Group 2 of long- acting FSH and GnRH antagonist (n=17), during 2022. All of the studied patients met the following inclusion criteria: (i) both ovaries present, with no morphological abnormalities (such as cysts, endometriomas, etc.), no history of past surgery and adequately visualised in transvaginal ultrasound scans; (ii) regular menstrual cycle lengths ranging between 25 and 35 days; (iii) no current or past diseases affecting ovaries or gonadotrophin or sex steroid secretion, clearance or excretion; (iv) no clinical signs of hyperandrogenism; (v) BMI ranging from 16 to 30 kg/m^2^. Progesterone, estradiol and LH were collected on HCG-day and analysed using chemiluminescence immunoassay. We also compared some important reproductive outcomes. Our data were analyzed using parametric and non-parametric correlation tests and multivariable analysis (Linear regression). *p* was considered significant when < 5%.

**Results:** Mean age (35.7±5 x 37.1±4), Antral Follicle Count (10±6 x 8.3±4), AMH (2.71±2.6 x 2.14±1.4), number of collected MII oocytes (9±6 x 7.5±6) and number of embryos (5.7±3 x 4.3±4) were not different between control and endometriosis groups, respectively (*p*>0.05). Furthermore, serum estradiol (2075±1970 x 2557±2295) and LH (2.4±5 x 3.0±4) levels did not differ also. Therefore, patients receiving daily FSH presented higher serum progesterone levels compared to long-acting FSH (1.24±0.6 x 0.86±0.4, *p*=0.038).

**Conclusion:** Controlled ovarian stimulation using daily FSH administration increased serum progesterone levels during the late follicular phase. Those patients should be carefully monitored and this could be detrimental to embryo implantation.

## P-97. Assisted reproduction treatments and the influence of religion

**Mariane Cristina Carlucci Molina Félix^1^, Fernanda Kunrath Robin^1^, Raquel Winter^1^, Nilo Frantz^1^**

*^1^Nilo Frantz Medicina Reprodutiva*

**Objective:** The objective of this work is to know the influence of religions on the diagnosis of infertility and assisted human reproduction (AHR) treatments.

**Methods:** A review of the last five years’ worth of literature was done. The search was performed in the Virtual Health Library (BVS) database with the descriptors “religion” and “infertility.” Portuguese, English, and Spanish-language articles were taken into consideration.

**Results:** For women living in societies where pro-natalist culture is prevalent and where social policies support an increase in birth rates, infertility becomes a significant source of suffering. Because their expectations for starting a family are not met, these women end up separating themselves from religion and religious practices, which forces the woman into isolation. Since faith and prayer are always encouraged, it is also possible to consider that this departure is a result of the judgment regarding participation in assisted reproductive treatments. Infertility (still taboo), isolation, stressful situations made worse by the absence of children, guilt, and a slight pressure to leave a wife are the emotions that are most frequently mentioned. The stages of treatment also cause psychological and emotional stress. When evaluating the effect of the religious group on the ICSI results, there was a significant increase in the fertilization rate, number of quality embryos, and pregnancy rates, results that were verified in the participants who declared themselves to be spiritualists and evangelicals; participants who declared themselves to be Catholics, or of alternative religions, or without a religion, had a worse result. Nursing stands out as the field capable of carrying out the approach in a holistic manner and with consideration for the patient’s history, beliefs, and religious identity.

**Conclusion:** With these findings, we can see how the couple undergoing assisted reproduction treatments’ stress levels are raised by the cultural elements that permeate religion. When a person wants to seek treatment and fulfill their parental dream, their religion does not stand in the way. But because of societal misconceptions about the woman, the infertile couple, and their use of assisted reproductive technologies, there is social distance between them. More research is required to more accurately determine the impact of religion on patients undergoing assisted reproductive technologies and on the outcomes of treatments.

## P-98. Evaluation of oocyte-embryonic competence and results *in vitro* fertilization treatment in patients with reduced ovarian reserve

**Mila Harada Ribeiro Cerqueira^1^, Vanessa Cristina Freitas Moreno^1^, Vanessa Nayane Pino Perez^1^, Gustavo Cerqueira e Silva^1^, Kazue Harada Ribeiro^1^**

*^1^Clinifert Centro de Reprodução Humana*

**Objective:** To evaluate the association between ovarian reserve markers and oocyte-embryonic competence, comparing the rates of fertilization, blastulation, pregnancy and abortion in patients with reduced and normal ovarian reserve.

**Methods:** Retrospective survey was conducted from January 2020 to March 2022, including a total of 446 ICSI cycles, divided into two groups: group 1 with 236 patients with decreased ovarian reserve (DOR) and group 2 with 210 patients with normal ovarian reserve (Normal). Each group was subdivided into 2 subgroups according to age below 37 years and 37 years or above. Ovarian reserve parameters were based on Bologna criteria: anti-Mullerian Hormone (AMH) <1.1ng/mL and antral Follicle Count (AFC) <7, considering AFC as the main cut-off point for the DOR group. The groups were compared for the number of oocytes retrieved, inseminated Metaphase **II** (MII), fertilization and blastulation rate, blastocyst transfers, clinical pregnancy outcomes and miscarriage. Statistics were performed using Factorial Anova analysis of variance, Pearson and Fisher correlation coefficient and chi-square test.

**Results:** The mean age of women in the DOR group in this study was 38 years old and in the Normal group, 36 years. In the DOR group, 35.6% were <37 years and 64.4% = or >37 years and in the Normal group, 52.4% and 47.6%, respectively. In the DOR group, 113 (47.8%) blastocyst transfers were carried out and in the Normal group, 156 (74.2%). In the DOR group, 951 oocytes were retrieved, 741 MII (77.9%) and 539 fertilized (72.7%), with a mean of 2.2 fertilized per patient. The blastulation rate was 69% in women <37 years old and 56% in women = or >37 years old. In the Normal group, 2,711 oocytes were retrieved, 1,920 MII (70.8%) and 1,446 fertilized (75.3%), with a mean of 6.7 fertilized per patient. The blastulation rate in women <37 years was 58.1% and 55% in older women. There was no significant difference in both groups in terms of fertilization rates when compared by age group (*p*=0.315), however, when compared by groups, the DOR group had a lower fertilization rate than the normal group (p=0.033). Regarding the blastulation rate, it was found that there was a negative correlation only in the elderly women in the DOR group (r-0.269, *p*<0.001), which did not occur in the Normal group (p=0.603). Pregnancy rates in the DOR and Normal groups in women < 37 years old were 57% and 60% respectively and 42% and 52% in older women. Although pregnancy rates were higher in the Normal group, there was no significant difference (*p*=0.326). Comparing the two groups, despite the variation in abortion rates in women = or >37 years, respectively 10% and 5%, it was found that there was no statistically significant difference (*p*=0.572). In younger women, regardless of ovarian reserve, there was no discrepancy in abortion rates, with results of 7% in the DOR group and 6% in the Normal group.

**Conclusion:** Patients with reduced ovarian reserve had a lower fertilization rate than those with normal reserve. However, there was no difference in blastulation rates, except when associated with advanced age. Regarding pregnancy and miscarriage rates between the two groups, it is suggested that there is no association between ovarian reserve markers and oocyte or embryo quality. However, age and the availability of oocytes and embryos seem to be associated with the rate of embryo transfer and constitute an important predictive factor of success in assisted reproduction treatments.

## P-99. Comparison of reproductive outcomes between OHSS versus non-OHSS patients submitted to frozen embryo transfer

**Lisiane Knob de Souza^1^, Bruna Campos Galgaro^1^, Luiza da Silva Rodrigues^1^, João Sabino Lahorgue da Cunha Filho^1^**

*^1^Insemine*

**Objective:** To compare the pregnancy rate of the first frozen embryo transfer of freeze- all cycles by ovarian hyperstimulation syndrome (OHSS) or other reasons.

**Methods:** This retrospective study analyzed 127 first frozen embryo transfers from January 2020 to July 2022 of patients who went under freeze-all cycle by ovarian hyperstimulation syndrome (OHSS) or other reasons. Clinical variables age, anti-mullerian hormone (AMH) and infertility causes and laboratory variables number of retrieved oocytes, number of MII and pregnancy rate were compared between the groups OHSS and other reasons. The statistical analysis was performed using qui-square, Mann- Whitney and T-Student tests and results were described as percentage, median [25th - 75th percentiles] and mean±DP respectively.

**Results:** Patients were divided into two groups: 73 patients in the OHSS group and 54 patients in the other reasons group (endometrium, elevated P4, COVID-19). The mean age was 33.19±4.22 and 36.09±4.95 (*p*<0.001), AMH of 4.87 [3.05-7.18] and 2.86 [1.47-3.57] (*p*<0.001) for the OHSS group and the other reasons group respectively. Infertility causes were not statistically significant between groups (*p*=0.052), being multiple causes as the major cause in both groups (38.3% and 42.6%), followed by male factor (31.6% and 20.7%). The laboratory parameters analysis showed a statistically significant difference between the two groups regarding number of retrieved oocytes (13 [9-18] and 7.5 [5-10]; *p*<0.001), number of MII (10 [8-15] and 6 [4-8]; *p*<0.001) and pregnancy rate (45% vs 24%; *p*=0.014). Fertilization rate was also analyzed, 78% to the OHSS group and 82% to the other reason group (p=0.07).

**Conclusion:** Pregnancy rate was higher in the OHSS group indicating that the freeze-all protocol is not harmful to this group of patients. Hence, this approach may be a better conduct for good responders.

## P-100. Chronic endometritis in patients with implantation failure in *In Vitro* Fertilization cycles

**Suélen Patrícia Pinheiro Machado de Lima^1,2^, Lister de Lima Salgueiro^1,2^, Adrielly B. Silva^1^, Bernardo Rodrigues Lamounier de Moura^1,2^**

*^1^Clínica Fértilis de Medicina Reprodutiva*

*^2^Embriólogica Assessoria e Educação em Reprodução Assistida*

**Objective:** Successful embryo implantation is a complex process that involves a multitude of factors and requires a healthy dialogue between the embryo and the endometrium. Endometrial changes, such as endometritis, can be one of the causes of non-implantation and, consequently, of IVF failure. There are different types of microorganisms, which can lead to endometrial change or inflammation. Depending on the type of microorganism, some antibiotics may be used in the first option, and in other cases other types of antibiotics are used. These can impair endometrial receptivity, leading to implantation failures and recurrent miscarriages. This study aims to analyze the results of 55 patients who had implantation failure after the transfer of embryos of good morphological quality and who underwent endometrial biopsy.

**Methods**: In this study, 55 patients aged between 19 and 44 years who had implantation failure after the transfer of good quality embryos were considered. The patients underwent endometrial biopsy and were diagnosed with a high prevalence of Mycoplasma and Ureaplasma sp., in addition to the presence of common bacteria (such as Streptococcus sp., Escherichia coli, Enterococcus faecalis, Klebsiella pneumoniae, Staphylococcus sp. and Corynebacterium), with lower prevalence, Chlamydia trachomatis and Neisseria gonorrhoeae were found, evidencing the presence of ENDOMETRITIS by anatomopathological study. Patients underwent treatment with broad-spectrum antibiotics for 15 days and, at the end of treatment, a second endometrial biopsy was performed to control the effectiveness of the treatment performed. Patients with a normal histopathological pattern were referred for embryo preparation and transfer in the next cycle and patients with an irregular histopathological pattern, with persistent endometritis, were referred for a new different antibiotic treatment for 15 days. At the end, they returned for a new endometrial biopsy to control the effectiveness of the treatment and subsequent preparation and transfer of embryos in a new cycle after normal histopathological results.

**Results**: Of the 55 patients diagnosed with endometritis and undergoing treatment, 39 (70.9%) patients returned for the second biopsy and 16 (29.0%) patients did not return after the proposed treatment. Of the patients who returned, 23 (58.9%) had a normal histopathological result after a new endometrial biopsy and continued with a new IVF cycle or embryo preparation and transfer. The 16 (41.0%) patients with altered results underwent a new course of treatment with new antibiotics and returned for a new endometrial biopsy and obtained a normal histopathological result to then proceed to a new cycle of IVF or preparation and transfer of frozen embryos. Of the 23 patients, after clinical treatment and the first normal control biopsy, they performed another IVF attempt or frozen embryo transfer, where 13 (56.5%) became pregnant and 10 (43.4%) did not. On the other hand, patients who had persistent endometritis and a new course of treatment was necessary to normalize the histopathological pattern, 7 (43.7%) became pregnant and 9 (56.2%) did not become pregnant. The mean age of patients was 32 years in the first group and 33 years in the second.

**Conclusion:** The performance of endometrial biopsy in cases with implantation failure proved to be important for the diagnosis of Endometritis and the clinical treatment approach proved to be effective for Endometritis and seems to increase the efficiency of *In Vitro* Fertilization results, as in total 51.3% of patients became pregnant after diagnosis and proposed treatment. Patients with persistent endometritis who required two cycles of treatment had a worse pregnancy prognosis than patients in the first group. These data show the importance and influence of the diagnosis and treatment of endometritis and we suggest further studies that can corroborate the data presented here.

## P-101. Evaluation of blastocyst formation and quality between day three sibling embryos cultured with continuous versus sequential commercial mediums

**Bruna Campos Galgaro^1^, Lisiane Knob de Souza^1^, Luiza da Silva Rodrigues^1^, João Sabino Lahorgue da Cunha Filho^1^**

*^1^Insemine*

**Objective:** To compare the blastulation rate and good quality blastocyst rate of day three embryos cultured with continuous versus sequential medium.

**Methods:** This is a prospective cohort study with 107 sibling cleavage embryos of IVF cycles from January to July 2021. Embryos were cultured in the same medium until day 3. On day 3, the sibling embryos were classified and equally divided into two groups: sequential or continuous medium. The number and quality of blastocysts were evaluated on day 5/6. Blastocysts classified as AA,AB,BA and BB according to Gardner’s scale were considered as good quality blastocysts. The culture conditions were applied following the manufacturer’s descriptions and statistical analysis were evaluated by qui-square test.

**Results:** The mean age of female patients who went under IVF cycles was 36.1 years. Of 107 cleavage embryos enrolled, 83 resulted in blastocysts, with a total blastulation rate of 77.57%. There was no statistical difference between continuous, 83.33%, and sequential medium, 71.70% (*p*=0.149). Regarding blastocyst classification, the formation of good quality blastocysts from the totality of day three embryos was 37.04% and 20.75% (*p*=0.063) for the continuous and sequential systems respectively, whereas including only great cleavage embryos i.e. 8-cell, symmetric and grade I fragmentation it was 37.5% and 60.0% (*p*=0.210) for sequential and continuous mediums respectively.

**Conclusion:** No statistical differences were found in blastulation or good blastocyst rates between the tested mediums. Therefore, under the optimal conditions, it is possible to achieve a good blastulation rate in both culture systems. However, because of the lack of a standard protocol, each IVF laboratory must create its own protocol based on their results and Key Performance Indicators (KPIs).

## P-102. Effect of smooth endoplasmic reticulum aggregates in the oocyte cytoplasm in IVF

**Bruna Campos Galgaro^1^, Lisiane Knob de Souza^1^, Luiza da Silva Rodrigues^1^, João Sabino Lahorgue da Cunha Filho^1^**

*^1^Insemine*

**Objective:** To evaluate the effect of the presence of smooth endoplasmic reticulum aggregation in the oocyte cohort on laboratory parameters.

**Methods:** A retrospective analysis enrolling 267 ICSI cycles from January 2021 to July 2022 was conducted. Clinical and laboratory parameters were compared between cycles with and without the presence of smooth endoplasmic reticulum aggregation (SER+) in the oocyte cohort. Among clinical factors, age, anti-Mullerian hormone (AMH), E2 on the hCG-administration day and cause of infertility were evaluated. Regarding laboratory parameters, the number of follicles, oocytes retrieved and MII oocytes, as well as fertilization, good quality cleavage embryos, 3PN and oocyte atresia rates were investigated. Day three embryos grade A (eight cells, symmetric and a maximum of 10% of fragmentation) and grade B (7 cells, symmetric and a maximum of 10% of fragmentation) were considered good quality embryos. The statistical analysis was performed using qui-square and Mann-Whitney tests and results were described as percentage and as median and 25th - 75th percentiles respectively.

**Results:** Of the treatments included, 23 (9.5%) were SER+cycles. The age of patients was not significantly different for SER+ and SER- cycles (34 [30-38] and 36 [34-39] *p*=0.063). Multiple infertility causes were the prominent reason in both groups (52.1% vs 47.1%) followed by male factor (17.3% vs 17.6%) and anatomic female factor (13% vs 12.7%) (*p*=0.884). No statistical difference was found for AMH (1.35 [1.17-3.39] and 1.5 [0.82-2.86] *p*=0.509), E2 (1844 [1565-2613] and 1742 [972-3480] *p*=0.505), and number of follicles (9 [6.5-13.5] and 8 [4.75-11] *p*=0.176). In the presence of SER+ oocytes, the median number of oocytes retrieved was statistically higher (9 [4.5-13.5] and 5 [3-9] *p*=0.048), whereas the number of MII oocytes was not (6 [3.5-11] and 5 [2-8] *p*=0.059). Furthermore, the fertilization rate was lower when SER aggregates were present in the oocyte cohort (71.14% vs 80.39% *p*=0.007), although atresia rate (6.04% vs 5.23% *p*=0.67) and good quality cleavage embryo rate (52.83% vs 43.81% *p*=0.07) were not statistically different. Interestingly, the rate of 3PN embryos was increased in SER+ (5.37%) against SER- group (0.58%) *p*<0.001.

**Conclusion:** SER+ cycles are quite common and the presence of SER aggregates in the oocyte cohort was associated with increased number of oocytes retrieved. Moreover, although lower normal fertilization and higher 3PN rates in SER+ group, this appears to not influence the quality of day three embryos.

## P-103. Testosterone and antioxidants supplementation before controlled ovarian stimulation for *in vitro* fertilization: a prospective non-randomized study

**João Sabino Cunha-Filho^1^, Tatiane Souza^1^, Rita Chapon^1^, Rafaela Donato^1^, Camila Bessow^1^, Vanessa Genro^1^**

*^1^Centro de Reprodução Humana INSEMINE, Porto Alegre, Brasil*

**Objective:** To investigate the effect of testosterone and antioxidants supplementation in the ovarian follicular response in infertile patients undergoing controlled ovarian stimulation for IVF in patients with AMH <1.0ng/ml.

**Methods:** We prospectively studied 64 infertile patients undergoing an IVF cycle. The study group was composed of 19 patients using transdermal Testosterone 5.5mg + L- carnitine, DHEA, Myoinositol, ac. folic, vitamins E and C daily for 30 days before controlled ovarian stimulation (HMG + GnRH antagonist), during 2021-22.Control Group (n=45) was composed of infertile patients undergoing IVF at the same time with the same protocol, except for the administration of pre-treatment with testosterone and anti-oxidants. All of the studied patients met the following inclusion criteria: (i) both ovaries present, with no morphological abnormalities (such as cysts, endometriomas, etc.), no history of past surgery and adequately visualised in transvaginal ultrasound scans; (ii) regular menstrual cycle lengths ranging between 25 and 35 days; (iii) no current or past diseases affecting ovaries or gonadotrophin or sex steroid secretion, clearance or excretion; (iv) no clinical signs of hyperandrogenism; (v) BMI ranging from 16 to 30 kg/m^2^; vi) AMH measured in the lasts 6 months <1.00 ng/ml. Our primary endpoint was the number of follicles that reached 17mm on HCG day. Moreover, we compared also the groups: age, AFC, AMH, number of collected oocytes, and mature oocytes. Our data were analyzed using non-parametric correlation tests and multivariable analysis (Logistic/Linear regression). *p* was considered significant when < 5%.

**Results:** Values were expressed as median and range and presented as study versus control groups, respectively: age (37 [33-43] x 37 [29-43]), AFC (5 [2-14] x 5 [0-10]), AMH (0.47 [0.12-1.00] x 0.54 [0.05-1.00]), number of collected oocytes (3 [1-12] x 3 [0-11]) and mature MII (3 [0-10] x 3 [0-12]) were not different between both groups (*p*>0.05). Furthermore, the number of follicles > 17 mm on HCG day was also similar between both groups (2 [0-7] x 2 [0-4]), *p*=0.306. In addition, using multivariable analysis, pre-treatment with testosterone and anti-oxidants did not change the studied reproductives outcomes (*p*>0.05).

**Conclusion:** Administration of testosterone and anti-oxidants in infertile patients with reduced serum AMH levels (<1.00 ng/ml) undergoing IVF did not increase or modify the number of mature follicles, nor the number of mature oocytes.

## P-104. Desogestrel is a new option for LH suppression during controlled ovarian stimulation for oocyte vitrification: prospective cohort study

**Rafaela Donato^1^, Camila Bessow^1^, Vanessa Genro^1^, Rita Chapon^1^, Tatiane Souza^1^, João Sabino Cunha-Filho^1^**

*^1^Centro de Reprodução Humana Insemine*

**Objective:** Progesterone compounds are currently wide-used during controlled ovarian stimulation for oocyte freezing for LH surge suppression. Desogestrel is a synthetic oral progesterone, used primarily in contraceptives. The main advantage of this progestin is the price and facility. Therefore, the aim of this study is to describe for the first time the usefulness of desogestrel during controlled ovarian stimulation for patients submitted to social oocyte vitrification.

**Methods:** A retrospective cohort study was performed during the period of 2019-2021 including 75 patients who submitted to oocyte freezing and 173 women matched by age as the controls (patients submitted to IVF for the first time). All patients received the same gonadotropin regimen (recombinant FSH). The final oocyte maturation also had the same protocol for both groups (recombinant hCG and GnRH analogue agonist). Patients from the control group used a flexible GnRH antagonist for LH suppression, and patients in the study group utilized desogestrel 75mcg PO bid (starting from the beginning of gonadotropin to hCG/agonist day). The primary endpoint was the number of collected MII.

**Results:** Age, baseline antral follicle count and anti-Mullerian hormone were similar between both groups as demonstrated in Table 1. However, the controlled ovarian stimulation length was shorter in the control group and those patients utilised less gonadotropin than the desogestrel group. Finally, the number of collected and MII oocytes was the same between both groups.

**Conclusions:** Desogestrel is a new option for patients seeking oocyte freezing during controlled ovarian stimulation, this drug is cheap and easy to use during this process.

**Table:** 

	Desogestrel (n=75)	Control (n=173)	statistics
Age (y)	36 (28-43)	36 (23-45)	0.793
AMH (ng/pg)	1.00 (0.01-16.00)	1.5 (0.05-7.95)	0.540
AFC	9 (2-21)	8 (2-23)	0.741
total dose gonadotropin (IU)	2933±785	2736±745	0.047
days of induction	11.0±1.6	10.4±1.6	0.044
number of oocytes	8 (1-20)	6 (1-33)	0.293
number of MII oocytes	6 (1-19)	5 (1-27)	0.156
FORT	52%	45%	0.217

## P-105. A profile of the professional nurse in assisted human reproduction services

**Raquel Adriana Winter^1^, Fernanda Kunrath Robin^1^, Catia Aguiar Lenz^2^, Mariane Cristina Carlucci Molina Félix^1^, Nilo Frantz^1^**

*^1^Nilo Frantz Medicina Reprodutiva.*

*^2^ Universidade Feevale*

**Objective:** The term assisted human reproduction (AHR) is used to describe methods for treating infertility in couples that involve manipulating at least one gamete. This is an excellent opportunity for the professional nurse to improve their technical and scientific knowledge due to their involvement with the processes and monitoring of the patient/couple diagnosed with infertility. Objective: The purpose of this study is to define the professional nurse profile in assisted human reproduction (AHR) services.

**Methods:** This is qualitative research with a descriptive and exploratory design. Information was gathered in September and October 2021 by applying for interviews with the participants. Six nurses were interviewed, corresponding to 100% female. They are professionals working in Assisted Human Reproduction Clinics in the states of Distrito Federal, Minas Gerais, São Paulo and Rio Grande do Sul, where only one works in the public network and the others only in the private network. The interviewees are all under the age of 45 and between the ages of 30 and 35. The average age of the adult population is between 30 and 35 years old. The typical length of employment at RHA Clinics ranges from six to ten years. One interviewee works for less than five years, two for between 11 and 15, and three for between six and ten years.

**Results:** Six nurses, working in AHR Clinics, from the public (16.67%) and private (83.33%) networks were interviewed. This is a highly specialized population with a small number of members available for study. The interviewees’ ages range from 30 to 35 on average.. The typical length of employment ranged from six to ten years. Congresses, seminars, classes, scientific meetings, improvement programs, webinars, books, and, primarily, scientific articles were the most frequently cited updating methods. As for academic production in RHA, three nurses reported not having publications in magazines or newspapers in the area. One nurse claims to have published an article in 2011 and to have had two posters approved at events in 2016 and 2017. One nurse has an unrelated publication in the RHA in the year 2017. And, a nurse teaches classes at congresses at RHA annually since 2015. Four interviewees stated they belonged to the Brazilian Society of Assisted Reproduction (SBRA), three said they belonged to the Brazilian Society of Human Reproduction (SBRH), two said they belonged to the Latin American Network of Assisted Reproduction (REDLARA), and a nurse said she was not connected to any RHA institutions. The analysis of the information from the questionnaires took place through content analysis. The analysis produced three thematic categories, including the career trajectory of the professional nurse in the assisted human reproduction service, the experiences and applications of the systematization of nursing care in RHA, and the professional influences and job satisfaction in RHA.

**Conclusion:** It was discovered that the identification of this professional’s role is essential due to the fact that academic nursing education in Brazil does not offer a specific theoretical foundation in RHA and that there aren’t many courses that specialize in this field. The nurse has a fundamental role in the care and monitoring of patients/couples who seek reproductive medicine services, being the only professional present at all stages of care, in addition to playing a strategic role in the administrative and organizational management of the care flow. According to the sentiments expressed by every interviewee, it is extremely satisfying that the significance of professional training and qualification on this topic is highlighted.

